# The real climate and transformative impact of ICT: A critique of estimates, trends, and regulations

**DOI:** 10.1016/j.patter.2021.100340

**Published:** 2021-09-10

**Authors:** Charlotte Freitag, Mike Berners-Lee, Kelly Widdicks, Bran Knowles, Gordon S. Blair, Adrian Friday

**Affiliations:** 1 Small World Consulting, Gordon Manley Building, Lancaster Environment Centre, Lancaster University, Lancaster, Lancashire LA1 4YQ, UK; 2 School of Computing and Communications, InfoLab21, Lancaster University, Lancaster, Lancashire LA1 4WA, UK

**Keywords:** ICT, carbon footprint, trends, AI, big data, data science, IoT, blockchain, policy, regulations

## Abstract

In this paper, we critique ICT's current and projected climate impacts. Peer-reviewed studies estimate ICT's current share of global greenhouse gas (GHG) emissions at 1.8%–2.8% of global GHG emissions; adjusting for truncation of supply chain pathways, we find that this share could actually be between 2.1% and 3.9%. For ICT's future emissions, we explore assumptions underlying analysts' projections to understand the reasons for their variability. All analysts agree that ICT emissions will not reduce without major concerted efforts involving broad political and industrial action. We provide three reasons to believe ICT emissions are going to increase barring intervention and find that not all carbon pledges in the ICT sector are ambitious enough to meet climate targets. We explore the underdevelopment of policy mechanisms for enforcing sector-wide compliance, and contend that, without a global carbon constraint, a new regulatory framework is required to keep the ICT sector's footprint aligned with the Paris Agreement.

## The bigger picture

To avoid catastrophic consequences from climate change, all sectors of the global economy, including *Information Communication Technology* (*ICT*), must keep their greenhouse gas (GHG) emissions in line with the Paris Agreement. We examine peer-reviewed estimates of ICT's GHG emissions, which put ICT's share of global GHG emissions at 1.8%–2.8%. We find pronounced differences and much debate concerning the underlying assumptions behind the peer-reviewed studies, which could suggest that global emissions from ICT are as high as 2.1%–3.9%. All study analysts agree that ICT emissions *will not reduce* without major concerted political and industrial efforts, and we provide three reasons for anticipating that ICT emissions are actually going to *increase* without intervention. Our analysis suggests not all ICT carbon pledges are ambitious enough to meet climate targets, and that policy mechanisms for enforcing sector-wide climate target compliance are lacking. Without a global carbon constraint, sector-wide regulations are required to keep ICT's carbon footprint aligned with the Paris Agreement. With a global carbon constraint, ICT would be a greater enabler of productivity and utility, creating opportunity for the sector to be financially successful as a critical part of a global net zero society.

## Teaser

We examine peer-reviewed estimates of ICT's GHG emissions, which put ICT's share of global GHG emissions at 1.8%–2.8%, yet find the actual share could be as high as 2.1%–3.9% when adjusting for truncation of supply chain pathways. We explore the reasons why current estimates vary, provide three reasons why ICT's emissions will likely increase without intervention, and outline the changes required to policy and industry targets to keep ICT's emissions in line with the Paris Agreement.

## Introduction

The Information and Communication Technology (ICT) sector has seen massive and accelerating growth in the last 70 years. ICT is now so significant that there is an increasing awareness of the potential environmental effects of ICT, particularly on climate change. ICT has a growing “carbon footprint” arising from greenhouse gases (GHG) released from all its life cycle stages. This includes embodied emissions (the GHG emissions released from the extraction of raw materials required, the manufacturing process and transport to the business or user), use or operational emissions (from energy use and maintenance) and end-of-life emissions (disposal). Yet estimates of ICT's footprint and whether it is in fact growing in impact, or held stable or even reducing by efficiency gains and Moore's Law, is very much a topic of lively debate. Many increasingly point also to ICT's potential to decarbonize other sectors. It is argued that this “enablement” is a key ingredient in the pathway to carbon neutrality, and in many ways exempts or justifies the footprint of ICT itself.

In this paper we look at accepted estimates of climate change impacts of ICT now and in the future (Estimating the carbon footprint of ICT) and ask critical questions concerning efficiency: whether efficiency gains could reduce emissions in the ICT sector and global economy over time, or whether these are more than offset by possible “rebound effects.” In this context, we take a broad view of rebound effects to include any increases in emissions due to the introduction of ICT or the efficiencies it enables, and include an example of a rebound effect (Jevons Paradox) in our supplemental information (this supplemental information includes an appendix for this paper, which goes into more depth about our literature review method, analysis, and additional information relevant to this work—specifically: the methodology, estimates of ICT emissions, video streaming, narratives (Six common narratives for ICT's role in climate change), truncation error, the European Commissions' investment in ICT, carbon pledges, renewable energy purchases, and Jevons Paradox). In this paper we also explore the importance of emerging trends in ICT (big data, data science, and artificial intelligence [AI]; the Internet of Things [IoT]; and blockchain) that could provide opportunities for environmental sustainability yet threaten global emissions reduction (ICT Trends: Opportunities and threats), as well as suggest important areas of regulation and governance (Current policy developments and governance in ICT).

Given the topic importance, there are surprisingly few studies analyzing the environmental impact of ICT and they are often characterized by a lack of interrogatability, potential for conflict of interest, a limited scope that leaves out growing ICT trends and an underestimation of ICT's carbon footprint as significant proportions of total emissions are omitted. We draw on peer-reviewed journal articles published from 2015 on the topic (Estimating the carbon footprint of ICT), and analyze trends in ICT and their environmental implications (ICT Trends: Opportunities and threats). For this, we include literature on the energy or carbon impacts of ICT, its major components (e.g., data centers, networks), its major application areas (e.g., AI, IoT), and the impact ICT has on energy or carbon consumption in other sectors. We go also beyond the literature: including consultations with the lead authors of the main studies who are included in this review, as well as other experts, to better assess ICT emission estimations and the associated complexities; and drawing upon research by Small World Consulting (SWC) to account for emissions omitted in many assessment methodologies (see our supplemental information for further details on the model used for assessment). For our policy analysis (European policy and ICT), we focus on European Commission documents and websites, supplemented by an analysis of industry pledges (Self-regulation in the ICT industry) drawn from analysis of annual reports, blog posts, and web pages from major ICT companies.

While there are limitations to our study in review scope and the uncertainties of carbon calculations, we are confident we have captured the main debates, and contribute through our focus on GHG emissions. We specifically focus on GHG emissions rather than electricity consumption as the former drives climate change and the latter does not capture important factors surrounding ICT's environmental impact. Through our analysis, we have found broad agreement on the size of ICT's current carbon footprint, yet there are a range of different views with regard to ICT's future role in climate change, both in terms of ICT's own carbon footprint and its effect on the wider economy's emissions—we discuss the arguments and assumptions underpinning these different views and their policy implications. Nevertheless, analysts included in our investigations agree that ICT emissions will not reduce without major concerted efforts involving broad political and industrial action, and we provide three reasons that indicate ICT emissions are actually going to increase without intervention. It is clear from our study that too much reliance is placed on a switch to renewables, and efficiency gains within and beyond the ICT sector, for achieving carbon targets; significant action through a global constraint (e.g., a carbon cap on extraction), and more assessment of ICT's rebounds and governance are required.

### Estimating the carbon footprint of ICT

In this section, we provide a broad overview of the estimates for ICT's carbon footprint before 2015, and an in-depth analysis of three major peer-reviewed studies of ICT's estimated emissions. We identify the key arguments and assumptions underpinning the different estimates, noting the essential points of agreement and crucially the major points for and against growth in ICT sectors emissions into the future.

### ICT's carbon footprint

**Figure 1:**
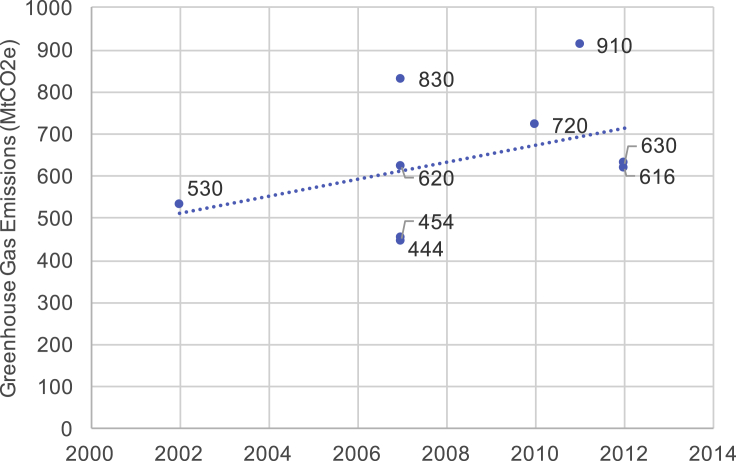
Estimates of ICT's carbon footprint from studies published before 2015 The linear best fit line shows the increase in emissions with time, although the growth is not necessarily linear.

Historically, ICT emissions have grown continuously alongside global emissions. Several studies prior to 2015 have estimated the carbon footprint of ICT (summarized in Figure 1). These show an increase in ICT's carbon footprint over time, even without considering the full life cycle emissions, with the trend line showing a 40% increase 2002–2012. The growth in ICT's emissions has coincided with consistent growth in our total global carbon footprint,^1^ where global GHGs have grown by 1.8% per year^2^ (approximately 20% per decade). This indicates ICT's footprint has likely grown faster than global emissions, with a very uncertain best estimate of twice as fast. Going back in time further, ICT's footprint will have grown faster than global emissions since the sector started from zero mid-last century.

Scientific debate over ICT's emissions has intensified in the last 5 years. We therefore focus on research since 2015—especially studies by three main research groups led by Andrae,^3^, ^4^, ^5^, ^6^ Belkhir,^7^ and Malmodin.^8^^,^^9^ Andrae and Edler^3^ estimate ICT's emissions for every year 2010–2030, Belkhir and Elmeligi^7^ for 2007–2040 and Malmodin and Lundén^8^^,^^9^ for 2015. Malmodin has also provided additional estimates for 2020 to us in personal communication. We summarize the arguments in this section.

#### ICT's current carbon footprint

**Figure 2:**
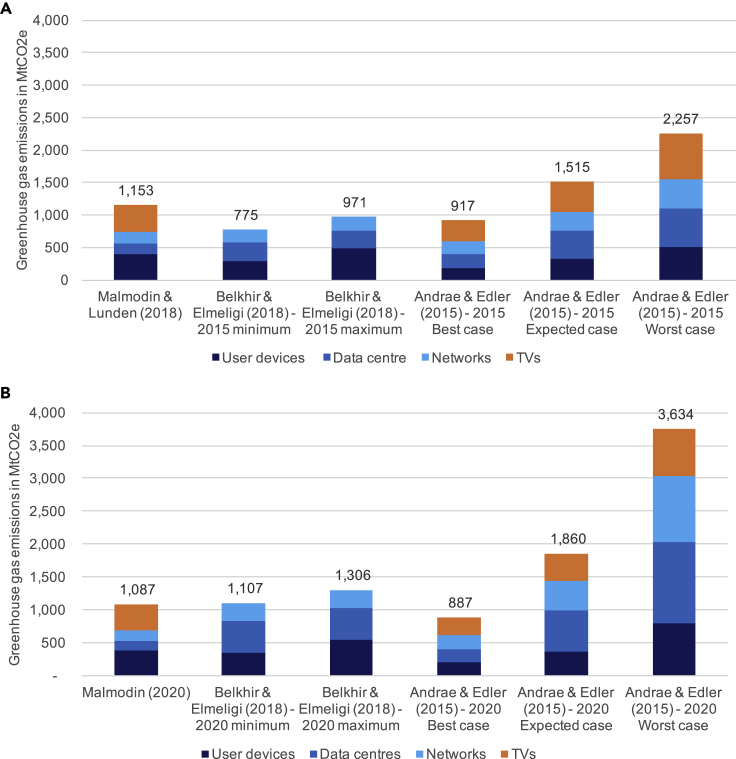
Estimates for global ICT’s carbon footprint in 2015 and 2020 (A) Estimates for global ICT's carbon footprint in 2015. (B) Estimates for global ICT's carbon footprint in 2020. Note that for Malmodin and Lundén's^8^^,^^9^ estimates, TV includes TV networks and other consumer electronics, whereas for Andrae and Edler's^3^ estimates, only TVs themselves and TV peripherals are included. Belkhir and Elmeligi^7^ did not include TVs. Malmodin and Lundén's^8^^,^^9^ original estimates for the ICT and entertainment and media sector includes paper media, which we have excluded here.

ICT is estimated at ca. 1.8%–2.8% of global GHG emissions in 2020. Estimates of ICT's emissions in 2020 (see Figure 2) vary between 0.8 and 2.3 GtCO_2_e. The highest estimates (Andrae and Edler^3^ “worst case”) put ICT's share of global GHG emissions around 6.3%, but Andrae now believes that the Andrae and Edler^3^ “best case” scenario of around 1.5% is more realistic for 2020 (personal communication). Belkhir and Elmeligi^7^ estimates are higher at 1.9%–2.3%, especially considering they omit TVs in their total estimate. Malmodin's estimates sit in between the others at 1.9% of global emissions. When adjusting for differences in scope, these studies point toward a footprint of 1.0–1.7 GtCO_2_e for ICT, TVs, and other consumer electronics in 2020; this is 1.8%–2.8% of global GHG emissions. We stress that this estimate carries some uncertainty but gives us a reasonable idea of the impact of ICT. Across studies, roughly 23% of ICT's total footprint is from embodied emissions, yet the share of embodied emissions for user devices specifically is ca. 50%. This is because, unlike networks and data centers, user devices are only used for parts of the day and use less electricity, but are exchanged often, especially in the case of smartphones. Electricity consumption of user devices and domestic equipment has decreased over the last 15–20 years driven by legislation and public procurement policy, such as the EU ERP directive and EnergyStar (Chris Preist, personal communication). However, efficiency improvements will not reduce embodied emissions drastically. While production processes are becoming more efficient, the manufacturing footprint of smartphones is increasing because of more advanced integrated circuits, displays, and cameras (Malmodin, personal communication). With a large share of their footprint coming from their manufacture, extending smartphones' lifetime is the best way to reduce their footprint. Most studies reviewed here assume an average lifetime of 2 years, partly driven by phone contracts that promise users the newest models.^7^ There are some signs, though, that this might be increasing slightly. For example, the NPD^10^ reported that in the US, the average use has increased to 32 months in 2017 up from 25 months in 2016. Legislation encouraging repair, e.g., the EU Waste Electrical and Electronic Equipment Directive, can help, alongside business models centering around service rather than product provision or selling repairable products to markets in the Global South (Preist, personal communication).

**Figure 3:**
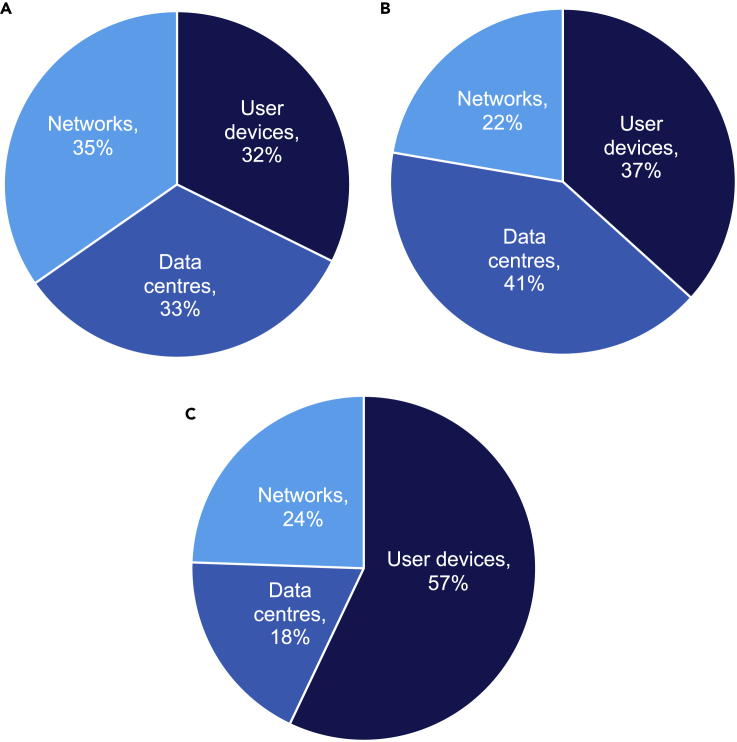
Proportional breakdown of ICT's carbon footprint, excluding TV (A) Andrae and Edler (2015): 2020 best case (total of 623 MtCO_2_e). (B) Belkhir and Elmeligi (2018): 2020 average (total of 1,207 MtCO_2_e). (C). Malmodin (2020): 2020 estimate (total of 690 MtCO_2_e). Andrae and Edler's^3^ best case is displayed because more recent analysis by the lead author suggest that this scenario is most realistic for 2020. Note that Malmodin's estimate of the share of user devices is highest; this is mostly because Malmodin's network and data center estimates are lower than those of the other studies.

There are important differences in how analysts arrived at these estimations. There is a lack of agreement about which technologies ought to be included in calculations of ICT's GHG emissions—particularly TV. All studies include data centers, networks, and user devices as the three main components of ICT, but there are pronounced differences of opinion regarding the proportional impact of each. A comparison of the different proportions in 2020 estimates (excluding TV) is provided below (Figure 3).

Regarding data centers, Belkhir himself noted that his projection of 495 MtCO_2_e for data centers in 2020 is overestimated (personal communication). Recent evidence by Masanet et al.^11^ of 205 TWh total energy use in 2018 seems to converge with Malmodin's estimate of 127 MtCO_2_e in 2020. Assuming a global electricity mix at 0.63 kgCO_2_e/kWh, Masanet et al.’s^11^ estimate comes to ca. 129 MtCO_2_e —higher than Andrae and Edler's^3^ best case estimate of 217 MtCO_2_e.

Studies systematically underestimate the carbon footprint of ICT due to the “truncation error.” This error arises from the partial exclusion of supply chain pathways by the traditional process of life cycle analysis (LCA). Malmodin's studies are the most comprehensive as they include operator activities and overheads (e.g., offices and vehicles used by data center and network operators), as well as considering life cycle emissions of equipment (i.e., from production, use, to disposal) rather than just production energy^3^ or only material extraction and manufacturing energy.^7^ However, Andrae and Edler,^3^ Belkhir and Elmeligi,^7^ and Malmodin and Lundén^8^^,^^9^ all follow LCA methodologies, which are unable to include the infinite number of supply chain pathways of a product, thereby incurring “truncation error” in their carbon accounting. Similarly, but of less significance, they also do not consider the full supply chain carbon footprint of electricity used to run ICT equipment. However, in the assessment of emissions from products, including electricity, the system boundary can be expanded to include all supply chain pathways by combining traditional LCA with environmentally extended input output (EEIO) methodologies. By mapping the LCA's system boundary onto the EEIO model, an EEIO-based estimate can be made of the truncated supply chain pathways. When truncation error has been adjusted for in this way, the carbon footprint for ICT, including TVs and other consumer electronics, rises to 1.2–2.2 GtCO_2_e (2.1%–3.9% of global GHG emissions) in 2020 with ca. 30% coming from embodied emissions and 70% from use phase emissions. We stress once more that these are rough estimates with a significant degree of uncertainty.

#### ICT's future carbon footprint

##### There is broad agreement by analysts in the field on certain key assumptions:

• the world's carbon footprint needs to decrease to avoid climate catastrophe
• data traffic is continuing to grow
• energy demand by ICT is increasing
• demand for data centers and network services will increase
• the shift to smartphones is decreasing emissions from PCs and TVs
• using more renewable energy would reduce ICT emissions
• ICT could reduce emissions in other sectors but not by default and only under certain conditions (contrasting to GeSI^12^ SMARTer, 2030 claims)
• ICT has the potential to increase its own emissions and facilitate rising emissions in other sectors

Opinions are more divided regarding future trends in emissions. From 2015 to 2020, Belkhir and Elmeligi's^7^ and Andrae and Edler's^3^ estimates of ICT emissions have increased due to an increase in data traffic and the number of user devices (see Figure 2). In contrast, Malmodin's estimates have decreased slightly—mostly for data centers (by 10%) due to an increased adoption of renewable energy, and for networks (by 8%) due to decreases in overheads, despite increases in their electricity consumption.

**Figure 4:**
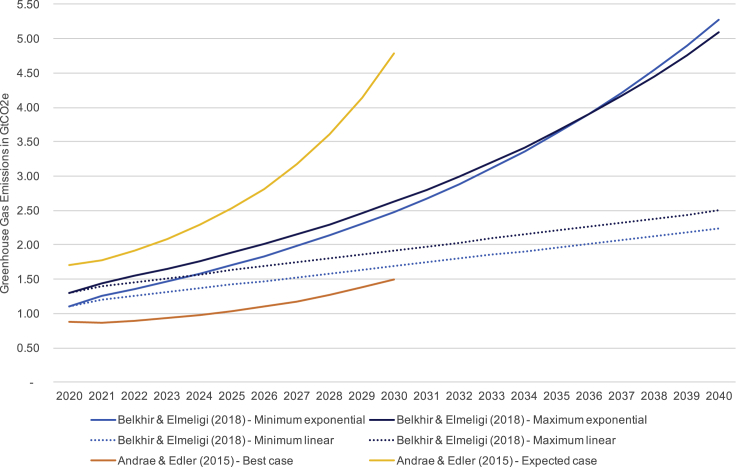
Projections of ICT's GHG emissions from 2020 (A) Andrae, (B) Belkhir, (C) Malmodin, personal communication. Belkhir and Elmeligi^7^ judge their exponential scenario as most realistic, while the linear growth scenario is more conservative and reflects the impact of mitigating actions between now and 2040. Malmodin and Lundén^8^^,^^9^ did not make concrete estimates beyond 2020, but Malmodin suggests that ICT's carbon footprint in 2020 could halve by 2030—offering a 2030 estimate of 365 MtCO_2_e in a recent techUK talk.^14^

Malmodin (personal communication) argues that: GHG emissions from ICT have stabilized for now; ICT and the entertainment and media sector growth is starting to decouple from GHG emissions; and that ICT could even halve its 2020 emissions by 2030 through renewable energy transformation and collective effort^13^ to 365 MtCO_2_e in 2030.^14^ In contrast, Belkhir and Elmeligi^7^ and Andre and Edler^3^ believe that emissions from ICT will continue to grow (see Figure 4).

All analysts think that it would be possible in theory for ICT to decrease its emissions with broad political and industry action—but Malmodin is more optimistic that this will happen than Belkhir and Elmeligi^7^ and Andrae and Edler.^3^ A recent report by Ericsson^15^ based on Malmodin and Lundén^8^^,^^9^ claims that ICT's emissions could be reduced by 80% if all its electricity came from renewable sources.

Differences in predictions could be due to age of data used. The data underlying Andrae and Edler's^3^ and Belkhir and Elmeligi's^7^ work is somewhat older (Andrae and Edler^3^ use some data from 2011 for data centers and networks, while Belkhir and Elmeligi^7^ use data from 2008 for data centers and from 2008 to 2012 for networks) considering ICT's fast pace of development, meaning their projections are potentially based on historical trends that might no longer apply, such as the assumed exponential growth of energy consumption by data centers and networks. In contrast, Malmodin and Lundén^8^^,^^9^ might better capture recent changes in emission trends given their estimates are based on data measured directly from industry (Malmodin and Lundén's^8^^,^^9^ estimates are based on 2015 data; Malmodin's more recent estimates provided in personal communication are based on data from 2018 onward). Malmodin and Lundén^8^^,^^9^ also have the most inclusive scope in terms of ICT equipment, life cycle stages and supply chain emissions considered.

However, this access to industry data inevitably comes at the price of a lack of data interrogatability. Part of Malmodin's data were obtained by ICT companies under confidentiality agreements, preventing others from reviewing the original data and the model's assumptions and calculations. There are also potential risks of conflicts of interest as both authors work for network operators (Malmodin works for Ericsson, Lundén works for Talia). This arguably makes the Malmodin and Lundén^8^^,^^9^ paper open to concerns that claims are less reliable due to selective reporting and assumptions that cannot be properly assessed. We are not suggesting that they cannot be trusted, but the lack of transparency makes independent data and analysis difficult, and transparency is necessary for important policy decisions. As employees of Huawei, Andrae and Edler^3^ also have potential for conflict of interest, but their study is transparent about their data sources, calculations, and assumptions. Belkhir and Elmeligi^7^ have no obvious conflict of interest and they use only peer-reviewed and publicly available sources.

Due to the trade-off between data interrogatability and up-to-date data, it is impossible to judge which study makes the most reliable predictions about ICT's future emissions based on methodology alone. It is possible, however, to examine their arguments and the underlying assumptions to assess which projection is more likely.

### ICT's future carbon footprint: unpacking the studies' assumptions

In the key studies reviewed here, there is disagreement on whether or not:

• energy efficiencies in ICT are continuing
• energy efficiencies in ICT are reducing ICT's carbon footprint
• ICT's carbon footprint will stabilize due to saturation in ICT
• data traffic is independent of ICT emissions
• ICT will enable emissions savings in other industries
• renewable energy will decarbonize ICT

These assumptions have a critical influence on what we can conclude about ICT's role in climate change. We therefore explore the arguments on both sides of the debate to shed some light on the most likely path of ICT's future emissions. In doing so, we draw on several other much-cited sources and direct consultation with key experts.

#### Are energy efficiency improvements in ICT continuing?

There has been a long history of ICT equipment becoming more efficient (and thus cheaper and more productive) with time. Moore's Law allowed the ICT industry to exponentially increase chips' performance, speed, and reduce their power consumption. The exponential improvements of processors has kept the exponential growth in demand partly in check in terms of energy consumption.

While Malmodin and Lundén^8^^,^^9^ acknowledge that Moore's Law has slowed down since 2012, they note that there is usually a time lag before the effects are felt outside of research labs—therefore arguing that efficiencies are continuing for now. Masanet et al.^11^ argue that there is scope for further efficiency improvements in data centers through: improvements in server virtualization; efficiency gains in servers, storage devices, and data center cooling technology; and the move toward large data centers that are more energy efficient due to efficiencies of scale and the ability to invest in AI to optimize energy use.

For efficiency improvements in user devices, there is evidence of carbon savings from TVs: older, more energy-intensive CRT and plasma TVs have been replaced by more efficient LED TVs; and TV sales have dropped due to users now watching video on laptops and smartphones (Belkhir and Elmeligi,^7^ Malmodin). However, smart TVs could change this trend if they become a popular way to access streamed media (Preist, personal communication).

However, efficiency improvements might be coming to an end—a view echoed by some of the experts we have consulted (e.g., Peter Garraghan, Belkhir, Andrae). As transistors have shrunk in size and increased in speed, they have begun to heat up; this led to manufacturers putting a speed limit on processing in 2004. The problem now is “quantum entanglement” where transistor layers become so thin that electrons jump between them, making transistors increasingly unreliable.^16^ Other avenues may exist for improving efficiencies (e.g., decreasing semiconductor use stage power and nanophotonics),^17^ but possibly not on the same timescales^18^ or with the same efficiency gains.

If processor efficiencies are reaching a limit, data centers' power consumption will likely rise as increasing demand will no longer be counterbalanced by increasing efficiency. Despite some remaining scope for further efficiency improvements, Masanet et al.^11^ note that there are limits to efficiency improvements and that energy demand will not stabilize by itself—arguing that urgent policy action and investment are needed to limit increases in energy use driven by increasing demand. Furthermore, efficiencies in ICT do not always guarantee replacement of the older, less efficient equipment (e.g., the development of 5G networks while 2G, 3G, and 4G networks still exist) and new devices or user habits may conflict with replacement gains. For example, some new ICT devices, such as smart watches and smart speakers, are used by people in addition to smartphones and laptops, and Court and Sorrell^19^ also highlight the issue of incomplete substitution of e-materialization trends like e-news or e-books. Multiple user devices in the home have also led to a third of UK households watching separate video content simultaneously in the same room once a week^20^ where people may have watched content using the same TV before.

#### Are energy efficiencies in ICT reducing ICT's carbon footprint?

Malmodin argues that so far, efficiency improvements are continuing, and data center emissions are expected to stay at 1% of global electricity and at the same level of emissions as in 2015 in the next 5 years. Furthermore, Masanet et al.^11^ reported that data centers' operational energy consumption has increased only marginally from 194 TWh in 2010 to 205 TWh in 2020 despite global data center compute instances increasing by 550% over the same time period—showing the effectiveness of efficiency improvements in ICT. Masanet et al.^11^ also note that these efficiency improvements would be able to offset a doubling of data center demand relative to 2018; beyond that point, energy demand will rise rapidly. This is in line with what Belkhir (personal communication) believes, although he is less optimistic about the remaining scope for efficiency improvements.

As highlighted above, ICT has seen rapid and continuous efficiency gains. Yet increases in demand for computation and the number of ICT-enabled devices per person have outpaced these energy efficiency improvements, resulting in growth in ICT's energy consumption and carbon footprint year-on-year. This pattern fits with the rebound effect described by Jevons Paradox whereby an efficiency improvement leads to an even greater proportionate increase in total demand, meaning total resource requirements rise rather than falls, as is often assumed. While Jevons Paradox has not been proved to apply within the ICT industry, it is risky to assume it does not apply given historical evidence of ICT emissions consistently rising despite significant improvements in efficiency (ICT's carbon footprint).

It would be surprising if rebound effects in ICT—and Jevons Paradox in particular—were to end in the future without a foundational change.^21^ There is a theoretical alternative scenario (the reverse of Jevons Paradox) where stalled energy efficiency growth leads to a plateau in ICT emissions due to prohibitive costs as increasing demand cannot be counterbalanced by efficiency improvements any longer. There is little precedent for this in prior work.

#### Are ICT's emissions likely to stabilize due to saturation?

The studies reviewed here all agree that the number of smartphones is increasing. According to Cisco,^22^ there will be 5.7 billion mobile subscribers by 2023–71% of the world population. However, within a few years, every person on earth might have a smartphone and the total number might not further increase (Malmodin, personal communication). There is some evidence suggesting that the average lifetime of smartphones is increasing too,^10^ which will decrease the yearly embodied carbon associated with people replacing their smartphones. In addition, Malmodin argues that there is a limited time per day that people can be using their phones, theoretically capping energy consumption. The same pattern of saturation could be true for other ICT equipment, which could stabilize ICT's emissions.

However, ICT companies generally have a strong incentive to prevent saturation from happening as this would cut their income growth. There is economic pressure for them to create new technologies for individuals and organizations to buy. An example of this is the increase in IoT devices, which require little person time and can operate in the background, driving both embodied and use phase emissions from the production of billions of IoT devices, the networks allowing them to communicate and from data centers that analyze the IoT data (see The Internet of Things). Other important trends (ICT Trends: Opportunities and threats), such as the growth in AI, would also escape this natural saturation. The history of ICT does not provide precedents for a saturation effect; it is therefore unlikely to occur without active intervention. Furthermore, there is still scope for more ICT infrastructure growth beyond smartphones before this innovation cycle even begins, e.g., for data centers in the Global South (Preist, personal communication).

#### Is data traffic independent of ICT emissions?

The amount of data traffic on the internet at a given time does not correspond with simultaneous increases in ICT's emissions. Instead, network operators plan capacity for peak data traffic,^23^ meaning emissions from ICT are fixed regardless of the amount of data traffic until growth in peak capacity is required. In Malmodin and Lundén's^8^^,^^9^ view, data traffic is not directly proportional to emissions due to efficiency gains and use of renewable energy in data centers and networks that allow them to process increasingly more data with similar emissions. Malmodin and Lundén^8^^,^^9^ (reiterated by Ericsson)^15^ believe the energy consumption of ICT is instead linked to the number of users and time spent using ICT because of the energy consumption of user devices and access equipment, such as modems and routers, and that data traffic growth is slowing down to a more linear than exponential growth.

Andrae and Edler^3^ and Belkhir and Elmeligi^7^ both agree that data traffic is a driver in ICT growth and emissions. Growth in the internet's infrastructure capacity allows for new data-intensive services and applications; these offer more affordances to users, driving demand for the services and therefore further infrastructure growth.^24^ Peak data traffic is one driver for this infrastructure growth due to increased demand for data-intensive services; other influences include ensuring technology is always accessible to all users (Preist, personal communication).

Video streaming is a particularly prominent driver in data traffic. During the COVID-19 pandemic, Netflix agreed with EU regulators to reduce their traffic and ease the load on the network, allowing network provision for homeworkers.^25^ Belkhir (personal communication) pointed out that this agreement between Netflix and EU regulators makes it difficult to argue that data traffic is independent of ICT infrastructure growth and therefore that data traffic has little effect on emissions.

#### Is ICT enabling carbon savings in other industries?

In their report SMARTer 2030, the Global eSustainability Initiative,^12^ which represents ICT companies, claim that ICT could save 9.1 GtCO_2_e in 2020 and 12.08 GtCO_2_e in 2030 in other industries, such as health, education, buildings, agriculture, transport, and manufacturing—mostly due to improved efficiency. This would allow a 20% reduction of global CO_2_e emissions by 2030, holding emissions at 2015 levels and decoupling economic growth from emissions growth. Relative to their estimate of ICTs own emissions of 1.27 GtCO_2_e in 2020 and 1.25 GtCO_2_e in 2030, GeSI^12^ argue that ICT is net carbon negative and that governments and businesses should invest more into ICT. According to them, already in 2015, ICT saved 1.5 times its own emissions. There is also a strong argument that ICT will accelerate the use of renewable energy in the grid and hence lead to decarbonization of the energy supply.

The GeSI^12^ report is sponsored by several large ICT companies and there is a lack of transparency in their analysis, raising concerns over possible conflict of interest. So far, there is little evidence that these predictions have come true. History has shown us that growth in the global economy and its carbon footprint has continuously risen, even with ICT creating efficiencies in other industries. It is risky to assume that further ICT-enabled efficiencies will suddenly start to create significant carbon savings in the wider economy without governance and intervention. Rather, it is more likely that ICT enables emission increases in other sectors because it enables efficiencies, leading to growth in the very areas into which ICT delivers those efficiency gains—including growth in industries that are already carbon-intensive (Preist, personal communication). By efficiencies here, it is important to note that we go beyond just energy-specific efficiencies as described by Jevons Paradox; rather, we take into account ICT's emission impacts and rebound effects more widely^cf.^^26^ and refer to any potential route for rebound ICT brings to our society (e.g., consider how ICT has made it far easier to book flights online, contributing to the growth of the aviation industry).

While GeSI^12^ mention rebound effects, this is only in the appendix and given very limited treatment. Their estimate of an increase of global emissions by 1.37 GtCO_2_e due to rebound effects is not included in overall calculations for emission savings by ICT and is almost certainly a serious underestimation. This is highlighted by their example of video conferencing^12^ estimating that “E-Work technologies like videoconferencing could save around 3 billion liters of fuel.” by cutting workers' commutes. It is difficult to quantify the exact balance of ICT-enabled savings and increased emissions, but one clue is that while video traffic has been expanding rapidly to the extent that it is one of the main contributors of internet traffic,^22^ emissions from flights were simultaneously increasing (save for pandemics).^27^ Therefore, ICT only enables efficiencies in other industries if it completely substitutes more traditional carbon-intensive activities rather than being offered in addition to them.

#### Will renewable energy decarbonize ICT?

While the exact share of renewable energy used for the ICT sector is not known, some ICT operators generate renewable energy on-site and the ICT sector overall is a major purchaser of renewable energy—leading the way for a global shift to this energy source. In a recent Ericsson blogpost building on Malmodin's work, Lövehagen^28^ claims that ICT's carbon footprint could be reduced up to 80% if all electricity came from renewable energy. Renewable energy has a much lower carbon footprint than fossil fuel energy at ca. 0.1 kgCO_2_e/kWh. Compared to 0.63 kgCO_2_e/kWh for the global electricity mix, a switch to 100% renewable energy would reduce emissions by ca. 86%. Both of these kgCO_2_e/kWh figures are based on SWC's EEIO model that draws on official data from the UK government's Department for Business, Energy and Industrial Strategy.

With unlimited growth in energy demand, even the relatively small carbon footprint from renewable energy compared to fossil fuel would add up significantly. In addition, there might be limits to the amount of renewable energy that can be generated with present technology, such as the availability of silver, which is used in photovoltaic panels. An average solar panel requires ca. 20 g of silver^29^ and there are currently 2.6 billion solar panels in the world generating a total of 865 TWh.^30^ From 2019 to 2020, 135 TWh of solar energy was added; the manufacture of these requires 52,000 tons of silver. Worldwide, 27,540 tons of silver were being mined in 2020, and the amount increases by ca. 2% every year.^30^ On this trajectory, solar panels would use 100% of global silver supplies in 2031 leaving none for electric car batteries and other uses.

While investments into renewable energy currently have the effect to reduce the price of renewable energy for other sectors, as soon as there are limits to the amount of renewable energy that can be generated, any additional energy used by ICT will take energy away from other purposes. There are also practical constraints on the extent that renewable energy can be used to power ICT equipment. Even data centers that are powered by 100% renewable energy usually have fossil fuel-powered backups for unexpected demand increases. Powering networks with renewable energy is a lot harder due to their decentralized nature,^7^ and powering user devices depends largely on the greening of national grids—a trend that is ongoing in the UK but still far from complete. Thus, while a shift to more renewable energy is crucial, it does not provide an unlimited supply of energy for ICT to expand into without consequences.

#### Six common narratives for ICT's role in climate change

**Figure 5:**
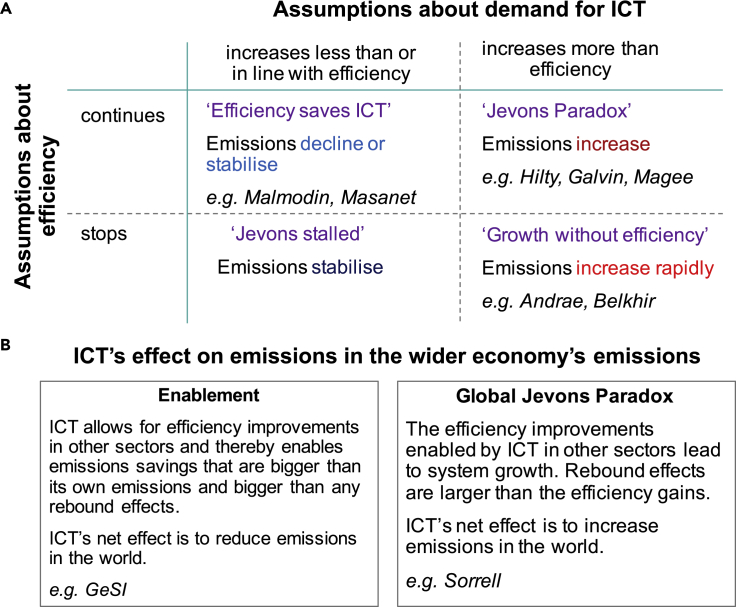
Narratives of ICT's role in climate change and the critical assumptions underlying these (A) ICT's carbon footprint. (B) ICT's effects on emissions in the wider economy. The proponents of each narrative are in italics. Efficiency is here defined as GHG emissions per equivalent ICT use. This includes Moore's Law but also higher renewable energy use, energy efficiency of the infrastructure, etc.

The assumptions from the studies and unpacked in this section can be summarized into six narratives of ICT's future role in climate change (see Figure 5): four around future trends in efficiency and demand and their effect on ICT's own emissions, and two on ICT's effect on emissions in the wider economy.

### Summary of ICT's carbon footprint

**Figure 6:**
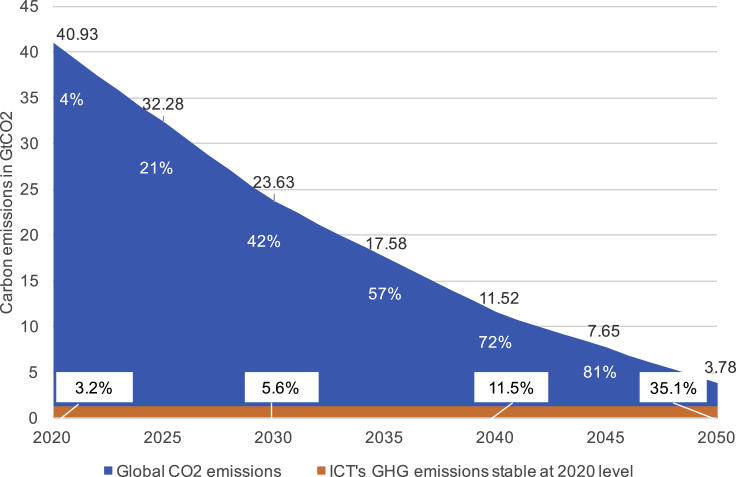
ICT emissions, assuming the 2020 level (adjusted for truncation error) remains stable until 2050, and global CO_2_ emissions reduced in line with 1.5^∘^C under scenario SSP2-19 Numbers on the blue slope indicate global CO_2_ cuts needed relative to 2010 and labels at the bottom indicate ICT's share of global CO_2_ emissions in percent. We assume most of ICT's emissions are from CO_2_ because a large proportion of its footprint is from electricity consumption and there are no agricultural components. The comparison to CO_2_ emissions was chosen because reliable budgets do not exist for GHG emissions at this point.

To meet climate change targets, the ICT sector needs to drastically decrease its own emissions and deliver vast savings in other sectors. Despite some variability in estimates, research studies reviewed here agree that ICT is responsible for several percent of global GHG emissions and that its footprint has grown until recently. The world needs to reduce its GHG emissions to stay within 1.5^∘^C warming.^31^ If the ICT sector should decrease its emissions in line with other parts of the economy, it would have to: reduce its CO_2_ emissions by 42% by 2030, 72% by 2040, and 91% by 2050 (see Figure 6) and net zero by 2050;^32^ or deliver equivalent savings in other sectors in addition to the savings these sectors will have to deliver themselves to meet these targets, making sure that rebound effects do not offset these savings. Global CO_2_ emission cuts to 2050 needed to stay within 1.5^∘^C warming by 2100 are based on modeling by Baskerville-Muscutt^33^ based on the Shared Socio-Economic Pathway 2 as outlined by the International Institute of Applied Systems Analysis;^34^ this is the “middle of the road” or average scenario for the trajectory the world will follow, and cuts are relative to global CO_2_ in 2010. Note that this is CO_2_ only, assuming ICT emissions are mostly CO_2_ as a large part if electricity and there are no agricultural components. The comparison to CO_2_ emissions was chosen because reliable budgets do not exist for GHG emissions at this point.

Under business as usual, increases in emissions are likely. Major concerted effort would be needed to reduce emissions. All the analysts we spoke to agree that to decrease ICT's emissions—even assuming emissions have stabilized—a strong and unified effort would be needed (Current policy developments and governance in ICT). Without this effort, even if ICT's emissions were to stay stable at the 2020 level over the next decades, the relative share of ICT's emissions in global emissions would increase to more than a third as other sectors reduce their emissions in line with 1.5°C warming (see Figure 6).

There are three reasons to believe that ICT's emissions are higher than estimated and that they are going to increase. *Reason 1: rebound effects have occurred since the beginning of ICT*, *and they will likely continue without intervention.* Even if efficiency improvements are continuing (see Are energy efficiency improvements in ICT continuing?), this will not completely counterbalance growth in demand for ICT; in fact, efficiency gains might spur further growth in emissions by allowing the ICT sector to grow further due to rebound effects (see Are energy efficiencies in ICT reducing ICT's carbon footprint?). We believe that a natural peak in ICT emissions due to saturation of demand is unlikely (see Are ICT's emissions likely to stabilize due to saturation?). To the extent that ICT enables efficiency gains in other sectors, there is the risk that rebound effects more than offset any savings following Global Rebounds (see Is ICT enabling carbon savings in other industries?). Renewable energy will help decarbonize ICT but is not a silver bullet (see Will renewable energy decarbonize ICT?).

*Reason 2: current studies of ICT's carbon footprint make several important omissions surrounding the growth trends in ICT.* The studies reviewed here make several important omissions in areas of ICT growth, such as blockchain and partial consideration of IoT. This leads to an incomplete picture. Some analysts argue that blockchain is not part of ICT because it requires specific hardware, not regular servers. However, we believe that it should be in scope of ICT as it is an ICT-facilitated algorithm (see Blockchain); having specific hardware for blockchain is similar to how graphics-intensive services (e.g., online games) require graphics processing units (Preist, personal communication). Malmodin and Lundén^8^^,^^9^ include some IoT and concluded that the impact of IoT is small. However, this is a small share of all IoT and they only accounted for the connected devices, not the energy consumption that IoT creates in data centers and networks (based on the assumption that data traffic and energy are not closely related, see Is data traffic independent of ICT emissions?). Such trends, as well as AI, could help reduce global carbon emissions, but they will also add to ICT's carbon footprint; we discuss this trade-off for prominent ICT trends in the next section (see ICT Trends: Opportunities and threats).

*Reason 3: there is significant investment in developing and increasing uptake of blockchain*, *IoT and AI.* Despite questionable evidence that ICT growth trends will save more carbon emissions than it will introduce (see ICT Trends: Opportunities and threats), blockchain, IoT, and AI are seeing increased investment and uptake. As we explore in Current policy developments and governance in ICT', the European Commission discuss these trends as a way to spur economic growth and yield emission reductions; yet, they expect ICT will only enable 15% reductions, which is insufficient for meeting climate change targets (see European policy and ICT). Some large technology corporations are setting their own carbon pledges, which might help reduce the emissions from ICT's growth trends; however, these pledges are often not ambitious enough to meet net zero emissions by 2050 (see Self-regulation in the ICT industry). Until ICT corporations become net zero, any investment in the ICT industry will be associated with an increase in emissions.

With a global carbon constraint, ICT will be a vital sector to ensure transition to a net zero world. If a global carbon constraint was introduced, we could be certain that rebound effects would not occur, meaning that productivity improvements through ICT-enabled efficiencies both within the ICT sector and the wider economy would be realized without a carbon cost. Under these conditions, ICT would be a key means by which productivity is maintained or increased despite the carbon constraint, and therefore ICT's role in enabling the whole economy can be expected to be even greater than it is today. Given these reasons, under a carbon constraint, ICT's share of global emissions could justifiably be allowed to rise.

### ICT trends: Opportunities and threats

Three recent and emerging innovations may have profound implications for the carbon footprint of the ICT sector: (1) big data, data science, and AI; (2) the IoT; and (3) blockchain and cryptocurrencies. In this section, we explore the opportunities and threats for each, as well as the potential mitigation of such threats.

### Big data, data science, and AI

Big data is one of the most significant technology trends, made possible by the vast data and computational capabilities of cloud computing. Arguments have been made for both the opportunities of realizing a “smart” future and potential growth in ICT's carbon footprint.

#### Opportunities

Big data, data science, and AI could contribute to a lower carbon smart future. Big data/data science/AI and IoT can help bring about a smart and sustainable future encompassing smart grids, cities, logistics, agriculture, homes, etc.^35^, ^36^, ^37^, ^38^ For example, by finding optimal routes through cities and reducing traffic congestion, or by optimizing energy use for building heating and lighting. As these areas rely on IoT, we defer discussion on these opportunities until Internet of Things.

There is a willingness across industry and academia to apply such technologies for the benefits of society. There is a significant move toward data science and/or AI for social good, including applications in health^39^ and the environment, although this work is in its infancy and generally not in everyday practice. The role of big data in supporting green applications has been discussed in the areas of energy efficiency, sustainability, and the environment;^40^ and the field of computational sustainability is emerging, using technologies, such as AI, in support of the United Nations (UN) sustainable development goals.^41^ There is also an emerging research community looking at the role of such technologies in supporting environmental sciences as they seek a deeper understanding of our changing natural environment. See, for example, research in Toronto, Exeter and the Center of Excellence in Environmental Data Science, a joint initiative between Lancaster University and UK Center For Ecology & Hydrology program called “data science for social good.”

#### Threats

The world's data are doubling every 2 years. Data has been described as “the new oil”^42^ given its commercial impact—yet as data storage and data centers grow to meet demand, this description could have a double meaning due to its environmental impacts. Data can help solve complex world problems, but there are concerns over the resources required to facilitate data science and AI, especially the carbon footprint of data centers (see Estimating the carbon footprint of ICT). The total size of the world's digital data was estimated to be 59 zettabytes in 2020, with the amount of data created in the following 3 years expected to be more than the data created in the last 30 years.^43^ AI and data science are therefore an important trend that drives growth in data storage and processing (data processing will be the larger contributor to ICT's energy use, as simply storing data is environmentally cheap in comparison [Preist, personal communication]) and in data centers, which some experts argue leads to an increase in ICT's carbon footprint (Is data traffic independent of ICT emissions?).

Emissions associated with processing this data are increasing due to growing computational complexity. Data science and AI offer additional threats over and above the potential growth of data center emissions. AI has the greatest potential for impact given the complexity of training and inferencing on big data, and especially so-called deep learning. Researchers have estimated that 284,019 kg of CO_2_e are emitted from training just one machine learning algorithm for natural language processing, an impact that is five times the lifetime emissions of a car.^44^ While this figure has been criticized as an extreme example (a more typical case of model training may only produce around 4.5 kg of CO_2_),^45^ the carbon footprint of model training is still recognized as a potential issue in the future given the trends in computation growth for AI:^45^ AI training computations have in fact increased by 300,000× between 2012 and 2018 (an exponential increase doubling every 3.4 months).^46^ Further adding to the threat of AI, ICT companies have been found to use such computationally intensive algorithms for advancing the fossil fuel industry.^47^

#### Threat mitigation

Sustainability needs more consideration in ethical guidelines of AI. Due to this growth of computation, Schwartz et al.^48^ argue the need for “Green AI” that focuses on increasing the efficiency of AI computation rather than the current focus on what they describe as “Red AI,” i.e., accurate AI models trained without consideration of resource costs. Sustainability is currently one of the least represented issues associated with ethics guidelines in AI,^49^ although a framework and “leaderboard” to track the energy consumption and carbon emissions of machine learning has recently been offered in the hope that this will encourage energy efficiency to be considered.^50^ Improvements in efficiency and opportunities may exist, such as addressing the processing requirements of AI algorithms by using idle PCs as a distributed supercomputer.^51^ However, we reiterate the earlier concerns that an efficiency-focused endeavor without a carbon or consumption constraint may fail to mitigate rebound effects (see Are energy efficiencies in ICT reducing ICT's carbon footprint?).

### The IoT

The IoT represent a set of everyday internet-connected objects from wearable technologies through to appliances, cars, and other transport vehicles. This has led to a substantial and ongoing growth of the internet as documented below.

#### Opportunities

IoT technologies can enable efficiency improvements outside of the ICT sector. IoT applications are often viewed as “smart technology,” especially when combined with data science/AI in ways that optimize energy usage more widely. Smart cities aim to provide better public services at a lower environmental cost,^52^ e.g., location-based services from smart city IoT sensing and data analysis can reduce transportation pollution through more efficient driving routes.^53^ Govindan et al.^54^ also investigate how such developments can support smarter logistics, including reducing energy requirements. As mentioned in Will renewable energy decarbonize ICT?, ICT has the potential to decarbonize the energy supply and a combination of IoT and the power grid has real potential to enable the Smart Grid, e.g., by dealing with intermittency of renewable supply.^55^ IoT deployments have been tested in schools with the aim of raising awareness of energy consumption and “promoting sustainable behaviors,”^56^ and IoT has also been harnessed to enable energy efficiency improvements within ICT, e.g., by using IoT to reduce air conditioning for data centers.^57^ These few examples highlight the breadth of IoT opportunities to reduce GHG emissions, as long as the IoT applications *substitute* more carbon-intensive activities rather than act alongside them.

#### Threats

IoT enablement comes at a cost of rapidly rising numbers of devices, device traffic, and associated emissions. The sheer number of IoT devices and the associated data traffic is growing significantly. Innovation in IoT is expected to create a 5-fold increase from 15.41 billion internet-connected devices in 2015 to 75.44 billion in 2025.^58^ Cisco estimate machine-to-machine (M2M) connections will grow from 6.1 billion in 2018 to 14.7 billion by 2023 (a compound annual growth rate [CAGR] of 19%), representing 1.8 M2M connections per member of the global population in 2023.^22^ The majority of these connections is expected to be formed by IoT in the home for automation, security, and surveillance (48% of connections by 2023), yet connected cars (30% CAGR between 2018 and 2023) and cities (26% CAGR) are the fastest growing IoT sectors.^22^

IoT's carbon footprint is under-explored, but will have significant implications for embodied emissions. While the footprint of IoT is uncertain and often unexplored in studies of ICT carbon emissions (Are ICT's emissions likely to stabilize due to saturation?), it has been estimated that the energy footprint of IoT semiconductor manufacturing alone might be 556 TWh in 2016 and increase 18-fold to 722 TWh in 2025.^59^ This does not include other aspects of embodied carbon in IoT, such as material extraction and transport, or sources of GHG emissions other than electricity; it also does not consider energy use of running systems, although Das^59^ estimates that this would be a lot smaller than the embodied carbon in manufacturing, at perhaps 118 TWh in 2016 and decreasing to only 1 TWh in 2025 as we see more energy efficient technologies. This study has also, however, been questioned as being vastly overestimated by Malmodin (personal communication). Assuming a global electricity mix of 0.63 MtCO_2_e/TWh, this would be a total of 424 MtCO_2_e in 2016 and 6,125 MtCO_2_e in 2025 for the manufacture and use of the semiconductors; this is without emissions from the entire IoT device, associated sensors, and the emissions in data centers and networks that IoT communicate with. It is also worth noting that the introduction of IoT could lead to an initial rise in obsolescence for other non-ICT products, as society makes the transition to an IoT-focused life (e.g., replacing a working kettle with an internet-connected kettle).

#### Threat mitigation

Lower energy IoT systems are a way forward, but may lead to energy-intensification and fuel greater emissions overall. Researchers are already looking to create lower energy IoT systems, considering both devices^60^ and communication technologies. One focus is on Low Power Wide Area Networks (LPWANs)^61^ to reduce the energy requirements of M2M communication, but at a trade-off of lower bandwidth. There is an associated field of study referred to as “Green IoT,”^62^, ^63^, ^64^, ^65^ which focuses on ensuring that IoT's own environmental costs are considered as we move toward a smarter society and environment. Yet we should be careful of IoT applications that could lead to rebound effects. For example, smart home technologies have the potential to reduce energy consumption (e.g., through remote-controlled heating or lighting), but could perhaps lead to “energy-intensification” once adopted through offering new services (e.g., pre-heating homes, continuously running security systems) or intensifying current services (e.g., internet connectivity, audio/visual entertainment)^66^—the latter adding to ICT's carbon footprint through additional user devices and data traffic.

### Blockchain

Blockchain is an example of a decentralized algorithm designed to avoid a centralized authority or central point of failure. Blockchain allows for potentially important new uses, e.g., for decentralized financial systems. Cryptocurrencies are the most popular application for blockchain, with Bitcoin being the biggest cryptocurrency available today.

#### Opportunities

Blockchain could offer some opportunities for reducing carbon, but there are no emissions-reducing applications of these technologies yet. A decentralized electronic currency could offer a real disruption in the management of market transactions and in the possibility of handling decentralized energy exchanges,^67^ although there are no real examples of demonstrable emissions savings yet. Kouhizadeh and Sarkis^68^ discuss the potential of blockchain technologies to enhance sustainability in the supply chain, for example, by supporting transparency in the early stages of supply chain management (e.g., vendor selection and evaluation); this work, however, is speculative at this stage, leading to researchers offering directions to further explore adoption of blockchain in this domain.^69^

#### Threats

The energy consumed by single cryptocurrency is equivalent to that of entire nations. Blockchain is underwritten by energy: the algorithm, if based on “proof of work,” creates high levels of replication and redundant computation.^70^ The methodology and assumptions behind Mora et al.’s^70^ projections of blockchain's future energy use have been questioned by Masanet et al.,^11^ but proof of work is widely accepted to be energy-intensive. Energy consumption can also increase through escalation of the “mining arms race” due to improving risk sharing for proof of work blockchains.^71^ Focusing on cryptocurrencies, one study indicates that Bitcoin's annual electricity requirements of 68.7 TWh in 2020 are equivalent to powering 7 million US households,^72^ associated with a footprint of 44 MtCO_2_. This is based on a global average electricity intensity of 0.63 kgCO_2_e/kWh, which is likely an underestimate since the energy used to mine Bitcoin often draws on a higher share of coal than the global average.^73^ Due to the inefficiency of transactions, a single transaction could be ca. 750 kWh, enough to power 23 households for 1 day,^72^ or 473 kgCO_2_e—also based on the (likely underestimated) 0.63 kgCO_2_e/kWh global average electricity intensity. Bitcoin currently has a market dominance of 64% of all cryptocurrencies.^74^ Under the assumption that other cryptocurrencies have the same carbon intensity as Bitcoin, the carbon footprint of all cryptocurrencies would be ca. 69 MtCO_2_e, 0.1% of global emissions. Another study estimated the Bitcoin network electricity consumption at 2.55 gigawatts (GW) in 2018 (a value that is nearly as much as Ireland at 3.1 GW), but that this could rise to 7.67 GW in the future (making it comparable with Austria at 8.2 GW).^75^ Other researchers argue an annual electricity consumption of 48.2 TWh and annual carbon emissions ranging from 23.6 to 28.8 MtCO_2_ for Bitcoin in 2018.^73^ Stoll et al.^73^ also estimated that other cryptocurrencies would add another 70 TWh in 2018, bringing the total carbon footprint to ca. 73 MtCO_2_e in 2018.

#### Threat mitigation

Fiscal policy intervention may be needed to mitigate energy consumption of decentralized algorithms. Alternatives to proof of work exist that could reduce the resources required for blockchain, e.g., proof of stake reduces computation and Byzantine protocols remove consensus mining.^76^^,^^77^ Carbon offset mechanisms for blockchain also exist, such as SolarCoin, whereby solar energy producers are rewarded with a free SolarCoin for each MWh of solar-based electricity they produce.^78^ Renewable energy can also be used to power these technologies and it is argued to form 73% of Bitcoin's mining,^79^ although it is important to note that CoinShares Research who published the report run a cryptocurrency investment fund, so there is a potential conflict of interest. However, de Vries^80^ does not think Bitcoin can be sustainable due to: (1) the seasonality of hydropower in Sichuan, China (a region that supposedly supports nearly half of global mining capacity)^81^ meaning energy is required from alternative sources such as coal; and (2) the e-waste associated with mining machines once they reach their end-of-life (if the cryptocurrency collapses, mining machines cannot be repurposed as a generic data centers since they are so specialized [Preist, personal communication]), estimated at an annual 10,948 metric tons (comparable to Luxembourg at 12 kt) assuming Koomey's efficiencies law.^82^ Despite being the most popular use of blockchain technology, there are, and will continue to be, blockchain applications beyond Bitcoin and cryptocurrencies. To mitigate the energy consumption of blockchain technologies and applications, Truby^83^ has proposed a series of fiscal policy options, such as introducing a customs duty or excise tax on imports of miners' verification devices based on its energy consumption.

### Summary of ICT trends

**Figure 7:**
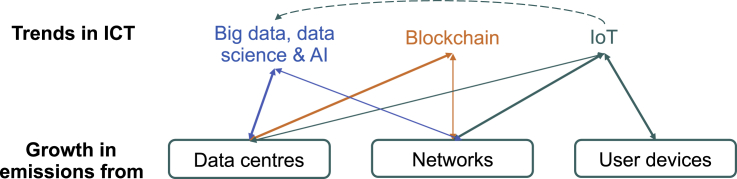
The impacts that trends in ICT have on growth in emissions from data centers, networks, and devices Note that the thicker lines depict prominent threats, thinner lines depict secondary threats, and the dotted lines depict the links between the trends.

If unchecked, ICT trends could drive exponential growth in GHG emissions. The three trends we have discussed could lead to substantial growth in ICT's footprint (see Figure 7 and note that in this section we expand “user devices” to “devices” to include embedded devices). While we have discussed the trends independently, it is important to note that these trends are in fact interlinked. For example, IoT involves collecting more data from sensors, requiring more analytics and adding to the issues raised by big data, data science, and AI, with the potential to further increase ICT's emissions. Such growth trends will also be facilitated through innovations in the ICT infrastructure, e.g., the move from 4G to 5G cellular networks would enable faster, data-intensive network transmissions for IoT devices—allowing for even more data to be collected, communicated, and processed. If not restrained, these above trends all have potential to help drive further exponential growth, unlikely to be outweighed by the ICT-enabled carbon reductions in other sectors.

COVID-19 has shown a consumption constraint that could disrupt these trends. As many activities have been restricted or avoided during the pandemic, ICT has shown the significant benefits and value it can bring to society—allowing families to communicate, people to work from home, and conferences to be held online. Under these circumstances, ICT serves as *a substitution* rather than *an addition* to our regular activities. Coinciding with this, there has been a temporary drop in carbon emissions. A recent study in Nature estimates that daily global CO_2_ emissions temporarily decreased by 17% in early April relative to 2019 levels, largely due to changed transport and consumption levels, and that 2020 annual emissions could decrease by 4% if restrictions remain in place until the end of 2020, and 7% if restrictions end in June relative to 2019.^84^ However, this is negligible if it does not lead to lasting changes after the pandemic. The key question is what society will do when the COVID-19 crisis is over. Will the world embrace some of the new ways of living and working instead of their traditional counterparts and reap the carbon benefits, or return to the old ways, or a mix of the two?

There are important policy decisions to be made that determine the future of ICT's carbon footprint. There is an increasing awareness of the impacts of ICT, but we note the need to expand our awareness to the full range of narratives and their underlying assumptions (see ICT's future carbon footprint: Unpacking the studies' assumptions). We also note that ICT and its trends can bring a lot of value to many people worldwide. Society is very much at a crossroads in terms of the choices faced, and there are some positive signals. For example, in AI research, there have been calls for the EU to incentivize AI applications that are “socially preferable (not merely acceptable) and environmentally friendly (not merely sustainable but favorable to the environment),” recognizing the need for a methodology to assess these characteristics.^85^

Without a global carbon constraint, avoiding unsustainable growth in ICT becomes a debate of what we should prioritize in the ICT sector, what problems can and should be solved using computing, and who can access the required ICT resources for such solutions—supporting valued use of ICT (for example, for uses that lead to carbon reductions in the economy) while constraining consumption and minimizing the ICT sector's carbon footprint. An example of such prioritization in practice is the recent Netflix agreement with EU regulators to reduce its bitrate to ease the burden on the internet during the COVID-19 outbreak, enabling more people to work online from home.^25^ This in turn places the spotlight on policy makers and governance structures at all levels, including in industry, governments, and academia. We look at this important issue in the next section.

## Current policy developments and governance in ICT

Self-governance and the policy landscape is changing. Europe is leading the world in implementation of and experimentation with climate policy,^86^ making the EU Green Deal and the European Commission's (EC) rhetoric particularly worthy of analysis as a bellweather of global climate policy. In this section, we explore such European policy, and also look at self-regulation of ICT emissions by top technology companies to understand whether they are sufficiently ambitious to meet carbon targets without the need of top-down regulation.

### European policy and ICT

ICT is a central pillar of Europe's climate strategy. Under the EC’s Green Deal, Europe is committed to becoming carbon neutral by 2050, and climate neutral later this century.^87^ The EC use the term “carbon neutral” to refer to no net emissions of carbon dioxide, and the term “climate neutral” to refer to no net emissions of GHG emissions. This is different from the way most ICT companies use the term “carbon neutral,” which includes all GHG emissions. ICT features prominently in policymaking around the climate: (1) because of recent efforts to lead the world in a sustainable, human-centric approach to innovation,^88^ and (2) to drive down GHGs across the economy.

European ICT emissions policy emphasizes efficiency, renewables, and circular waste. The EC's official figures put ICT's current share of global GHG emissions at more than 2%,^89^ and a study commissioned by the EC anticipates that “the energy consumption of data centers and telecommunication networks will grow with an alarming rate of 35% and 150% respectively over 9 years” (from 2018).^90^ Rather than seeking to directly affect this consumption trend, policy focuses on mitigating the impacts of rising consumption, specifically through improved efficiency and renewable energy. Three fundamental assumptions are evident in this approach: (1) there is scope for energy efficiency improvements in ICT to continue, at least through 2050 (Are energy efficiency improvements in ICT continuing?); (2) energy efficiency gains in ICT can reduce ICT's carbon footprint (Are energy efficiencies in ICT reducing ICT's carbon footprint?); and (3) renewable energy will decarbonize ICT (Will renewable energy decarbonize ICT?). As we have discussed in Summary of ICT's carbon footprint, there are strong arguments against each of these premises that may impede successful decarbonization of ICT unless simultaneously curbing demand or adding a global carbon constraint. However, publicly facing policy statements do not attend to these counter-assumptions.

Data centers are a particular focus of European policy. The EC has committed to carbon neutral data centers by 2030, through a mixture of continued efficiency improvements, transitioning toward reliance on renewable energy sources, and developing methods of reusing the heat that servers generate.^91^ This is an ambitious proposal, as currently there is no indication that data center emissions are decreasing despite continuous efficiency improvements (see ICT's carbon footprint). The EC also does not specify whether this must be achieved through on-site renewables or can include purchasing of offsets.

Other noteworthy policy covers e-waste, which is recognized by the World Economic Forum as the fastest growing category of waste.^92^ As part of Europe's New Circular Economy Action Plan, the EC plans to put forward a “Circular Electronics Initiative” by the end of 2021 to improve the lifespan, repairability, and recyclability of ICT products.^93^ This initiative would help decrease the embodied carbon of ICT but would be partly offset if the total number of devices continues to increase (i.e., innovation will prohibit saturation in ICT, see Are ICT's emissions likely to stabilize due to saturation?).

Except for this Circular Electronics Initiative, which will likely include a reward scheme for consumers who recycle their old devices,^94^ the Green Deal is notable for its lack of clear incentivization or enforcement mechanisms regarding decarbonization of ICT. It may be believed that efficiency naturally improves as technology advances (e.g., through Moore's Law), and/or that market forces will compel industry to drive these improvements, as there is no discussion of either penalties to be applied or assistance to be offered to the sector toward achieving carbon neutrality by 2050. Also not provided within the Green Deal are estimates of the emissions reductions needed within the ICT sector itself to meet this ambition, which may be incompatible with continuing growth expected of ICT's electricity consumption (see ICT's carbon footprint).

Europe seeks to supercharge enablement through significant investment in ICT. While policies clearly acknowledge ICT's share of global emissions and commit to reducing them, the primary thrust of Europe's climate strategy is the use of ICT to enable emissions savings in other industries (“enablement”). An EC commissioned report states vaguely that ICT “probably saves more energy than it consumes.”^90^ The wording of the Green Deal, however, is unambiguous: “Digital technologies are a critical enabler for attaining the sustainability goals of the Green deal in many different sectors.”^95^ This includes various initiatives and major funding schemes intended to foster innovation in and uptake of AI, IoT, and blockchain.

The Green Deal does not provide a detailed roadmap for how these technologies will in fact deliver against these goals, nor figures regarding expected savings to be achieved. These are undoubtedly difficult to estimate, but as yet there is no evidence in the multi-decade history of ICT-driven efficiency savings that enablement works for reducing overall emissions (see Is ICT enabling carbon savings in other industries?). In the absence of an intervention, such as the introduction of a global carbon constraint, claims of the feasibility of this strategy should be approached with skepticism. As a baseline, staying below 1.5°C warming would require the global economy to reduce by 42% by the year 2030, including the ICT sector (see Summary of ICT's carbon footprint); so if ICT's emissions do not shrink by 42% by 2030, then it would have to enable reductions in other sectors—beyond the 42% that other industries will have to cut anyway—to compensate for this shortfall. This may prove a delicate balancing act. To facilitate this work, complete and accurate estimates of ICT's footprint need to be captured regularly, alongside careful accounting of the emissions ICT is driving or saving in other sectors, with sector targets adjusted accordingly to ensure regional and global targets are met. For this, consistent carbon accounting standards would need to be established across the sector; this would avoid the variability of carbon estimations, as we found with current studies in Estimating the carbon footprint of ICT, from differences in the approaches, boundaries, and data used.

We note the competing policy priorities of the EC. Europe faces pressures to remain competitive in the global technology market and seeks to lead the way in rapidly growing technologies that would otherwise be capitalized by Asian and US competitors.^96^ By stimulating innovation in these areas, Europe seeks to maintain both the health of its economy and the health of the planet. But critically, in the current policy environment, and lacking a global carbon constraint, economic growth would likely further spur consumption and therefore emissions.

### Self-regulation in the ICT industry

Companies need net zero carbon targets that cover supply chain emissions. Several big ICT companies have recently announced carbon pledges to self-regulate their emissions (e.g., Amazon, Apple, BT, Microsoft, Sky). These pledges fall into three main categories: (1) carbon neutral (least ambitious); (2) net zero; and (3) carbon negative (most ambitious). To limit global warming to 1.5°C,^31^ we will need to reach net zero emissions by 2050 globally.^97^ Companies should aim for net zero or, even better, carbon negative. To make this possible carbon neutral targets are not enough because they do not cover supply chain emissions. Yet only a few firmly aim to be net zero (e.g., Microsoft, Sky, Amazon, BT), and only Microsoft aims to be carbon negative.^98^

Carbon offsetting requires truly additional carbon removal methods. Companies need to prioritize reducing the total emissions as much as possible^99^—only then should the rest of their emissions be offset by permanent, verifiable, and additional carbon removal methods. For a company's emissions to be truly offset, the same amount of carbon that the company emits needs *to be removed* from the atmosphere (e.g., through afforestation, reforestation, planting seagrass, taking in landfill gas), not simply avoided. An example of an avoided emission offset is an area of forest that is protected from logging; the amount of carbon that would have been released if the forest was cut down is counted as offset. However, there needs to be some certainty that it would have been removed if it had not been purchased, otherwise these offsets cannot be considered additional. Even genuine “avoided” emissions may end up “leaking” out at another point in the system (e.g., a protected area of forest may just lead to more logging somewhere else in the world).^100^

Only 2% of offsets result in truly additional removals.^101^ Furthermore, some offsetting projects may not be permanent: where forests or peatlands are used to sequester carbon, these carbon stores must be protected from fires or logging—otherwise the carbon removals are negated. Efficiency enablement cannot count as offsetting because it is hard to show that any enabled savings are not negated by rebound effects (see Is ICT enabling carbon savings in other industries?).

Only some renewable energy helps to cut emissions. Some companies also claim, or aim for, power provision from 100% renewable energy without specifying whether they aim to cut emissions. Companies need to detail which type of renewable energy they use (e.g., biofuels, solar, wind, hydro), and what proportion of their renewable energy comes from on-site renewable power generation, Power Purchasing Agreements (PPAs), and Renewable Energy Guarantees of Origin (REGOs), as these differ in their additionality. For a company to claim they are 100% renewable, they should source 100% of their energy through PPAs, on-site renewables, and investment in off-site projects but not unbundled REGOs, because the latter cannot claim additionality. Renewable energy projects should not be considered a removal but rather a scope 2 reduction (see Will renewable energy decarbonize ICT?).

The new ITU standard encourages ICT companies to become net zero by 2050. In collaboration with GSMA, GeSI, and SBTi, the International Telecommunication Union (ITU),^32^ a UN agency focused on the ICT industry, released a new standard in February 2020. The standard aims to reduce ICT's GHG emissions by 45% by 2030, and net zero by 2050, in line with limiting global warming to 1.5°C. The scope of ITU's recommendation includes “mobile networks, fixed networks, data centers, enterprise networks, and end-user devices, but excludes ICT services.” The “voluntary” standard comes with reduction targets for each ICT sub-sector for the next decade. Sub-sectors are defined as per other ITU documentation, specifically clauses A2 to A6 of ITU-TL.1450.^102^ Data center operators adopting the science-based target will need to reduce emissions by at least 53%, mobile network operators by 45% and fixed network operators by 62%.^12^ The targets have been approved by the SBTi and require companies to set targets for scope 1 and 2 emissions and some supply chain scope 3. Most of these reductions between 2020 and 2030 are expected to come from a shift to more renewable and other low-carbon energy sources. The targets are less ambitious than pledges by individual companies, such as BT, Sky, and Microsoft, which commit to reach net zero by 2030 or 2040, but they send a strong signal that the world needs net zero and science-based targets and provide a template that policy makers could adopt.

### Key implications for policy moving forward

The full climate impacts of ICT need to be considered systematically, accounting for end-to-end life cycles and supply chain emissions. It is critical that complete and accurate estimates are used to guide climate policy making and target setting within the sector. Studies of ICT's carbon footprint should strive for interrogatability, but also need to disclose potential conflicts of interest that may affect boundary setting for such calculations. Where technologies are unlikely to be included within the estimates of other sectors' carbon footprints, it is essential that they are included in estimates of ICT's footprint so that climate impacts can be accurately monitored across the economy. It is also vital that calculations do not conflate efficiency improvements with emissions reductions, and that they use methods that allow for objective, high-quality, and up-to-date data and analysis—rectifying the issues of current estimates (see ICT's carbon footprint). This also supports the recommendations by Dobbe and Whittaker^47^ who lobby for carbon transparency, as well as consideration of the full supply chain and rebound effects in carbon accounting.

While ICT offers opportunities to enable reductions in CO_2_ emissions in other sectors, evidence does not support their ability to achieve the significant carbon savings required by 2050. It is important not to overhype ICT's potential to reduce emissions across the economy, thus additional research is sorely needed to provide robust estimates to policy makers. Continued growth in the carbon footprint of the ICT sector cannot be justified on the basis that these technologies may enable sufficient savings in other sectors—particularly as estimations of ICT-enabled emissions savings in other sectors fall short of what is required for meeting agreed targets, and there is a risk that ICT's expansion into other sectors could increase those sectors' emissions (see European policy and ICT). This fundamentally calls into question the presumed role of efficiency within climate strategy. There is clear need to detail sector by sector the savings ICT is expected to produce—reflecting careful balancing of sector footprints within the contexts of regional and global targets—along with developing a detailed roadmap toward delivering on those expectations.

The ICT sector must adopt science-based net zero targets in line with, or better than, the ITU standard; but industry self-regulation may not be sufficient to yield necessary emissions reductions. With growing awareness of the climate emergency, public pressure may be enough to get more ICT companies to announce net zero emissions by 2050. However, there is a lack of net zero pledges thus far. Some companies that have pledged net zero are not on target, or do not have detailed and transparent action plans. Note that this piecemeal approach of individual companies making commitments also comes at a competitive cost for the foreriders, with others gaining financially from being free from such commitments. The way forward for a reduction in ICT's emissions is a sector-wide commitment to net zero that is enforced through incentives and compliance mechanisms, such as procurement clauses that set out carbon criteria and consequences for non-compliance. We flag this as an important issue for the sector but detailed consideration of the form of regulation is beyond the scope of this paper. We also note that an ICT-focused net zero commitment is unlikely to limit the emissions from ICT's impact on the wider economy, unless upstream scope 3 emissions are included in the targets.

There is a pressing need to devise a strategy for constraining consumption of ICT so that efficiency improvements lead to actual emissions reductions and enable productivity to be maintained in a carbon-constrained world. It is likely that unabated growth in demand for ICT will more than offset the emissions saved through improved efficiency of these technologies. The only condition under which these rebound effects would not apply is if a constraint were applied, such as a constraint on consumption or an economic constraint through rising carbon costs (e.g., a carbon tax or a cap on emissions). Policy-enforced carbon caps on global emissions, or carbon pricing for all industries, would help avoid the risk of Global Rebounds; but without a global carbon constraint, policies will be needed to enforce credible and ambitious carbon pledges within the ICT sector (see Self-regulation in the ICT industry). We have outlined below five criteria specifically for ICT sector targets, all of which will need to pervade the ICT sector and be subjected to tough, well-resourced, and independent scrutiny:

1 targets should be inclusive of scope 1, 2, and 3 emissions
2 reduction trajectories should be in line with IPCC recommendations for limiting warming to 1.5°C
3 where transition to renewable energy is part of the decarbonization pathway, a careful test should be applied that the renewables are provably additional
4 emissions offsets need to pass tests of *permanence*, *verifiability*, and *additionality*
5 where “net zero” or “carbon neutral” targets are announced, these should be disaggregated into an emissions reduction component and an offsetting component so that offsets are not allowed to replace reduction responsibilities
6 emission reduction targets should not be replaced by enablement claims due to the risk of rebound effects

Top-down, deliberate direction of ICT research and development may be needed to meet global carbon targets. In a world where consumption of ICT needs to be constrained, “worthy” uses of ICT may need to be weighed against other “less worthy” ones. The ICT sector plays an essential role in helping people live better, and it needs to continue to do so while carefully managing demand. Binding commitments to emissions targets for the ICT sector are needed to force decision making that prioritizes the environment over profit when these are in conflict. Unprecedented coordination across the sector in collaboration with policy makers is required to design and enact a plan for achieving net zero emissions from ICT by 2050.

## Discussion and conclusions

As we have explored in this report, there are two central issues for the ICT industry with respect to the climate emergency: ICT's own carbon footprint; and ICT's carbon impact on the rest of the global economy. There has been surprisingly little research into these questions given their significance in response to climate change. The evidence that does exist needs to be interpreted with awareness of problems arising from the following issues: (1) the age of the data; (2) a lack of data interrogatability; (3) a potential for conflict of interest (especially where researchers are employed by ICT companies, and data and analysis is not freely available); and (4) varying approaches to, and lack of agreement on, the boundaries of the analysis of specifically what constitutes the ICT industry in terms of inclusion in estimates of its carbon footprint (e.g., whether or not growth trends in ICT such as blockchain are included, how scope 3 emissions in the supply chain are included to avoid truncation error).

Historically we can be sure that four phenomena have gone hand in hand: ICT has become dramatically more efficient; ICT's footprint has risen to account for a significant proportion of global emissions; ICT has delivered increasingly wide-ranging efficiency and productivity improvements to the global economy; and global emissions have risen inexorably despite this.

Looking to the future, our concerns are that this growth in emissions will continue at a time when emissions *must shrink*. All analyses reviewed in this report concur that ICT is not on a path to reduce emissions in line with recommendations from climate science *unless additional steps* are taken by the sector, or legislators, to ensure that this happens. Prevalent policy emphasis on efficiency improvements, use of renewables and circular electronics is likely insufficient to reverse ICTs growth in emissions. There are real concerns that the period governed by Moore's Law is coming to an end, and there is huge investment in trends that can significantly increase the carbon footprint of ICT, including in AI, IoT, and blockchain. Recently there are encouraging signs that some ICT giants may be moving in a positive direction (e.g., through net zero and carbon-negative targets that include their supply chains), yet there is a lack of policy mechanisms for enforcing sector-wide climate target compliance. Our hope is that with the right policy to enforce these commitments, ICT companies will be able to deliver on their pledges and that other industries will follow ICT's example, allowing us to stay within 1.5°C warming.

Based on the evidence available, it is also key that regulators move away from the presumption that ICT *saves more emissions than it produces*—at the very least it would seem unsafe to assume that ICT efficiencies bring about carbon savings by default. While ICT offers opportunities to enable reductions in GHG emissions in other sectors, evidence does not support their ability to achieve the sustained significant carbon savings we require by 2050. And while ICT might make lower carbon living possible, this will not in itself help to bring about a cut in carbon, and conceivably may lead to rebound effects leading to higher emissions overall. The argument of enablement simply does not exempt the ICT sector from addressing its own emissions, and the sector could certainly do more to understand its enablement and rebound effects. To ensure current technologies have a truly positive impact on the environment, the climate emergency requires a global constraint such as a carbon cap on extraction, a price on carbon emissions, or a constraint on consumption, to rule out rebounds in emissions. With this in place, the ICT-enabled carbon reductions could be realized, and the ICT industry could become a vital sector for the transition to a net zero world.

## Experimental procedures

### Resource availability

#### Lead contact

Any queries related to our review resources should be directed to Kelly Widdicks (k.v.widdicks@lancaster.ac.uk).

#### Materials availability

No new unique reagents were generated as a result of our review.

#### Data and code availability

The data from our figures is available on Lancaster University's Pure research repository here: https://doi.org/10.17635/lancaster/researchdata/477. Belkhir requested their raw data were kept confidential for Figure 4, so this is not available for the relevant.csv file in the repository. No code was used for the analysis of the data in this review, but we did draw on research by Small World Consulting (SWC) Ltd. into sector emissions to adjust estimates by the key studies in Estimating the carbon footprint of ICT for truncation error; details about this research are provided in the supplemental information (Appendix A.5.4).

## Supplemental information

Document S1. Supplemental Appendices

Document S2. Article plus supplemental information
