# The real climate and transformative impact of ICT: A critique of estimates, trends, and regulations

**DOI:** 10.1016/j.patter.2022.100576

**Published:** 2022-08-12

**Authors:** Charlotte Freitag, Mike Berners-Lee, Kelly Widdicks, Bran Knowles, Gordon S. Blair, Adrian Friday

## Main text

(Patterns *2*, 1–18; September 10, 2021)

In the originally published version of this article, the authors inadvertently cited an incorrect source for the following statement: “…and a study commissioned by the EC anticipates that ‘the energy consumption of data centers and telecommunication networks will grow with an alarming rate of 35% and 150% respectively over 9 years’ (from 2018).^90^’ The correct reference [90] is European Commission (2018). Expert and stakeholder consultation workshop on Research on Green ICT 2020-2030. https://digital-strategy.ec.europa.eu/en/library/expert-and-stakeholder-consultation-workshop-research-green-ict-2020-2030. The original reference [90] should instead be [95] and all subsequent references renumbered accordingly.

These errors have now been corrected online, and the authors apologize for any confusion they may have caused.

