# Supplementary material for: The real climate and transformative impact of ICT: A critique of estimates, trends, and regulations
Source: Patterns (N Y). 2021 Sep 10;2(9):100340. doi: 10.1016/j.patter.2021.100340 (PMC8441580; doi:10.1016/j.patter.2021.100340)
Supplement: Document S1. Supplemental Appendices [file mmc1.pdf]

Patterns, Volume *2*

Supplemental information

The real climate and transformative impact of ICT: A critique of estimates, trends, and regulations

Charlotte Freitag, Mike Berners-Lee, Kelly Widdicks, Bran Knowles, Gordon S. Blair, and Adrian Friday

Supplementary Material: Appendix

A Methodology

A.1 Definitions

Table A.1 *Definitions for terms used throughout the report. Unless a reference is provided, these are pulled or adapted from the Cambridge Dictionary [2020] or Berners-Lee [2011].*

**Term**	**Definition**
1.5°C	1.5 degrees Celsius global warming has far fewer climate-related risks in terms of sea level rise, drought, hot weather and precipitation extremes than 2 degrees Celsius. For this reason, world leaders agreed to limit global warming to well-below 2 degrees Celsius and 'in pursuit' of 1.5 degrees Celsius at the 2015 United Nations Climate Change Conference in Paris [IPCC 2018].
2G/3G/4G/5G	Second, third, fourth and fifth generation communication technology.
Artificial Intelligence (AI)	The study of how to produce machines that have some of the qualities that the human mind has, such as the ability to understand language, recognize pictures, solve problems, and learn.
Algorithm [in the context of Blockchain/AI/Natural Language Processing]	A set of mathematical instructions or rules that, especially if given to a computer, will help to calculate an answer to a problem.
Augmented reality	Images produced by a computer and used together with a view of the real world.
Big data	Very large sets of data that are produced by people using the internet, and that can only be stored, understood, and used with the help of special tools and methods.
Bitcoin	A type of cryptocurrency.
Blockchain	A decentralised algorithm. In the context of cryptocurrencies: a system used to make a digital record of all the occasions a cryptocurrency is bought or sold, and that is constantly growing as more blocks are added.
Cap and trading scheme (for carbon)	A cap is set on the total amount of certain GHGs that can be emitted. Within this cap, companies buy or receive emission allowances, which they can trade with one another. At the end of the year, a company must give up enough allowances that cover all its emissions or face a fine. Any spare allowances can be kept to cover future emissions or sold to other companies.
Carbon	A shorthand for all the different global-warming greenhouse gases.

Carbon footprint	A best estimate for the full climate change impact of something, including all greenhouse gases, expressed in carbon dioxide equivalent (the amount of carbon dioxide that would have the same impact as the specific greenhouse gas associated with a thing); the central climate change metric.
Carbon intensity	The amount of greenhouse gas emissions associated with an activity.
Carbon negative	The process by which an activity sequesters more greenhouse gas emissions than are emitted through said activity.
Carbon neutral	Releasing no net greenhouse gas emissions into the atmosphere. Typically achieved by reducing emissions and using offsets to counterbalance any emissions generated.
Climate change	Changes in the earth's weather, including changes in temperature, wind patterns, and rainfall, especially the increase in the temperature of the earth's atmosphere that is caused by the increase of particular gases, especially carbon dioxide.
Cloud computing	The use of services, computer programs, etc. that are on the internet rather than ones that you buy and put on your computer.
CO_2	Carbon dioxide, the most common greenhouse gas.
CO_2e	Carbon dioxide equivalent. Different greenhouse gases have different global warming potentials. CO_2e expresses the climate change impact of all greenhouse gases emitted in association with an activity as the amount of carbon dioxide that would have the same climate change impact.
Cryptocurrency	A digital currency produced by a public network, rather than any government, that uses cryptography to make sure payments are sent and received safely.
Data centre	A place where a number of computers that contain large amounts of information can be kept safely.
Data science	The use of scientific methods to obtain useful information from computer data, especially large amounts of data.
Data traffic/Internet traffic	The activity of data and messages passing through an online communication system or the number of visits to a particular website.
Decarbonising	Reducing the carbon footprint of an activity.
Dematerialisation	Reducing the amount of material needed to produce a product.
Downstream traffic	Data traffic that is moving in a downstream direction (i.e. being downloaded).
Economy-wide impacts of ICT	The impact the ICT industry has on other industries, for example through allowing for efficiencies, providing additional products and/or replacing more traditional technologies, but also allowing intensified activity or growth in other areas of the economy. The effect can be both to

	increase or decrease impact and those other industries. Differentiated from ICT’s impact within the ICT industry. The net effect of ICT depends on the impact it has in both areas and their balance.
Emissions	A shorthand for greenhouse gas emissions.
Entertainment and Media (E&M) sector	A sector category used by Malmodin and colleagues; it covers TV, consumer electronics (such as cameras and audio systems in a car and portable GPS) and print media.
Environmentally Extended Input Output (EEIO) analysis	A “top-down” approach for estimating life cycle emissions, capable of capturing impacts from the entire supply chain. See Appendix F for details.
Embedded device/system	A computer system that does a particular task inside a machine or larger electrical system, or physical object.
Embodied carbon/emissions	The greenhouse gas emissions released from the extraction of raw materials required, the manufacturing process and transport and distribution of a product. It includes a share of all the activities required to take goods and services at the point of sale, but excludes the product use phase. It can be from cradle to factory gate, from cradle to site of use or from cradle to grave – in the latter case, end of life emissions are included. In this report, we assume cradle to point of sale unless otherwise stated.
Enablement	The avoidance of emissions in the wider economy through ICT applications, including through improved efficiency.
End of life emissions (see lifecycle stages)	Emissions after disposal of a product, after the end of the use phase.
Energy footprint	The amount of energy used by a product, activity or industry.
Exponential growth	A rate of increase which becomes quicker and quicker as the thing that increases becomes larger.
Fossil fuel	Fuels, such as gas, coal, and oil, that were formed underground from plant and animal remains millions of years ago.
GB (Gigabytes)	A unit of computer information consisting of 1,000,000,000 bytes.
Greenhouse gas (GHG) emissions, or emissions for short	Gases that contribute to global warming, including carbon dioxide (CO_2), methane (CH4), nitrous oxide (N2O) and fluorinated gases.
ICT’s own impact	The impact the ICT industry has in terms of its energy use or GHG emissions through the entire lifecycle of its products, including their manufacture, operation and disposal. Differentiated from the effect ICT has on other industries, that is, economy-wide impacts.
Information Communication Technology (ICT)	The use of computers and other electronic equipment and systems to collect, store, use, and send data electronically.
Internet of Things (IoT)	Objects with computing devices in them that are able to connect to each other and exchange data using the internet.

Life Cycle Analysis (LCA)	The detailed study of the series of changes that a product, process, activity, etc. goes through during its existence and the resulting environmental impact.
Lifecycle stages (Material extraction, manufacturing, transport, use phase, end of life)	The stages of resource use and environmental releases associated with an industrial system from the extraction of raw materials from the Earth and the production and distribution, through the use, and reuse, and final disposal of a product.
Machine-to-Machine (M2M) communication	The act of sending data between machines or computers.
Machine Learning (ML)	The process of computers changing the way they carry out tasks by learning from new data, without a human being needing to give instructions in the form of a program.
Moore's Law	The observation by Gordon Moore of Intel Corporation that the cost of a computer chip for a particular amount of processing power will continue to fall by half every two years.
Natural Language Processing (NLP)	A field of Artificial Intelligence that gives the machines the ability to read, understand and derive meaning from human languages [Lopez Yse 2019].
Net zero	Having no net climate change impact through greenhouse emissions in a company's value chain. This is achieved by reducing greenhouse gas emissions in the value chain and removing the remaining emissions through additional carbon removals.
Network	A number of computers that are connected together so that they can share information.
Offset	A mechanism to negative a certain amount of GHG emissions either through avoiding emissions elsewhere (e.g. through protecting a forest from logging) or removing emissions form the atmosphere (for example through natural carbon sequestration, such as peatland restoration or reforestation projects). There is debate whether avoided emissions should count as additional and whether they are permanent.
Operational emissions	See use phase emissions.
Operator activities	Activities by operators of manufacturing plants, data centres and networks, such as office heating and lighting, business travel, maintenance of equipment, a share of which should be allocated to the lifecycle emissions of equipment using data centre and network services.
Proof of Work / Proof of Stake	Types of Blockchain consensus algorithms which are processes in computer science used to achieve agreement on a single data value among distributed systems [CoinBundle Team 2018].
Rebound effect	The way in which micro-actions can be nullified by counter balancing adjustments elsewhere in the global system. Often used to refer to the way increased energy efficiency leads to more energy usage overall.

Renewable energy	Energy that is produced using the sun, wind, etc., or from crops, rather than using fuels such as oil or coal.
Router	A piece of electronic equipment that connects computer networks to each other, and sends information between networks.
Scope 1 emissions	Direct emissions from burning of fossil fuels on site (includes company facilities and vehicles)
Scope 2 emissions	Indirect emissions from purchased electricity and gas.
Scope 3 emissions	All other indirect emissions in a company's value chain; including upstream emissions in the supply chain (e.g. emissions from purchased goods and services, transportation of these goods to the company, use of leased assets such as offices or data centres, business travel and employee commuting) and downstream emissions (from transportation, distribution, use and end of life treatment of sold products, investments and leased assets). Scope 3 emissions form the majority of a company's emissions.
Semiconductor	A material, such as silicon, that allows electricity to move through it more easily when its temperature increases, or an electronic device made from this material.
Server	A central computer from which other computers get information.
Set top box	An electronic device that makes it possible to watch digital broadcasts on ordinary televisions.
Smart technology [e.g. smart grids/cities/ logistics/agriculture]	An object/city/process etc. that is internet-connected and therefore able to make intelligent decisions.
Supply chain emissions	Emissions that occur upstream of a company's own operations, including emissions from purchased goods and services, transportation of these goods to the company, use of leased assets such as offices or data centres, business travel and employee commuting. See upstream scope 3 emissions.
Truncation error	In the context of carbon accounting, truncation error describes the omission of some proportion of the total carbon footprint by LCAs because this approach is unable to track all the supply chain pathways associated with a thing. That means, they disregard or *truncate* the pathways that individually only contribute a small share of the total, often set to less than 1%, even though these can make up sizeable share of the total if they are all added up. See Appendix F for more detail.
Upstream traffic	Data traffic that is moving in an upstream direction (i.e. being uploaded).
Use phase/Operational emissions	Emissions associated with the use of a product, mainly from energy use and maintenance.
Value chain emissions	Emissions occurring in a company's value chain, both upstream in the supply chain (from manufacture and

	transport), from its operations and downstream (from product use by customers).
Video-on-demand services	Services (e.g. Netflix, Amazon Prime, BBC iPlayer) that provide a system for watching films or recorded programmes on the internet or television at any time.
Virtual reality	A set of images and sounds, produced by a computer, that seem to represent a place or a situation that a person can take part in.
Virtualisation/ Server virtualisation	The process of changing something that exists in a real form into a virtual version.

A.2 Abbreviations

Table A.2 Abbreviations used throughout the report.

Abbreviation	Term
AI	Artificial Intelligence
A&E	Andrae and Edler (2015)
BEIS	Department for Business, Energy and Industrial Strategy
B&E	Bekhir and Elmeligi (2018)
CEH	UK Centre for Ecology and Hydrology
CRT	Cathode Ray Tube
DAC	Direct Air Capture
EC	European Commission
EEIO	Environmentally Extended Input Output
GeSI	Global e-Sustainability Initiative
GHG	Greenhouse gas emissions
GSMA	Global System for Mobile Communications Association
ICT	Information and Communication Technology
IoT	Internet of Things
ITU	International Telecommunication Union
LCA	Life Cycle Analysis
LED	Light-Emitting Diode
M&L	Malmodin and Lundén (2018)
PC	Personal Computer
PPA	Power Purchasing Agreements
REGO	Renewable Energy Guarantees of Origin
SBTi	Science Based Targets initiative
SWC	Small World Consulting
TV	Television
UN	United Nations

A.3 Units

Emissions are measured in kilograms (kg), tons (t), kilotons (kt), megatons (Mt) and gigatons (Gt). GHG emissions, for example, are often expressed in $MtCO_2e$ or $GtCO_2e$.

1 Gt = 1,000 Mt; 1 Mt = 1,000 kt; 1 kt = 1,000 t; 1 t = 1,000 kg

Energy consumption is measured in watt-hour (Wh), kilowatt-hour (kWh), megawatt - hour (MWh), gigawatt -hour (GWh) and terawatt -hour (TWh).

1 TWh = 1,000 GWh; 1 GWh = 1,000 MWh; 1 MWh = 1,000 kWh; 1 kWh = 1,000 Wh

A.4 Scope

For the purposes of this report, we have adopted a broad definition of ICT to include all types of data centres, networks and user devices used for processing, storing, sending and receiving digital information. This includes data centres of all scales (i.e. servers run by companies in cupboard up to large data centres), all major types of networks (telephony, mobile and broadband data, TV), and a wide range of digital end user devices, such as PCs, laptops, tablets, mobile and fixed phones, TVs, displays and gaming equipment (see Appendix B.2.1). We included all stages of equipment lifecycle, from the extraction of the raw materials, manufacture, transport and use to end of life. For networks and data centres, we included infrastructure (such as the construction and running of the building housing the servers, including cooling, and the digging down of network cable tracks) and operator activities (e.g. business travel, office heating and lighting etc.).

A.5 Method

For the literature review, we built on our collective knowledge of the literature and carried out additional literature searches using Google Scholar, the ACM Digital Library and the citation information from relevant papers. Note that this was not a systematic literature review. For the main review of ICT's carbon impact (Section 2), we included peer-reviewed journal articles published from 2015 onwards with the key words outlined below. These key words were also drawn upon to facilitate our analysis of the trends in ICT and their environmental implications (Section 3). For our policy analysis (Section 4.1), we focused solely on European Commission documents and websites. Our analysis of industry pledges (Section 4.2) draw on a survey of annual reports, blog posts and web pages for 18 major ICT companies (Microsoft, Sky, Vodafone, Apple, Amazon, Netflix, Facebook, Tesla, Google, Samsung, Ericsson, Spotify, Huawei, Cisco, Sony, Nintendo, Intel and IBM).

A.5.1 Key words

- Sustainability
- Energy consumption/ energy
- Carbon emissions
- GHG (Greenhouse gas) emissions
- LCA (Life-Cycle Analysis)
- Efficiency
- ICT/ IT (information technology)/ digital technologies

- User devices
- Internet traffic/ Internet
- Data traffic
- Data centres/ data centers
- Communication networks/ networks
- Big data
- AI (Artificial Intelligence)
- Machine learning
- Data science
- IoT (Internet of Things)
- Smart (home, grid, city)
- Cryptocurrencies
- Blockchain
- Bitcoin
- Video streaming/ video
- YouTube
- Video-on-demand
- TV/ television
- Cloud computing/ services
- Jevons Paradox
- Rebound effect

A.5.2 Selection of key papers

Articles were selected guided by the following questions:

- Does the paper focus on the energy or carbon impacts of ICT, its major components (e.g. data centres, networks), or its major application areas (e.g. AI, IoT)?
- Does the paper focus on the impact ICT has on energy or carbon consumption in other sectors?

A.5.3 Consultation with key experts

In addition to this, we consulted with the following leading experts based on their extensive knowledge on the carbon impacts of ICT through video conference calls:

- Dr. Lotfi Belkhir (Associate Professor at W Booth School of Engineering Practice and Technology, McMaster University)
- Dr. Anders Andrae (Senior Expert at Huawei Technologies)
- Jens Malmodin (Senior Specialist at Ericsson)
- Dr. Peter Garraghan (Reader in Distributed Systems at the School of Computing and Communications, Lancaster University)
- Livia Cabernard (PhD student at the Institute of Science, Technology and Policy, ETH Zurich)
- Prof. Chris Preist (Professor of Sustainability and Computer Systems at University of Bristol)

We discussed their research in relation to ICT's carbon footprint, their opinion of other prominent studies, their response to criticism from the other experts, their view on the

future of ICT's emissions and on the trends posing risks and opportunities for ICT's impact on climate change.

A.5.4 Other sources of information

For this report, we drew on research by Small World Consulting (SWC) Ltd. into sector emissions to adjust estimates by the key studies in Section 2 for truncation error.

SWC developed an environmentally extended input output (EEIO) model (described in detail by Berners-Lee et al. [2011] and Kennelly et al. [2019]) that uses data from the Office of National Statistics on the expenses and GHG emissions from 105 industries in the UK to calculate the carbon intensity per Pound spent. This allows us to model carbon flows in the UK economy and the upstream scope 3 emissions of an industry in its supply chain, by tracking the economic activity stimulated by each sector in other sectors. In contrast to LCAs, SWC's EEIO model tracks 100% of all supply chains associated with a sector. It can be used to estimate the truncation error of LCAs for a particular sector; that is, the percentage of the total emissions that is typically omitted by an LCA. We note that SWC's EEIO model is based on UK emissions data which are not representative of other economies, yet it provides a good–enough estimate to help understand the potential truncation error incurred by LCA estimates (Appendix F).

For manufacture of ICT equipment, these omissions include radiative forcing, manufacture of buildings and machines, of mining equipment and of transport vehicles and other operator activities and overheads associated with the manufacture of a product. We also know that most LCAs do not include pathways that contribute less than 1% of the total carbon footprint. In total, these excluded pathways make up 40% of the total embodied carbon.

For the operation of ICT equipment, electricity is the most important source of GHG emissions. Based on a hybridised EEIO-LCA model SWC developed from scope 1 and 2 emissions data from BEIS [Department for Business, Energy and Industrial Strategy 2019] and the IEA [2019], SWC estimates that the carbon intensity of global average grid electricity in 2018 was 0.63 kgCO_2e/kWh or MtCO_2e/TWh. The carbon intensity factor for electricity used in most LCAs includes emissions from electricity generation and transmission and distribution losses, but not extraction and transportation of fuel to the plant, the manufacture of equipment used in these processes and operator activities. Based on this, we estimate that LCAs omit 18% of the use phase carbon. Our truncation error mark-up applied to the key studies reviewed here is based on the difference between the specific electricity intensity factor they report and SWC's factor of 0.63 kgCO_2e/kWh.

All percentages out of global GHG emissions are based on a total of 57.9 GtCO_2e in 2020. This is based on 55.6 GtCO_2e GHG emissions, including land use change, in 2018 [Olivier and Peters 2019], assuming the growth rate of 2% in 2018 applies to 2019 and 2020. Note that this extrapolation did not consider the impact of Covid-19 on emissions.

A.6 Limitations

This report is not based on a systematic literature review but rather built on our own knowledge of the sector alongside strategic literature searches aimed at covering the

main studies in the field. We have focused on critically analysing the main arguments surrounding the ICT sector's environmental footprint and trends. We have not scrutinised reports about the impact of individual components of ICT (e.g. the carbon impacts of servers alone) or covered the full breadth of research papers within ICT on IoT, Blockchain and AI that do not take an environmental position (e.g. instead focus solely on health, finance, etc.). We have limited our discussion of impacts on the wider economy to the ICT sector's potential to enable efficiencies or drive emissions in other sectors; a full, economy-wide assessment of ICT's environmental impacts globally was deemed out of scope for this study. We are confident, however, that we have captured the main academic debates and the most relevant non-academic publications on the climate change impact of the ICT sector as a whole and the impacts of prominent ICT trends going forward. We call for future work to fully assess the Enablement and Global Rebounds narratives (see Figure 5) on the world's economy.

Carbon accounting is a rather imprecise science due to the complexity of the supply chain emissions pathways and issues with how to allocate emissions to a particular product, activity or sector. For each carbon footprint calculation, there is a margin of error. The uncertainty increases even further for projections of future emissions, as these are influenced by the actions of companies, policy makers, individual users and unforeseen events like natural catastrophe and pandemics. There are several unknowns including what changes future innovations might bring or the carbon footprint of activities which are largely undocumented (e.g. the dark web). The carbon footprint of some of the emerging ICT trends are also difficult to calculate, e.g. IoT and Blockchain due to their hidden and distributed nature.

We have tried to make this uncertainty clear throughout the report. The carbon footprints calculations in this report serve as approximations indicating the order of magnitude and important trends in emissions that can guide decision-making about the effects that different courses of action could have on climate change. Furthermore, the lack of coherent standards for carbon accounting leads to different approaches, scopes and assumptions being used by different studies. We have attempted to make these explicit and compare the different methodologies used by the key studies reviewed in Section 2.2.

For reasons outlined above, emission estimates are more uncertain than estimates of electricity consumption. Nevertheless, an assessment of ICT's climate change impact needs to focus on GHG emissions rather than electricity consumption alone because it is emissions that ultimately drive climate change, and electricity consumption itself does not capture the impact of factors such as energy source mix and emissions in the energy generation supply chain. Since most studies focus on ICT's energy consumption, we felt that we could most usefully contribute to the scientific debate by applying our expertise in supply chain emission accounting to clarify some of the complexities around the emission footprint from energy use and other sources of GHG.

B Estimates of ICT Emissions

B.1 Historical Estimates of ICT's GHG footprint

***Table B.1** Historical estimates of ICT's GHG footprint. Unless otherwise stated, all estimates include embodied (based on LCAs) and use phase GHG emissions.*
**Based on 670 TWh in 2007 and 930 TWh in 2012 [Lannoo et al. 2013] and 0.68 $MtCO_2e$/TWh (SWC estimate).*
***Based on 655 TWh in 2007 and 909 TWh in 2012 [Van Heddeghem et al. 2014] and 0.68 $MtCO_2e$/TWh (SWC estimate).*

Study	Year	$MtCO_{2e}$	Scope for emissions
Gartner [2007]	2007	620	CO_2 emissions only; use phase and emissions for phones, PCs, printers, data centres and networks
GeSI [2008]	2002	530	Desktop PCs and laptops and PC peripherals (monitors, printers), data centres, telecoms networks and devices
	2007	830	
	2020	1430	
Malmodin et al. [2010]	2007	1,150	Phones, PCs, modems, networks and data centres (630 $MtCO_2e$); TVs, TV peripherals and TV networks (390 $MtCO_2e$); other E&M equipment, including audio devices, cameras and gaming consoles (130 $MtCO_2e$)
GeSI [2012]	2011	910	PCs (desktops, laptops), mobile devices (tablets, smartphones, regular mobile phones), and peripherals (external monitors, printers, set-top boxes, routers, IPTV boxes); fixed and wireless networks (excluding local WiFi networks), data centres (servers, storage and cooling)
	2020	1270	
Lannoo et al. [2013]*	2007	454	Emissions from electricity and use phase only; computers, data centres, networks
	2012	630	
Malmodin et al. [2013]	2020	2,200	Phones (fixed, mobile), PCs (desktops, laptops), modems, networks and data centres (1,100 $MtCO_2e$); TVs, TV networks and TV peripherals (1,100 $MtCO_2e$); other E&M equipment, including audio devices, cameras and gaming consoles (420 $MtCO_2e$)
Van Heddeghem et al. [2014]**	2007	444	Emissions are use phase electricity only; desktops, laptops, monitors, networks and data centres
	2012	616	
Malmodin [2019]	2010	720	Phones (fixed, smartphones, other mobile), tablets, PCs (desktops, laptops), displays, modems, some IoT, networks and data centres

B.2 Detailed Review of the Key Studies

This report focuses on reviewing peer-reviewed studies by three main research groups published from 2015 that estimate ICT's carbon footprint from 2015 onward. Here, we

include a summary of the studies scope and assumptions (B.2.1), then follow with an overview of estimates (B.2.2) and a detailed review of relevant studies by researchers around Andrae (B.2.3), Belkhir (B.2.4) and Malmodin (B.2.5).

B.2.1 Overview of scope and methodological differences

Studies on the energy and carbon footprint of IT can be classed as either bottom-up (based on LCAs, energy use reports for certain devices and company reports, combined with data on the number of devices produced and used in a given year and the number of network subscriptions), or top-down (based on national or global statistics and input-output analysis). The latter is often difficult to obtain. Most studies use a bottom-up approach in combination with some top-down data, for example combining LCAs for user devices with global statistics for data traffic, such as from Cisco. Using a combined method is probably the best approach to assess emissions accurately.

The studies reviewed for this study use different methodological approaches. Some only include emissions from electricity (e.g. A&E), presenting a more limited picture, while others also include other sources of GHGs (e.g. B&E; Malmodin's research), such as fossil fuel backup power for data centres, fuels used by vehicles and other sources of emissions in the process of mining.

All the key studies include use phase emissions but studies vary as to the other lifecycle stages considered. On one end of the spectrum, in addition to the use phase, A&E only include production energy, just one aspect of embodied emissions. On the other end, Malmodin's research includes end of life emissions, that is, the emissions associated with waste management. The stages of the equipment lifecycle covered by the different studies in this review as well as the scope and assumptions applied are summarised in Table B.*2*.

Table B.2 *Scope matrix for studies included in this review. T&D = Transmission and distribution losses in electricity grids. Note that Malmodin includes some 'Other digital technologies or trends', specifically: wearables such as smart watches and fitness trackers, smart energy meters, control units, surveillance cameras, public displays, payment terminals and the internet-connected communication device in vending machines.*
**Included in the E&M sector estimates, not ICT estimates.*

Component of ICT sector	Andrae and Edler (2015)	Belkhir and Elmeligi (2018)	Malmodin and Lundén (2018)
**User devices**			
Smartphones	✓	✓	✓
Nonsmart mobile phones	✓	✕	✓
Fixed phones	✕	✕	✓
Tablets	✓	✓	✓
Phablets	✓	✓	✕
Laptops/Notebooks	✓	✓	✓
Desktop PCs	✓	✓	✓
Displays	✓	✓	✓
Computer peripherals (e.g. mouse and keyboard)	✕	✕	✓
Projectors	✕	✕	✓*

Cameras	✗	✗	✓*
Home media players/audio systems/traditional speakers	✓	✗	✓*
Portable media players, e.g. iPods	✗	✗	✓*
Smart speakers	✗	✗	✗
Smart watches/fitness trackers	✗	✗	✓*
Headphones/Earphones	✗	✗	✓*
Game consoles	✓	✗	✓*
Arcade game machines	✗	✗	✓*
Customer premises equipment (routers, modems)	✓	✓	✓
**Networks**			
Fixed telephony	✓	✓	✓
Mobile	✓	✓	✓
Fixed access wired	✓	✓	✓
Fixed access WiFi	✓	✓	✓
Enterprise networks	✗	✓	✓
Lower power, lower bandwidth device networks for IoT	✗	✗	✗
**Data centres**			
Servers	✓	✓	✓
Buildings that house servers	✗	✓	✓
Cooling	✓	✓	✓
Backup power supplies	✗	✓	✓
Operator activities, such as offices, business travel, maintenance of equipment	✗	✗	✓
**TVs, TV peripherals and TV networks**			
TVs	✓	✗	Yes*
Set top boxes	✓	✗	Yes*
Aerials	✗	✗	-
Satellite dishes	✗	✗	Yes*
DVD/BD players	✓	✗	Yes*
TV networks	✗	✗	Yes*
>>Cable	✗	✗	Yes*
>>Satellite	✗	✗	Yes*
>>DTT	✗	✗	Yes*
**Other digital technologies or trends**			
Cryptocurrencies/Blockchain	✗	✗	✗

AI/Machine Learning	✗	✗	✓
IoT	✗	✗	✓ (some)
Satellites	✗	✗	✗
Radio (device+networks)	✗	✗	✗
Embedded devices, e.g. sensors for smart cities, smart home tech, M2M communication	✗	✗	✓ (some)
Private internet, e.g. for military purposes	✗	✗	✗
**Trends considered for future projections**			
Blockchain	✗	✗	✗
Artificial Intelligence/Deep learning/Machine Learning	✓	✗	✗
IoT	✓	✗	✓
Video	✓	✗	✗
**Assumptions**			
Electricity carbon intensity (kgCO_2e/kWh)	Varies by scenario; 0.61 in 2015; 0.6-0.61 in 2020; 0.55-0.65 in 2030	0.5	0.6
Aspects of electricity likely covered (inferred from number)	generation, well-to-tank and T&D losses	generation only	generation, well-to-tank and T&D losses
Use phase included	Yes	Yes	Yes
Embodied included (based on LCAs)	Yes	Yes	Yes
Embodied carbon included	Production electricity only; no transport or end of life considered and no other sources of GHG other than electricity	Material extraction and manufacturing energy, not transport and end of life	Material acquisition, parts and component production and assembly, transport and end of life

B.2.2 Estimates for ICT's GHG Emissions in 2015 and 2020

Table B.**3** below estimates summaries by the key studies for 2015 and 2020.

Table B.3 *Estimates of GHG emissions from the ICT sector in a) 2015 and b) 2020.*

GHG emissions from ICT in 2015 ($MtCO_2e$)	User devices	Data centres	Networks	Total without TV	TVs	Total with TV
M&L 2015	395	160	180	733	420	1,153
B&E 2015 minimum	290	281	204	775	-	N/A
B&E 2015 maximum	485	281	204	971	-	N/A
B&E 2015 average (calculated)	388	281	204	873	-	N/A
A&E 2015 best case	186	213	190	589	329	917
A&E 2015 expected case	324	441	287	1,052	463	1,515
A&E 2015 worst case	514	582	454	1,550	706	2,257
**GHG emissions from ICT in 2020 ($MtCO_2e$)**	**User devices**	**Data centres**	**Networks**	**Total without TV**	**TVs**	**Total with TV**
Malmodin 2020	392	127	168	690	400	1,090
B&E 2020 minimum	343	495	269	1,107	-	N/A
B&E 2020 maximum	542	495	269	1,306	-	N/A
B&E 2020 average	443	495	269	1,206	-	N/A
A&E 2020 Best case	201	216	206	623	264	887
A&E 2020 Expected case	369	448	631	1,448	413	1,860
A&E 2020 Worst case	790	1,001	1,251	3,042	711	3,634

The studies vary in the scope with B&E only including user devices, data centres and networks, A&E including TVs and M&L including other consumer electronics such as cameras and audio systems in a car and portable GPS. In order to make estimates more comparable, we have brought them to the same 'system boundary', by adding Malmodin's estimate for the E&M sector (400 $MtCO_2e$; excluding print media) to B&E's and A&E's estimates (after subtracting emissions from TV from A&E's total estimates, using information provided in their supplementary information). The results are shown below. Considering that Andrae judges his Best case to be most realistic for 2020

[personal communication], the most likely range is 1.0-1.7 $GtCO_2e$ for ICT, TVs and other consumer electronics in 2020; this is 1.8-2.9% of global GHG emissions.

Table B.4 *Estimates of GHG emissions from the ICT sector in 2020 after adjusting for scope to include TVs and other consumer electronics.*

	$MtCO_2e$	Share of total GHG emissions
Malmodin 2020	1,090	1.9%
B&E 2020 Minimum	1,507	2.6%
B&E 2020 Maximum	1,706	2.9%
A&E 2020 Best case	1,023	1.8%
A&E 2020 Expected case	1,848	3.2%
A&E 2020 Worst case	3,442	5.9%

B.2.3 Research by Andrae and colleagues

Approach

Andrae and Edler [2015], from here on A&E, used a hybrid top-down bottom-up approach to model the production and use phase electricity use of user devices, networks, data centres and TVs between 2010 and 2030. User device emissions are modelled bottom-up from predicted production numbers and estimates for production and use phase energy use derived from LCAs. Estimates for the use phase electricity consumption of data centre and network are based on top-down data traffic trends based on Cisco data and estimates for electricity per data unit from the literature, while estimates for production electricity use are based on a fixed share of total electricity use by networks and data centres (5%, 10% and 15% for best, expected and worst case, respectively) – a method that seems somewhat imprecise. Their model also considers changes in energy efficiency (1% annually in the worst case, 3% in the expected case and 5% in the best case) and in electricity carbon intensity based on projected share of renewables which vary by year and scenario.

Findings

A&E's estimates for 2030 vary by a factor of 13, yet all scenarios show an increase relative to 2020 (see Figure B.1 Andrae and Edler's projections for GHG emissions from ICT by year.). While a growth trend in data traffic underlies the increase in total emissions, the large uncertainty in the size of this trend leads to the wide range of estimates. While the footprint of user devices is becoming less important, partly due to a shift from desktops and laptops to smaller devices like smartphones, and networks and data centres will contribute an increasing share of the total emissions over the next decade, due to the increase in data traffic. A&E argue that this growth in data traffic is driven by the popularity of video streaming, especially over mobile data, and emerging new data-heavy technologies, such as cloud computing. In more recent papers [2019a, 2019b, 2019c], Andrae also argues that AI and deep learning, IoT, Blockchain, virtual and augmented reality, facial recognition, and the rollout of 5G could lead to an explosion of data traffic over the next decade. In addition, IoT devices could increase the production footprint of ICT.

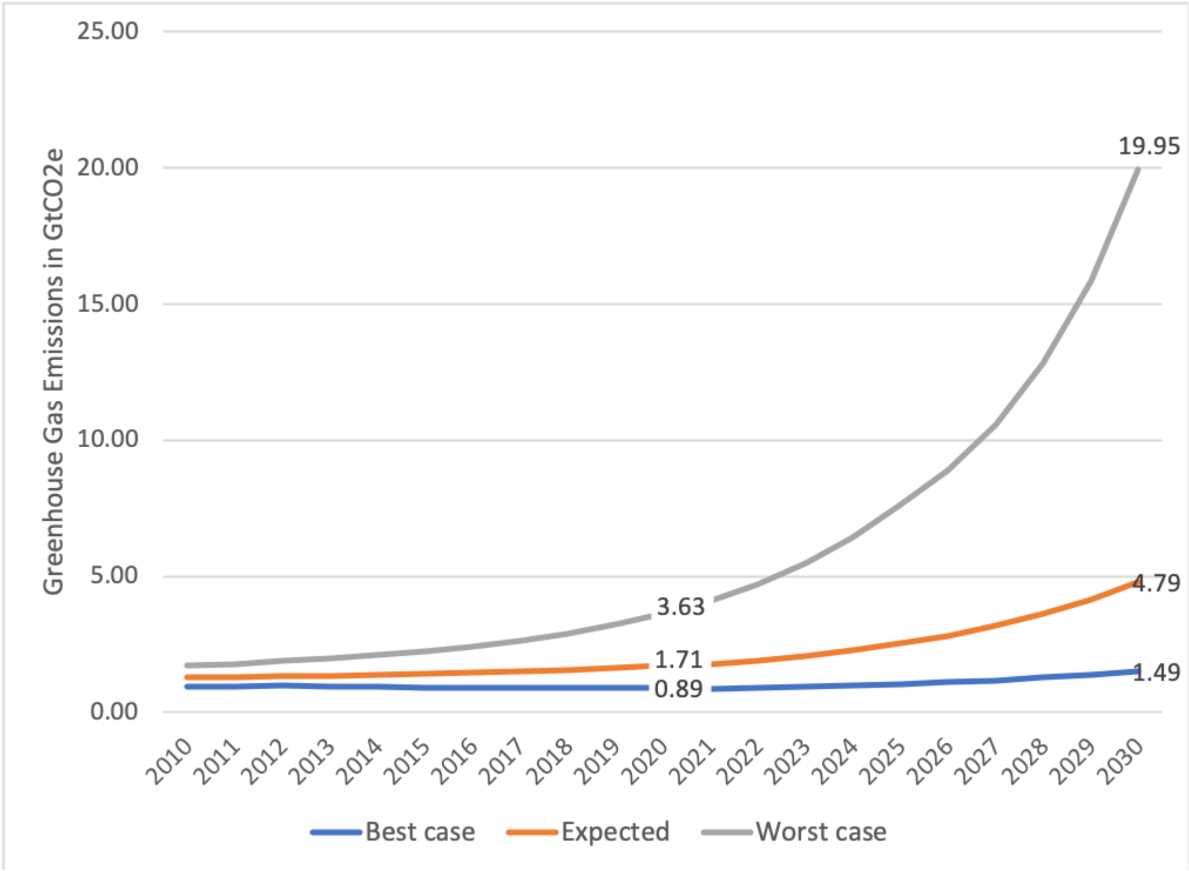

Figure B.1 *Andrae and Edler's projections for GHG emissions from ICT by year.*

A&E's worst case paints a dark picture and has been criticised as unrealistic by B&E and Malmodin [personal communication]. In personal communication, Andrae noted that A&E's study overestimated the carbon footprint of fixed wired and WiFi networks quite much even in the best case but underestimated mobile networks. Thus, for wireless and fixed access, the best case is the most relevant while for mobile, the expected case is the most likely. In 2019c, Andrae also notes that he overestimated production electricity of networks and data centres and that the ratio of production electricity to use phase electricity should be 2% instead of 5% used for the best case. Andrae's revised estimates for 2020 and 2030 (see Table B.**5**) are close to the best case scenario, partly thanks to increasing awareness of ICT's large energy footprint. But while ICT is saving electricity, those savings are used for further expansion, such as in cloud computing. If the trends discussed above take off unexpectedly, Andrae believes that data centre electricity use could be more than 4000 TWh in 2030 [personal communication].

*Table B.5 Andrae's revised estimates for 2020 and 2030 [personal communication], based on Andrae [2019b, 2019c, 2020]. Andrae uses an electricity carbon intensity of 0.55 $MtCO_2e$/TWh in 2020 and 0.54 $MtCO_2e$/TWh in 2030. Consumer devices including WiFi modems and TVs. *Use phase only*

Year	2020			2030		
**Metric**	**TWh**	**Range of $MtCO_2e$**	**Avg. $MtCO_2e$**	**TWh**	**Range of $MtCO_2e$**	**Avg. $MtCO_2e$**
Consumer devices*	600-1000	330-550	440	400-1000	216-540	378
Networks*	200-270	110-149	129	330-870	178-470	324
Data centres*	290-300	160-165	162	600-1000	324-540	432
Production of the above	250-380	138-209	173	180-300	97-162	130
	**1300-1900**	**715-1045**	**880**	**1500-3200**	**810-1728**	**1269**

In summary, Andrae believes that ICT's carbon footprint will continue to grow if not for major breakthroughs, albeit at a lower rate for the next few years than previously estimated. While the absolute total will increase, ICT's share of global electricity might stay stable if there are interventions or breakthroughs but this is unlikely under business as usual. Emissions might only reduce if data centres and production facilities are run entirely on renewable energy and if data intensity grows slower than expected [Andrae 2020]. Andrae [2020] notes that there are several potential 'engineering tricks' that might uphold efficiency gains even though Moore's Law has ended, such as decreasing semiconductor use stage power and nanophotonics, but it is unclear to what extent they have already been exploited.

Limitations and Criticism

One important difference to the other studies reviewed here is that only production electricity is included but not transport and material extraction, nor other sources of GHG other than electricity. Using the example of smartphones, they estimate between 18.8 (best case) and 37.5 (worst case) $kgCO_2e$ in 2010 but with efficiency gains, this decreases to 14.1-35.0 $kgCO_2e$ in 2015 and 10.6-33.0 $kgCO_2e$ in 2020. The assumption of decreasing embodied emissions for devices is problematic because smartphones are getting bigger and computationally more powerful, counteracting efficiency gains and greening of the electricity grid. In comparison, M&L estimated 45 $kgCO_2e$ for the average smartphone embodied footprint and B&E estimated 24.5-45.3kg. Thus, the study likely underestimates the embodied carbon footprint of ICT.

A&E's study has also been criticised by B&E for using a variety of device lifetimes in their scenarios that are not based on the published literature (e.g. 1, 2 and 3 years for smartphones, nonsmartphones, phablets and tablets for worst, expected and best case, respectively), thereby increasing the variance of embodied emission estimates.

In their calculations, A&E use the same number of device units for both the production and operational energy. In a sense, they are calculating how much electricity was used to produce all devices in use in a given year, regardless of when these devices were produced. That assumption is flawed as the same energy efficiency assumptions are applied to all devices used in a given year, even though older devices produced in earlier years will have not benefitted from these efficiency gains. Their figures might therefore underestimate the production energy for user devices further.

However, this paper has to be credited as the most transparent of all the papers reviewed, as the authors lay out clearly their assumptions and calculations in the supplementary information, broken down to the individual device and network type for every year between 2010 and 2030. It is also well-grounded in the previous published literature. The biggest criticism is probably the wide range of projections which leads to a difference between the best and worst case by a factor of 13.4; that said, a high degree of uncertainty does exist especially in such a rapidly developing sector as ICT.

B.2.4 Research by Belkhir and colleagues

Approach

Belkhir and Elmeligi (2018), from here on B&E, used a bottom-up approach for user devices and a top-down approach for data centres and networks and for total projections beyond 2020. The footprint of user devices is calculated by multiplying the number of phones sold in a given year by the lifecycle annual emissions, including embodied carbon spread over the expected lifetime and annual electricity consumption. One of the strengths of this study lies in the systematic review of useful life estimates in the literature for user devices which is more rigorous and provides a smaller range of estimates for user device embodied footprints than A&E more arbitrary useful life estimates. Data centres estimates are based on data from 2008 by Vereecken et al. [2009] and network estimates are based on 2008-2012 data from Van Heddeghem et al. [2014] which B&E projected to grow linearly.

B&E provide a breakdown of emissions for user devices, data centres and networks for each year 2007-2020, but model only the total carbon footprint of ICT from 2020 to 2040 by fitting a linear and an exponential growth curve to their estimates of total emissions from 2007 to 2020.

Findings

B&E estimate ICT's footprint in 2020 at between 1.11 $GtCO_2e$ (minimum) and 1.31 $GtCO_2e$ (maximum). The range is considerably smaller than in A&E and is due to uncertainties in the carbon footprint of user devices, mainly desktops and displays. Data centre and network estimates are the same for both minimum and maximum estimates. The authors suggest that including TVs could add another 435 TWh for operational energy use alone (assuming a global electricity carbon intensity of 0.6 $MtCO_2e$/TWh, this would add 261 $MtCO_2e$), assuming a 2% growth per year in number of TVs.

With regards to projections beyond 2020, both the linear and exponential curve show an increase (see Figure 4 in Section 2.1.2). The authors note that exponential growth, which would lead to between 2.48 and 2.62 $GtCO_2e$ in 2030 and 5.1 and 5.3 $GtCO_2e$ in 2040, is the most realistic, and that growth is highly likely if business as usual

continues. These predictions necessarily assume that trends active over the last decade continue for the next two decades and they assume unchecked growth. With an explosion of data traffic driven by trends like AI, Blockchain and IoT and the slowing down of efficiency improvements, there could be an additional jump in ICT's emissions within the next 3-5 years and an overall higher growth in emissions than modelled in their projections. However, while data centres will likely increase in power consumption, they might decrease in GHG emissions, if the trend of powering them with renewable energy continues [Belkhir in personal communication].

The predictions beyond 2020 are limited to totals and are not broken down by component but in their paper, the authors discuss the trend of wireless and mobile communications, cloud-based computing and IoT driving increases in data centres and networks. One of B&E main findings is the disproportionate impact of smartphones, whose footprint they estimate at 125 MtCO2e in 2020.[1] Most of this is due to their embodied footprint which is a concern in combination with their short average lifetime of 2 years. As many online data-heavy activities such as social media and video streaming are accessed by consumers on their mobile phones, the emissions associated with the data centres behind platforms like Facebook can be seen as a knock-on effect of mobile phone usage [Belkhir in personal communication].

Limitations and Criticism

B&E included the production and operational energy of ICT for user devices but they only considered the operational energy of data centres and networks, ignoring their embodied carbon because they found it to be negligibly small and excluding operator activities, potentially leading to a slight underestimate of total emissions.

B&E do not consider efficiency improvements in their estimates. For user device footprints this assumption might hold approximately as devices are 'upgraded' with more functionalities and a correspondingly higher footprint. However, for data centres, their estimates are based on extrapolation from 2008 emissions from data centres with a power usage effectiveness of 2, much higher than most modern data centres, without adjusting for efficiency improvements. This might explain why B&E's data centre estimate of 495 $MtCO_2e$ in 2020 is at the higher end relative to the other studies discussed here. Belkhir himself noted that their projection for data centres in 2020 is overestimated as efficiency improvements have unexpectedly been able to keep up with growing demand, even though this counterbalancing effect will soon come to an end as efficiency improvements slow down according to Belkhir.

While transparent about their sources, all peer-reviewed articles and publicly accessible industry reports, they did not make available supplementary data with the raw data for the total carbon footprint of ICT (with the exception of figures for 2020, 2030 and 2040) and user devices by year or their calculations. However, in personal communication with the lead author, we were able to get access to the raw data.

---

[1] This is based on 3.6 billion smartphones, including phablets, in 2020. Note that B&E did not include traditional mobile phones or fixed phones in the study's scope. B&E estimate 5.6 billion mobile phones to be in operation in 2030 and 8.7 billion in 2040, although data from Cisco [2020] suggests that the number of mobile phones could rise to 8.3 billion in 2023 already so there might actually be an even steeper rise in carbon emissions from smartphones.

B.2.5 Research by Malmodin and colleagues

Approach

Malmodin and Lundén [2018], hereafter M&L, use a hybrid top-down/bottom-up approach and draw on primary industry data from major manufacturers, sales statistics and LCAs for equipment. Emissions of user devices were modelled: 1) bottom-up based on the number of shipped and in-use devices in 2015 and the embodied and use phase emissions per unit estimated in LCAs, and 2) top-down the energy and carbon footprints of 35 major ICT and E&E manufacturers (reported in the supplementary materials) which were extrapolated based on those companies' share of revenue. Embodied footprints of user devices are the most uncertain part of their study.

Network footprints are based on a top-down analysis of network electricity consumption published by the authors [Malmodin and Lundén 2018b] which draw on anonymised operator data covering 70% of subscriptions, which was extrapolated to the global level. Data centre emissions are based on a mix of public and anonymised operator data. The authors note that it was not easy to get hold of primary data from networks and data centres operators because it is considered competitive. The operator data are therefore anonymised; however, they report data collected from public reports in the supplementary materials.

M&L only estimated ICT's global emissions for 2015 but in personal communication, Malmodin has shared his more recent and yet unpublished estimates for 2018 and 2020 with us (see Table B.**6** and Table B.**7**) which follow the same approach as M&L but are based on more recent operator data. We are also drawing on a presentation given by Malmodin at Energimyndigheten in 2019 [Malmodin 2019].

Table B.6 *Breakdown of ICT's carbon footprint as provided by Malmodin [personal communication].*

$MtCO_2e$	2015	2018	2020
ICT without TV	730	705	690
Networks	182	173	168
Data centres	141	129	127
Enterprise networks	17	16	15.5
User devices	392	380	375
TVs, TV networks and other consumer electronics	420	N/A	400
ICT with TV	1,153	N/A	1,087

Table B.7 *Operational electricity consumption by the ICT industry as provided by Malmodin [personal communication]. The total is adjusted for double counting.*

Operational TWh	2015	2018	2020
ICT Total	803	836	859

Networks	222	243	257
Data centres	220	225	230
Enterprise networks	25	25	25
User devices	343	349	353

Findings

Malmodin and colleagues argue that ICT's global emissions have broadly stabilised; they increased slightly from 720 $MtCO_2e$ in 2010 to only 733 $MtCO_2e$ in 2015 and decreased to 690 MtCO2e in 2020 (this includes user devices, networks and data centres). Relative to 2015, emissions from data centres have decreased the most (by 10%) followed by enterprise networks (9%) and networks (8%), even though electricity consumption increased in that time period due to the continued build out of 4G and 5G networks and an increasing number of data centre servers. The reason why the increased total energy use by ICT does not translate into higher carbon footprints is that it is partly offset by a higher share of renewable energy, a slight decrease in network overheads and that embodied carbon has stayed largely the same. The E&M sector (specifically, TVs, TV networks and other consumer electronics) add another 420 $MtCO_2e$ in 2015 [M&L] and 400 $MtCO_2e$ in 2020 (see Table B.**3**), showing a slight decrease too.

M&L further argue that ICT and E&M sector growth is starting to decouple from GHG emissions as ICT use is continuing to grow in terms of number of users and data traffic, albeit slower than previously as the world is moving towards saturation.[2] M&L argue that electricity consumption in data centre and networks does not follow the same exponential growth curve because of efficiency gains by servers. They show that the carbon footprint per subscription and per GB of data in networks has decreased fast since the 1990s and argue that while the number of networks users has increased by a factor of 10 and data traffic has increased 10,000 times between 1995 and 2015 if voice traffic is included, yet ICT's carbon footprint has only tripled during that time. They hold that ICT's footprint does increase with use but is better correlated with the number of users rather than data traffic. M&L acknowledge that Moore's Law has slowed down since 2012/13 but note that there usually a time lag until the effects are felt outside of research labs. In personal communication, Malmodin also noted that so far, efficiency improvements are continuing. The decoupling is also helped by enablement of emission savings in other industries, for example from print media as newspapers increasingly shift online, even though they admit that the effect still has to be seen in other sectors like transport.

These predictions stand in contrast to an earlier study [Malmodin et al. 2013] that projected ICT's footprint at 1.1 $GtCO_2e$ in 2020 plus an additional 1.3 $GtCO_2e$ for the E&M sector, including 680 $MtCO_2e$ for TVs and TV peripherals. This study argued that the increase in emissions would be driven mainly by an increase in the number of devices and therefore network subscriptions and data traffic, which will have been partly counterbalanced by energy efficiency improvements in networks, more efficient

[2] Malmodin [2019] argues that data traffic grew 70 times 1995-2000, 15 times 2000-05, 4 times 2010-15, 3 times 2015-20.

TVs, a shift from desktops to laptops and lower standby electricity consumption.

Explaining the discrepancy with earlier predictions, M&L argue that their earlier study was still based on older data which assumed the historical growth of PC and TV sales would continue, whereas more recent research takes into account the peak of PCs around 2011 and a slow decline of TV and tablet sales as well as better power management. The only PC type that is expected to increase is gaming PCs which have a higher carbon footprint. Consumer electronics sales (e.g. cameras, media players) are also declining thanks a move to smaller devices like laptops, tablets and smartphones and in particular the integration of functions into smartphones which *replace* older and less efficient user devices. There is also a shift from traditional storage devices (e.g. memory sticks) to cloud storage.

At the same time, they argue that M2M communication and IoT are only adding a very small footprint. In contrast to earlier studies by Malmodin [e.g. Malmodin et al. 2010, 2013], M&L's study included several IoT devices, including wearables, smart energy meters control units, surveillance cameras, public displays, payment terminals and the internet-connected communication device in vending machines. For 2015, they concluded that their impact on emissions was marginal. However, they did not include other now-common IoTs like smart speakers or any connected devices from other sectors, such as those embedded in vehicles, buildings or IoT used for military, medical, security and industrial purposes, other than those listed above, although they note that these are expected to add to GHG emissions in the future. The authors note that the number of IoT devices might explode and that M2M communication and therefore the number of network subscriptions are likely to increase rapidly in the future too. Already between 2010 and 2015, the number of M2M IoT subscriptions increased from 70 million in 2010 to 350 million in 2015.

A report by Ericsson [2019] presents a 2020 scenario which assumed a large increase in IoT and other new devices, with 500 billion sensors and tags, one billion connectivity boxes in ICT and 27 billion connectivity modules in other sectors, and found that their life cycle emissions were only minimal. The report argues that data traffic is driven more by video than by IoT. However, the methodology underlying this analysis is not provided.

Malmodin [2019] thinks that ICT can halve its emissions by 2030 relative to 2020 through renewable energy transformation and through collective effort. He expects data centres to be 1% of global electricity use even in the future, even though the absolute amount is expected to go up. In a recent Ericsson blogpost building on Malmodin's work, Lövehagen [2020] claims that ICT's carbon footprint could be reduced up to 80% if all electricity came from renewable energy. Importantly, she makes clear that ICT could be both a tool for decreasing or *increasing* global carbon emissions by accelerating carbon-intensive processes, depending on how it is used.

Another reason for stabilisation of emissions for Malmodin is his belief that there are limits to ICT's carbon footprint as smartphone markets saturate and there are a limited number of hours per day that users can use ICT equipment [personal communication]. Malmodin [personal communication] notes that the impact of AI and machine learning

is so far very small even though it has been around for a while, and that emissions from AI are unlikely to explode unless training AI becomes more efficient.

Limitations and criticism

M&L and Malmodin's follow-up research present the most recent research on global ICT emissions reviewed here and the one with the widest scope in terms of ICT equipment, lifecycle stages and supply chain emissions considered.[3] Unlike Belkhir's and Andrae's studies, the estimates are based on more recent and a wider range of measured data directly from industry rather than older data reported in the academic literature. This is valuable as trends in ICT can change fast.

However, this data is also the biggest weakness, as it is not made public and cannot be scrutinised by the reader. Apart from one LCA for smartphones [Ercan et al. 2016], LCAs for device embodied and use phase carbon are not based on peer-reviewed LCA studies but on data by market research companies like IDC, IHS and Gartner whose reports are not available for free, even through university subscriptions. They are therefore not easily accessible for the reader either.

In personal communication, Malmodin noted that data centre emissions might be overestimated by up to 13 MtCO_2e for 2020 if the use of green electricity is taken into account, even though the investigation is still ongoing. For 2015, he notes that M&L should have used 0.63 kgCO_2e/kWh instead of 0.6 kgCO_2e/kWh. This would change the use phase carbon footprint upward by 5%.

Malmodin has been challenged about his opinion that ICT's carbon footprint has reached a peak and that energy growth is slowing despite data traffic increases, as he believes energy use is largely unrelated to data traffic, and his assumption that energy efficiency gains will continue [Belkhir and Andrae in personal communication]. This debate is examined in more detail in Sections 2.2.1 and 2.2.4.

B.3 Drivers of change in ICT Future Emissions

A&E predict that over the next decade, user devices will become less important and networks and data centres will become more important for ICT total emissions. This is partly due to: a shift from desktops and laptops to phones; an increase in data traffic through trends like video streaming, cloud computing and emerging new data-heavy technologies; and a higher share of mobile data transmission because of the popularity

[3] Unlike A&E and B&E, M&L consider all stages of ICT equipment lifecycle, that is material acquisition, parts and component production and assembly, transport and end of life. In addition to 'classic' ICT, they include the entertainment and media (E&M) sector, which includes TVs and other consumer electronics, such as cameras and audio systems in a car and portable GPS, e.g. for use in cars (see Table B.2). The study also includes operator activities and overheads, such as offices and business travel used by data centre and network operators, and enterprise networks, which are wireless and wired networks within business buildings that are operated by the company. The network emissions include the embodied carbon of infrastructure, like digging cable ducts and constructing antenna towers spread over its lifetime. ICT used by the financial system is also in scope, including computers, TVs, networks and servers, but not cryptocurrencies with the rationale that mining cryptocurrencies required *specific* hardware, not regular servers. This rationale has been challenged by Belkhir [personal communication] as unreasonable as mining computers and servers use GPUs, which are found in gaming and are therefore within the scope of ICT. AI is included indirectly by covering all data centre emissions with a top-down approach.

of smartphones. In more recent papers [Andrae 2019a, 2019b, 2019c], Andrae also argues that trends such as AI and deep learning, IoT, Blockchain, virtual and augmented reality, facial recognition, and the rollout of 5G could lead to an explosion of data traffic over the next decade—increasing the share of data centres in ICT's emissions. While the trend towards smaller devices like smartphones is helping reduce ICT's emissions, we are also adding more devices like smart speakers and IoT.

B&E point to the rising footprint of smartphones, with a share of 11% of total ICT emissions in 2020. As the number of users and the amount of data-heavy activities like video-streaming and social media on smartphones increases, this in turn contributes to an increase in mobile network and data centre use. Due to their short lifetime and increasing energy efficiency, the vast majority of smartphones' emissions are embodied. They predict that data centres' electricity use is going to rise but that emissions might stabilise if the trend of powering them with renewable energy continues. Network emissions are increasing slowly, and PCs' emissions are decreasing.

In contrast, M&L believe that AI and Machine Learning (ML) will not play a large role, unless training AI becomes more energy efficient because it would not be economical to run. Their assessment of IoT (even though with limited scope – see Appendix B.2.5) led them to conclude that the impact of IoT is, and will continue to be, minimal in the foreseeable future due to low data volumes; this is despite a possible explosion in the number of devices and network subscriptions. TV and PC emissions are decreasing due to better power management, lower standby power consumption and decreased sales. Their view is that other electronics are also declining – helped by a shift to smartphones which integrate functions such as video streaming, cameras, and portable media players, into one device. They highlight the large energy consumption of user access equipment that is on 24/7, such as modems, routers and set top boxes. They argue that the growth in data traffic is slowing down and that data centres and networks electricity consumption will not grow exponentially alongside the growth of data traffic because of efficiency gains and shifts to renewable energy.

B.4 Reports out of scope of the review

There have been several reports in recent years on the topic of ICT's emissions, including on behalf of the Global eSustainability Initiative (GeSI), which represents ICT companies. In their report SMART 2020, produced by The Climate Group, they estimated ICT's emissions at 530 MtCO_2e in 2002 and 830 MtCO_2e in 2007 and projected ICT's footprint to rise to 1,430 MtCO_2e in 2020 under business as usual. In a later report compiled by BCG, SMARTer 2020 [GeSI 2012], they estimated emissions in 2011 at 0.91 GtCO_2e and revised their 2020 projection to 1.27 GtCO_2e. Their latest report, SMARTer 2030 [GeSI 2015], compiled by Accenture, extends their earlier projections to 2030 with an estimate of 1.25 GtCO_2e. The scope is summarised in Table B.**1**; for the 2015 report, it also includes 3D printers. In their reports, they also discuss 'abatement potential' by ICT in other industries whereby ICT could save 9.1 Gt CO_2e in 2020 and 12.08 Gt CO_2e in 2030; we explore these trends in more detail in Section 2.2.5. Another report claiming emission reductions of 2.1 GtCO2e enabled by mobile technology was released by GSMA [2019], which represents mobile operators.

Policy Connect, a London-based thinktank, produced a report *Is Staying Online Costing The Earth?* [McMahon 2018] sponsored by Sony in 2018 that concluded that energy consumption by ICT is not necessarily going to rise due to efficiency gains, renewable energy, a trend to smaller devices and ICT-enabled carbon savings in other industries.

The report *Lean ICT – Towards Digital Sobriety,* produced by The Shift Project [2019b], a Paris-based thinktank, came to a very different conclusion. They projected that ICT emits between 2.1 and 2.3 $GtCO_2e$ in 2020 and between 3.3 and 4.2 $GtCO_2e$ in 2025, including embodied and use phase carbon for PCs, phones, tablets, TVs, some IoT, networks and data centres. The modelling is based on Andrae and Edler's (2015) study but with updated assumptions, such as data traffic and the number of devices used. These estimates lie between Andrae and Edler's expected and worst case and therefore much higher than what the three main experts whose papers we reviewed above believe. The report points to several important trends, such as the impact of video streaming and short-lifespan devices, the underestimation of ICT's emissions by consumers because the underling infrastructure is invisible, and the unequal distribution of data consumption with high-income countries benefitting and thus emitting more than low-income countries.

These reports have not been included in the detailed review as they are not peer-reviewed. In addition, there are potential conflicts of interest where reports are sponsored by ICT companies [e.g. McMahon 2018; GeSI 2015; GSMA 2019]. Policy Connect's report largely relies on M&L's study, which is included in our detailed review, rather than offering original insights. For The Shift Project's [2019b] report, new modelling was done based on A&E's study but the findings have been discredited by all of the experts consulted (including Andrae, Belkhir, Malmodin and Preist). In the case of GeSI, the modelling behind the report is not transparent and assumptions are not made clear so it cannot be fully assessed.

C Video Streaming

Video streaming has become the dominant driver of data traffic consumption - forming 60% of downstream traffic and 22% of upstream traffic globally in 2018 [Sandvine 2019]. This traffic demand has been driven by adoption of video-on-demand services offered by companies such as Netflix, Amazon Prime and Disney; the popularity of YouTube and the embedding of video clips into other online services (e.g. social media such as Facebook and Twitter); and the use of video for security surveillance and video conferencing.

If travel is fully replaced by video conferencing, video offers significant carbon savings.

Online video can most prominently provide opportunities for reduction in travel-related carbon emissions. For example, video conferencing for co-locating a conference can create significant emission reductions from flights [Coroama et al. 2013], creating dematerialisation if the potential of this media is *"actively sought and unleashed"* [Coroama et al. 2015]. Video streaming has shown how useful it is during the Covid-19 outbreak, allowing entertainment during isolation as well as supporting home working. During the pandemic: replacing physical face-to-face meetings, for example, will reduce the travel-based emissions from business flights and peoples' commutes to work; we have also seen academic conferences moving online. However, as highlighted in Section 2.2.5, the rise in video traffic and availability of video

conferencing has not yet led to a reduction in air travel [Graver et al. 2019], although this may change following the Covid-19 crisis.

Video is accelerating data traffic.

Video is clearly a prominent driver in data traffic which could significantly add to ICT's growth and emissions (Section 2.2.4). For example, higher streaming qualities such as High Definition (HD) and Ultra HD (UHD) can have a *"multiplier effect on traffic"*: 4K (UHD) doubles the bit rate of HD video and multiplies the bit rate of Standard Definition (SD) by nine [Cisco 2020]. Streaming qualities also affect device adoption, e.g. 66% of flat-panel TV sets are expected be UHD in 2023 (doubling the 33% share in 2018) [Cisco 2020], therefore impacting the embodied emissions of video-focused devices as users replace older TVs with newer models. In addition, faster infrastructure (e.g. 5G, fibre to the home) enables applications such as UHD cameras and VR streaming [Cisco 2020], multiple simultaneous streams within households [Widdicks et al. 2019], and now data-intensive gaming activities [Vaughan 2019] – driving the demand for video related network traffic and high performance streaming infrastructure such as content delivery networks and data centres further.

Changes are required to stop continuous video and internet infrastructure growth.

The Shift Project [2019a] estimated that 300 MtCO_2e was generated in 2018 due to online video and argue that this is comparable to annual emissions of Spain in 2010. These estimates have come under scrutiny [Kamiya 2020] due to arguments that they were based on old data, that energy impacts of the internet are much lower [cf. Shehabi et al. 2014] and that energy intensities of data transmissions are halving every two years [cf. Aslan et al. 2017] – following the 'Efficiency saves ICT' narrative (Section 2.2.2). These arguments also underpin some criticisms by TechUK [Fryer 2020] on a recent documentary BBC iPlayer [2020] "Dirty Streaming", arguing the documentary provides misleading or incorrect information on ICT's environmental impacts.

The Shift Project may overestimate absolute emissions due to the direct processing of video traffic – especially as the energy per bit does improve over time, there is evidence that data traffic, including video, links more potently to growth in infrastructure and capacity. Preist et al. [2016] argue that growth in the internet's infrastructure capacity allows for new data-intensive services and applications (of which video is a part) – offering new affordances to users, in turn driving demand for these services and therefore further infrastructure growth. Peak data traffic is one driver for this infrastructure growth due to increased demand for data-intensive services; other influences include overprovisioning the infrastructure to ensure these services are always available to all users even at peak times [Preist, personal communication]. Growth begets more growth, unless we put a ceiling on absolute demand. In addition, Belkhir [personal communication] highlighted that the agreement between Netflix (a major video streaming service) and EU regulators to ease Netflix's load on the network during the Covid-19 pandemic [Sweney 2020] makes it difficult to argue data traffic is not interlinked with ICT infrastructure growth.

This is where changes in online service design may have a positive impact, e.g. turning off the video for a large portion of YouTube users who are only *listening* to the content [Lord et al. 2015, Widdicks et al. 2019] can have comparable emission reductions to running data centres on renewable energy [Preist et al. 2019] — but much more will need to be done to mitigate the significant growth of video streaming.

D Narratives

The assumptions about efficiency improvements and demand for ICT and predictions about ICT's impact on emissions in the scientific literature and non-scientific reports and the media can be summarised in the form of six common 'narratives', as detailed below. Note that Rebounds in ICT, Rebounds stalled and Global Rebounds are theoretical possibilities for which there is some evidence in the scientific literature (Appendix I); however, they are not commonly discussed in the literature on ICT's emissions.

The first four narratives relate to ICT's own emissions, and the final two relate to ICT's impact on the rest of the global economy. The arguments underlying each narrative are underlined; we explore these in Section 2.2.

Efficiency saves ICT

Efficiency improvements are continuing; in combination with a shift towards more renewable energy, this will offset increases in ICT's energy use, stabilising ICT's emissions at the current level or even decreasing it in the future. Emissions are not so much influenced by the increasing data traffic but rather by the number of users, which will naturally level off soon as the world reaches saturation for personal ICT devices.

E.g. research by Malmodin and colleagues, Masanet and colleagues

Growth without efficiency

The growth in data traffic will lead to increases in network and data centre energy use, while the growth in IoT will lead to increases in embodied device emissions.

A) In combination with efficiency improvements slowing down, this will lead to an exponential growth in ICT's emissions.

B) Even if efficiency improvements continue, they will lead to further emission growth because of Jevon's paradox (Appendix I), unless emissions are capped.

E.g. research by Andrae and colleagues, Belkhir and colleagues

Rebounds in ICT

The efficiency improvements enabled by ICT in other sectors lead to system growth within ICT. Under current conditions, rebound effects are greater than 100%. Therefore, the net effect of efficiency through ICT's is a rise in global emissions. If efficiencies continue, ICT's emissions will also increase unless they are deliberately constrained.

Rebounds stalled

If efficiency improvements stall (for example because Moore's Law reaches its quantum limit), this will lead to a plateau of emissions because growth requires efficiency gains.

Enablement

Because ICT enables carbon savings in other industries, the net effect of ICT is to lower global emissions despite growth in the ICT sector's own footprint.

E.g. GeSI's SMARTer 2030, GSMA's The Enablement Factor report

Global Rebounds

ICT enables efficiencies in other sectors which lead to growth in the wider economy. Rebound effects are larger than the efficiency gains (i.e. greater than 100%) and lead to an overall increase in global emissions.

E Truncation Error

There are two core methodologies for estimating the embodied carbon: the more commonly used Life Cycle Analysis (LCA) and Environmentally Extended Input Output (EEIO) analysis. LCA has potential for greater specificity as it is tailored to specific models, such as an iPhone 11, but inevitably incurs a truncation error; an underestimation arising from LCAs being unable to include the infinite number of supply chain pathways. To illustrate, a factory manufacturing computers will itself use computers to manage the production, a small share of whose embodied carbon needs to be attributed to the factory's output. Most of the literature assessing the embodied carbon in ICT is LCA-based.

EEIO offers a much more generic estimate, based on macro-economic modelling of financial and carbon flows between industrial sectors. It provides estimates of the total carbon emissions resulting from production of different types of goods per unit of monetary value. Whilst lacking specificity (i.e. all goods within broad categories, such as 'manufacture of office machinery and computers', have the same carbon footprint per dollar), EEIO-based estimates have the important advantage of taking account of emissions from all supply chain pathways; they do not incur truncation error.

To get some of the best of both approaches, it is possible to combine LCA and EEIO methodologies by approximating and adjusting for the truncation error incurred by LCAs. This can be done by mapping the LCA's system boundaries onto the EEIO model. Such a hybrid methodological approach stands to have both the specificity of LCA and the system-completeness of EEIO. In this report, we have drawn upon work carried out by SWC (see Appendix A.5.4) to derive adjustment factors for LCAs in different product categories and applied these to LCA-based embodied carbon assessments to derive system-complete estimates.

Based on SWC's EEIO model, we estimate that truncation error causes an omission of ca. 40% of the total embodied carbon and ca. 18% of the use phase carbon. When this is factored in, adjusting each study's specific electricity intensity figures, estimates for 2020 are on average 25% higher. Table E.**1** shows A&E, M&L and B&E LCA carbon estimates without adjustment of truncation error; Table E.*2* shows the adjusted estimates when truncation error is taken into account. This is just an approximation. We reiterate the caveat that SWC's EEIO model is based on UK data which is not representative of the world economy yet we have applied it to A&E's, M&L's and B&E's global estimates for a rough estimate of underestimation. We also note that these studies likely incur truncation errors of different sizes due to their differences in methodology. We have only adjusted for these differences with respect to electricity carbon intensity, but not for embodied emissions. Due to its more inclusive scope, M&L's is likely to have a smaller truncation error and A&E likely has a larger one than the average truncation error assumed here.

Table E.1 Original estimates of embodied and use phase carbon for 2020. Malmodin's and A&E's estimates include TVs, B&E's estimates do not.

**Study**	**Embodied ($MtCO_2e$)**	**Use phase ($MtCO_2e$)**	**Total ($MtCO_2e$)**	**$MtCO_2e/TWh$**
Malmodin (2020)	300	787	1087	0.60
B&E - 2020 minimum	213	894	1107	0.50
B&E - 2020 maximum	349	957	1306	0.50
B&E - 2020 average (calculated)	281	926	1207	0.50
A&E - 2020 Best case	157	730	887	0.59
A&E - 2020 Expected case	326	1534	1860	0.59
A&E - 2020 Worst case	1024	2610	3634	0.61

Table E.2 Estimates for 2020 adjusted to include all supply chain pathways.
**Included in the average. A&E's best case was chosen because Andrae (2020) reported that this is the most realistic for 2020. An average calculated for B&E's minimum and maximum estimates was included sinde B&E did not endorse either scenario and we wanted to avoid skewing the average by considering two of their estimates.*

	**Embodied ($MtCO_2e$)**	**Use phase ($MtCO_2e$)**	**Total ($MtCO_2e$)**	**$MtCO_2e/TWh$**
Malmodin (2020)*	500	826	1326	0.63
B&E - 2020 minimum	355	1127	1482	0.63
B&E - 2020 maximum	582	1206	1788	0.63
B&E - 2020 average (calculated)*	469	1166	1635	0.63
A&E - 2020 Best case*	262	781	1043	0.63
A&E - 2020 Expected case	543	1628	2171	0.63
A&E - 2020 Worst case	1706	2704	4410	0.63
**Average**	**410**	**925**	**1335**	**0.63**

For ICT, TV and other consumer electronics, the adjusted total ranges between 1.2 and 2.2 $GtCO_2$ in 2020 (2.1-3.9% of global GHG emissions, see Table E.**3**) with 30% coming from embodied carbon and 70% from use phase on average.

Table E.3 Estimates for 2020 adjusted to include all supply chain pathways and brought to the same scope. *Included in the average.

	**Embodied ($MtCO_2e$)**	**Use Phase ($MtCO_2e$)**	**Total ($MtCO_2e$)**
Malmodin (2020)*	500	826	1326
B&E's Minimum + Malmodin's E&M figure	457	1482	1940
B&E's Maximum + Malmodin's E&M figure	684	1562	2246
B&E's Average (calculated) + Malmodin's E&M figure*	571	1522	2093
A&E's Best without TV + Malmodin's E&M figure*	296	898	1194
**Average**	**456**	**1082**	**1538**

This scope does not include some ICT equipment, such as radios, Blockchain and most IoT. Using EEIO, Livia Cabernard from ETH Zurich [Cabernard 2019, Cabernard et al. 2019] estimates that ICT's embodied carbon footprint (including manufacturing and transporting and covering a wider scope of ICT, specifically computers, mobile phones, TVs, radios, office machinery and all embedded ICT) was ca. 1.1 $GtCO_2e$ in 2015. Of the 1.1 $GtCO_2e$ embodied emissions, 27% were from computers, 55% from radio, TV and mobile and 18% from ICT embedded in other end products. Over the last few decades, a production shift to China (61% of ICT production in 2015, 45% in 1995) has increased the embodied carbon footprint of ICT because China's electricity use is mainly from coal [Cabernard in personal communication].

Modelling future population growth, efficiency improvements in relevant sectors, such as steel production, and a transition from coal to renewable energy in line with actions taken to limit global warming to 2°C (6°C), Cabernard [personal communication, based on Wiebe et al. 2018] predicts that ICT's embodied carbon footprint could be 1.38 (1.5) $GtCO_2e$ in 2020, 1.27 (1.56) $GtCO_2e$ in 2025 and 1.16 (1.64) $GtCO_2e$ in 2030 for embodied emissions of ICT manufacturing. That means that the estimates for embodied carbon in Table E.**3** only cover a third of ICT's true embodied carbon footprint, because some ICT equipment, such as radio, Blockchain and embedded ICT, is left out of scope.

In summary, in most research on ICT's emissions, ICT's embodied carbon is considerably underestimated. While users are keeping their PCs and smartphones for slightly longer, the manufacturing footprint of smartphones is increasing because of more advanced integrated circuits, displays and cameras [Malmodin in personal communication]. With the number of IoT devices predicted to grow exponentially, the embodied footprint of ICT is likely to increase in the future.

F European Commission’s Investment in ICT

F.1 Artificial Intelligence

The European Commission has ambitions to significantly increase uptake of AI as a way of *“strengthen*[ing] *the competitiveness of European industry”* [European Commission 2019c]. Specific initiatives and funding streams include Digital Innovation Hubs [European Commission 2018b] to *“provide support to SMEs to understand and adopt AI”* [NoCash 2020], and InvestEU (more on this below). The EC recently funded the 50 million Euro project “ELISE” which aims to *“make Europe competitive by setting up a ‘Powerhouse of AI’”* [FCAI 2020]. New Common European data spaces are also being set up to enable greater data sharing [Kayali et al. 2020], which may be used by AIs. The Commission also views AI as a *“driving force to achieve the Sustainable Development Goals”* [European Commission 2019c], so is funding *“competitions and missions for AI solutions tackling specific environmental problems”* [European Commission 2019d]. Examples of how AI is expected to produce emissions reductions in other sectors include *“increasing the efficiency of farming, contributing to climate mitigation and adaptation,* [and] *improving the efficiency of production systems through predictive maintenance”* [European Commission 2019c].

F.2 Internet of Things

The European Commission has launched the Alliance for Internet of Things Innovation to *“to support the creation of an innovative industry driven European Internet of Things ecosystem”* [European Commission 2019a]. Within the Digitising European Industry initiative, the Commission identifies three foci: 1) *“a thriving IoT ecosystem”*; 2) *“a human-centred IoT approach”*; and 3) *“a single market for IoT”*. The latter is facilitated through the *“European data economy”* initiative, which *“proposes policy and legal solutions concerning the free flow of data across national borders in the EU, and liability issues”* [European Commission 2019a]. The IoT is seen as playing a key role in the European response to climate crisis through providing an infrastructure to enable the ‘smart future’ (see, e.g. [ETIP SNET]), and for distributing energy consumption across smaller data centres [Gilmore 2018].

F.3 Blockchain

The European Commission has established the European Blockchain Observatory and Forum to *“accelerate Blockchain innovation and the development of the Blockchain ecosystem within EU and* [to] *help cement Europe's position as a global leader in this transformative new technology”* [EU Blockchain 2020]. Along with AI, Blockchain is the target of the 2 billion Euro joint European Commission and European Investment Fund InvestEU Programme. In addition, the European Blockchain Partnership [European Commission 2018a] commits all member states to *“realising the potential of Blockchain-based services for the benefit of citizens, society and economy”* [European Commission 2020]. As one of its potential benefits, Blockchain is viewed by the Commission as the kind of ‘disruptive technology’ required by the climate crisis [European Commission 2019b], and the Commission has outlined five core areas they seek to ‘unleash’ Blockchain technology in service of climate. Based on the idea that Blockchain can *“improve the transparency, accountability and traceability”* of GHGs, these application areas include areas of clean power, smart transport systems, sustainable production and consumption, sustainable land use, and smart cities and homes.

G Carbon Pledges

Company pledges vary in particular with regards to scope of emissions covered (see Table G.1), offsets used and how much emissions are reduced. Note that there are also some companies with plans to cut emissions by a certain percentage but without the sweeping ambitions of the below pledges.

Table G.1 Emissions covered by Scope 1, 2 and 3.

Scope 1 emissions	Direct emissions from burning of fossil fuels on site (includes company facilities and vehicles).
Scope 2 emissions	Indirect emissions from purchased electricity and gas.
Scope 3 emissions	All other indirect emissions in a company's value chain including upstream emissions in the supply chain (e.g. emissions from purchased goods and services, transportation of these goods to the company, use of leased assets such as offices or data centres, business travel and employee commuting) and downstream emissions (from transportation, distribution, use and end of life treatment of sold products, investments and leased assets). Scope 3 emissions form the majority of a company's emissions.

G.1 Carbon Neutral

Carbon neutral means no net release of carbon-dioxide emissions into the atmosphere, through using offsets to counterbalance any emissions generated.[4] For a company to be carbon neutral, they must measure, reduce, and offset their emissions.

There is no set standard on which (if any) scope 3 emissions should be included, how these emissions should be reduced (purchasing credits or on-site renewables, for example), and which offsets are robust and credible.

Companies pledging carbon neutral: Sky (since 2006), Google (since 2007), Microsoft (since 2012), and Apple (by 2030).

G.2 Net Zero

Net zero is defined by the Science Based Targets initiative (SBTi) as a company having no net climate change impact through GHG emissions in a company's value chain. This is achieved by reducing GHG emissions in the value chain and removing the remaining emissions through additional carbon removals. Companies setting a net zero pledge can register with the SBTi if their targets Net zero means no net climate change impact through GHG emissions in a company's value chain, achieved by reducing GHG emissions in the value chain and removing the remaining emissions through additional carbon removals. A net zero target is more ambitious than a carbon neutral target because it specifies that scope 3 emissions need to be included, while for carbon neutral, generally only emissions from business travel and waste are included but not other scope 3 emissions.

[4] Note that most ICT companies refer to carbon dioxide and other GHG when they use the term 'carbon neutral'. This is sometimes called 'climate neutral'.

Organisations can register their net zero targest with the Science Based Target initiative (SBTi) which certifies organisations whose emission targets are in line with the IPCC's recommendations that global warming be limited to 2°C, well below 2°C or more recently to 1.5°C [IPCC 2018, Pineda and Faria 2019]. According to the SBTi, for company pledges validated after July 8th 2020, only two thirds of scope 3 emissions need to be accounted for and scope 3 emissions targets 'generally need not be science-based'. A target needs to be set for reducing scope 3 emissions only if they make up over 40% of a company's total emissions [SBTi 2019]. However, the Carbon Trust believes that net zero should apply to 100% of scope 3 emissions [Stephens 2019].

Net zero is also more ambitious than carbon neutral regarding offsets. SBTi specifies that 'offsets must not be counted as emissions reduction toward the progress of companies' science-based targets' [SBTi 2020]. In the way Sky and Microsoft define net zero, any emissions that cannot be reduced need to be removed from the atmosphere rather than being avoided, whereas carbon neutral can use both emission removal offsets and avoided emission offsets [Sky Zero 2020, Smith 2020].

Companies pledging net zero: Microsoft (by 2030), Sky (by 2030), Amazon (by 2040), BT (by 2045) and Sony (by 2050).

G.3 Carbon Negative

Carbon negative (also sometimes described as 'carbon positive' or 'climate positive') is where an activity removes more emissions than it emits across an entire value chain. This goes further than both carbon neutral and net zero. There is currently no official definition or standards for this apart from the ones used by Microsoft for their carbon negative pledge [Smith 2020].

Companies pledging carbon negative: Microsoft (by 2050).

G.4 100% Renewable

100% renewable means that all of a company's power consumptions comes from renewable sources, such as solar, wind and hydro. It does not specify whether and how much the company's emissions should be cut. Unfortunately, often companies do not provide detail on whether they will generate additional renewable energy on-site, or buy renewable energy certificates, including unbundled REGOs which should not be seen as truly 100% renewable, in the authors' opinion (see Appendix I).

Companies pledging 100% renewable: Netflix (since 2018), Google (2017), Facebook (by 2020), Samsung (by 2020), Microsoft (by 2025), Sky (by 2020), Vodafone (by 2025), Apple (since 2018).

Please note: the achievement of these pledges is self-reported and not externally validated.

G.5 Why scope matters

Carbon neutral pledges are not enough for the world to limit global warming to 1.5°C because they do not require the company to account for scope 3 emissions, which form the majority of ICT's carbon footprint. Voluntary emissions reductions of company

Scope 1 and 2 emissions alone could theoretically be sufficient if every company in the economy played its part. However, without any kind of enforcement or reputational consequences, companies have a competitive advantage if they do not set of meet targets. In addition, companies could lower their scope 1 and 2 emissions by outsourcing carbon-heavy activities to suppliers, which would not decrease overall emissions. By signing up for scope 3 targets, companies take responsibility for their supply chain. They then have an incentive to encourage their suppliers to cut their emissions too, creating a snowballing effect in the economy.

H Renewable Energy Purchases

H.1 On-site Generation

On-site generation of renewable energy is the best option for purchasing renewable energy. This is because, with on-site generation, the company carries the set-up costs of the renewable energy project and there are fewer transmission and distribution losses than when electricity is sourced from the grid. It also ensures that additional renewable energy is created which is needed for a successful global transition away from fossil fuels.

H.2 PPA with Bundled REGOs

Companies can also buy a Power Purchasing Agreement (PPA) for renewable energy. PPAs do not cover the set-up costs of a renewable energy project (unlike on-site generation) but they pay for the cost of the power generation and receive a bundled Renewable Energy Guarantees of Origin (REGOs) that certifies that each 1 MWh of electricity purchased comes from a renewable energy project. REGOs ensure for each unit of renewable energy generated, only one company can claim the environmental benefits, avoiding double counting. PPAs ensure additionality because a company purchasing a PPA pays for additional renewable energy to be generated and fed into the electricity grid. They can also encourage the setup of new renewable energy projects because projects can attract funding from investors more easily with guaranteed buyers of PPAs lined up.

H.3 Unbundled REGO

Companies also have the option of buying an unbundled REGO without the actual energy to lower their scope 2 emissions. In this case, the environmental benefit of renewable energy gets separated from the energy itself. The company generating renewable energy can sell off the environmental benefit represented by the REGO but without selling the actual energy. The company buying the unbundled REGO can claim lower scope 2 emissions. Unfortunately, unbundled REGOs do not encourage greater power generation from renewable sources because the demand for REGOs is currently vastly outstripped by the supply thus making them very cheap. This means they are an ineffective instrument for investment into renewable projects [Scott 2019] and cannot claim additionality. Because unbundled REGOs cannot claim additionality and the company buying it lays a sole claim to the “greenness”, thereby not sharing it with other electricity users, unbundled REGOs raise the carbon footprint of the electricity grid [Hewlett 2017]. The separation also makes it harder to

track what renewable energy projects lie behind each certificate. A company wishing to reduce their Scope 2 emissions and become powered by 100% renewables should therefore look towards investing directly into renewable projects and PPAs in which bundled REGOs are purchased, crucially, with the underlying power.

I Jevons Paradox

In 1865, William Stanley Jevons predicted that as the UK's use of coal became more efficient, it would make coal more attractive and thereby would increase demand for coal rather than reduce it [Jevons 1865]. *Jevons Paradox* refers to a situation in which an efficiency improvement leads to an even greater proportionate increase in total demand, with the result that resource requirement goes up rather than down, as is often assumed. There is evidence that Jevons Paradox applies beyond coal [e.g. Alcott 2005, Sorrell 2009, Schaffartzik et al. 2014]. An example is the increased energy efficiency of new forms of lighting (such as electric lighting compared to gas lighting) which allowed lighting to be used more widely – increasing the total energy consumption from lighting. Another demonstration is the fact that electric trains are vastly more efficient than steam trains, let alone horses, yet the carbon footprint of land transport has continuously risen over the time period that these technological advances took place due to expanded use [Berners-Lee and Clark 2013].

While Jevons Paradox is linked with efficiency as the principal driver of rebound effects, the paradox is frequently linked more broadly to a wide range of socio-economic drivers leading to a perverse increase rather than decrease in input demand. Macro-economic models suggest that this 'backfiring' or *rebound effect* leads to savings being cancelled out completely on average and even *adding input* demand relative to previous levels through a variety of mechanisms.[5] At the global level, efficiency improvements in almost every aspect of life have gone hand in hand with rising energy demand and rising emissions.

It is sometimes argued that without the efficiency improvements, demand would have increased even further; this assumes that demand would rise independently of efficiency. It is also argued by some [e.g. GeSI 2015; UK Energy Research Council 2007] that rebound effects are less than 100% of the efficiency savings, but this often results from an incomplete consideration of rebound pathways, especially macro-economic effects. To assess the full impact of rebound effects, all parts of the economy and a longer timescale need to be considered. The only way to feasibly do this is to analyse the combined effect of all global efficiency gains in all sectors and to track this against global energy use. This analysis yields a total energy rebound averaging

---

[5] Jevons Paradox and rebound effects are explored in more detail in Berners-Lee and Clark's book *The Burning Question* (2013). Briefly, they argue that when we improve energy efficiency, the available energy becomes more productive and therefore more valuable, leading to increased use. This is because any energy saved bounces back as additional energy elsewhere, either because: 1) efficiency makes the use of the resource cheaper (e.g. lighting, cloud storage of more data than with traditional file storage), 2) the savings are spent on other activities with a carbon footprint, 3) lower resource use leads to lower prices which increases demand for the resource elsewhere, or 4) knock-on effects in other areas of the economy (e.g. when video conferencing enables forming relationships with people on the other side of the world, leading to more air travel to visit them). Resource use can also be displaced into another country (e.g. when burning of fossil fuel domestically is restricted to lower the country's emissions but fossil fuel is continued to be extracted for exports to other countries with fewer environmental concerns).

102.4% over the past 50 years (i.e. the annual global growth in energy use) [Berners-Lee and Clark 2013]. Despite the increasing utility per unit of energy, the world's energy use is increasing. The same holds true for emissions. Over the last 170 years, CO_2 emissions have been rising at 1.8% per year (with only temporary deviations on either side of that trajectory) [Berners-Lee and Clark 2013] alongside the growth of ICT, vast efficiency gains in ICT and other technological advances in other industries.

In terms of the ICT industry, it has been argued that it is through its increasing efficiency that computational power has risen and ICT has been able to become so important in society; the energy consumption of early computers would have been prohibitive for the scale of expansion we have seen over the last decades [Aebischer and Hilty 2015]. An analysis of dematerialisation by Magee and Devezas [2017] found evidence that, in the ICT industry, efficiencies in the material needed for a single product lead to either increasing performance or reduced prices and that this inadvertently leads to increases in demand, resulting in an increase in absolute material consumption. Silicon is one example as it holds a special place in information storage, transmission and computing. Other examples of rebound effects in ICT are provided by Gossart [2015], Galvin [2015] and Walnum and Andrae [2016]. Galvin [2015] estimates that rebound effects in ICT's energy use could range between 115% and 161% based on eight case studies, as efficiency is more than offset by increases in demand.

In addition to efficiencies within the ICT industry, ICT-delivered efficiencies can also have far-reaching effects in other industries – in what we will call *Global Rebounds*. In recent years, ICT has increasingly expanded into other sectors. Common examples include video conferencing technologies or online shopping which could reduce the need to physical travel or reading news on a smartphone. These have the potential to both decrease and increase environmental impact. Where these new technologies evolve to be more energy intensive than their alternatives (e.g. high-quality video streaming), where they are used *in addition* rather than as a substitute (e.g. e-books being used alongside paper books), or where they allow intensified activity or growth in other industries because they are cheaper, more productive or more convenient (e.g. more regular checking of news on a smartphone than with traditional newspapers leading to increased need for news production), the impact of the economy as a whole in terms of energy use, resource use or GHG emissions can increase [Court and Sorrell 2020].

In a systemic review of the direct and economy-wide impact of e-materialisation (such as e-publications, e-games, e-music etc.) on energy consumption, Court and Sorrell [2020] found that studies systematically neglect rebound effects. Most studies assume substitution of old technology with the new digital system where this assumption is not always justified, leading to overestimates of energy savings. Assumptions around the lifetime, the number of users, efficiency of user devices and the replacement of travel lead to a wide range of predictions from 90% decreases to 2000% increases in energy consumption. They conclude that there is no conclusive evidence suggesting significant current or future energy savings from e-materialisation. There is another aspect to efficiency: psychological spillovers through moral licensing where people feel that they have done their part for the environment when increasing efficiency and then go on to have an increased environmental impact elsewhere [Sorrell et al. 2020] - but this is out of scope for this report.

The net effect of ICT depends on the balance of impacts it has both through its own emissions and the effects it has on the wider economy. The economy-wide effects of ICT are difficult to quantify, but in the absence of solid evidence, it would at the very least be risky to assume that the Jevons Paradox and other rebound effects (e.g. time rebounds [Börjesson Rivera et al. 2014]) do not apply to ICT's direct and economy-wide impact.

References

1. Aebischer, B. and Hilty, L.M., 2015. *The energy demand of ICT: a historical perspective and current methodological challenges*, in *ICT Innovations for Sustainability*. Springer. p. 71-103.
2. Alcott, B., 2005. *Jevons' paradox.* Ecological economics, **54**(1): p. 9-21.
3. Andrae, A.S., 2019a. *Prediction Studies of Electricity Use of Global Computing in 2030.* Int J Sci Eng Invest, **8**: p. 27-33.
4. Andrae, A.S., 2019b. *Comparison of Several Simplistic High-Level Approaches for Estimating the Global Energy and Electricity Use of ICT Networks and Data Centers.* International Journal, **5**: p. 51.
5. Andrae, A.S., 2019c. *Projecting the chiaroscuro of the electricity use of communication and computing from 2018 to 2030.* Researchgate. net.
6. Andrae, A.S., 2020. *Hypotheses for primary energy use, electricity use and CO2 emissions of global computing and its shares of the total between 2020 and 2030.* WSEAS Transactions on Power Systems.
7. Andrae, A.S. and Edler, T., 2015. *On global electricity usage of communication technology: trends to 2030.* Challenges, **6**(1): p. 117-157.
8. Aslan, J., Mayers, K., Koomey, J.G., and France, C., 2018. *Electricity intensity of Internet data transmission: Untangling the estimates.* Journal of Industrial Ecology, **22**(4): p. 785-798.
9. BBC iPlayer, 2020. *Dirty streaming: The internet's big secret*. https://www.bbc.co.uk/news/av/stories-51742336/dirty-streaming-the-internet-s-big-secret - accessed March 2020.
10. Belkhir, L. and Elmeligi, A., 2018. *Assessing ICT global emissions footprint: Trends to 2040 & recommendations.* Journal of Cleaner Production, **177**: p. 448-463.
11. Berners-Lee, M. and Clark, D., 2013.*The Burning Question: We can't burn half the world's oil, coal and gas. So how do we quit?* : Profile Books.
12. Berners-Lee, M., Howard, D.C., Moss, J., Kaivanto, K., and Scott, W., 2011. *Greenhouse gas footprinting for small businesses—The use of input–output data.* Science of the Total Environment, **409**(5): p. 883-891.
13. Börjesson Rivera, M., Håkansson, C., Svenfelt, Å. and Finnveden, G., 2014. Including second order effects in environmental assessments of ICT. Environmental Modelling & Software, 56, pp.105-115.
14. Cabernard, L., 2019. *Global supply chain analysis of material-related impacts in ICT (MRIO approach)*. http://www.lcaforum.ch/portals/0/df73/DF73-04_Cabernard.pdf - accessed March 2020.
15. Cabernard, L., Pfister, S., and Hellweg, S., 2019. *A new method for analyzing sustainability performance of global supply chains and its application to material resources.* Science of the Total Environment, **684**: p. 164-177.
16. Cambridge Dictionary, 2020. Available from: https://dictionary.cambridge.org/ - accessed March 2020.

17. Cisco, 2020. *Cisco Annual Internet Report (2018–2023).* https://www.cisco.com/c/en/us/solutions/collateral/executive-perspectives/annual-internet-report/white-paper-c11-741490.pdf - accessed February 2020.
18. CoinBundle Team, 2018. Consensus Algorithms. Securing Blockchain Transactions. Medium. https://medium.com/coinbundle/consensus-algorithms-dfa4f355259d#a76e - accessed March 2020.
19. Coroama, V.C., Hilty, L.M., Heiri, E., and Horn, F.M., 2013. *The direct energy demand of internet data flows.* Journal of Industrial Ecology, **17**(5): p. 680-688.
20. Coroama, V.C., Moberg, Å., and Hilty, L.M., 2015. *Dematerialization through electronic media?*, in *ICT Innovations for Sustainability*. Springer. p. 405-421.
21. Court, V., and Sorrell, S., 2020. *Digitalisation of goods: a systematic review of the determinants and magnitude of the impacts on energy consumption.* Environmental Research Letters, **15**(4): p.043001.
22. Department for Business, Energy and Industrial Strategy, 2019. *Greenhouse gas reporting: conversion factors 2019.* UK Government. https://www.gov.uk/government/publications/greenhouse-gas-reporting-conversion-factors-2019 - accessed March 2020.
23. Ercan, M., Malmodin, J., Bergmark, P., Kimfalk, E., and Nilsson, E., 2016. *Life cycle assessment of a smartphone*. in *ICT for Sustainability 2016*. Atlantis Press.
24. Ericsson, 2019. *Exponential data growth – constant ICT footprints.* https://www.ericsson.com/en/reports-and-papers/research-papers/the-future-carbon-footprint-of-the-ict-and-em-sectors - accessed March 2020.
25. ETIP SNET, *Integrating Smart Networks for the Energy Transition: Serving Society and Protecting the Environment.* Vision 2050. https://www.etip-snet.eu/etip-snet-vision-2050/ - accessed March 2020.
26. EU Blockchain, 2020. *Observatory and Forum*. Available from: https://www.euBlockchainforum.eu/ - accessed March 2020.
27. European Commission, 2018a. *European countries join Blockchain Partnership*. Shaping Europe's digital future. Digibyte. https://ec.europa.eu/digital-single-market/en/news/european-countries-join-Blockchain-partnership - accessed March 2020.
28. European Commission, 2018b. *Digital Innovation Hubs.* https://ec.europa.eu/futurium/en/system/files/ged/digital_innovation_hubs_in_digital_europe_programme_final2_december.pdf - accessed March 2020.
29. European Commission, 2019a. *The Internet of Things.* Shaping Europe's digital future. Policy. https://ec.europa.eu/digital-single-market/en/internet-of-things - accessed March 2020.
30. European Commission, 2019b. *Blockchain Unleashed for Climate Action*. Shaping Europe's digital future. Event, Trade Fair and Congress Center of Malaga. https://ec.europa.eu/digital-single-market/en/news/Blockchain-unleashed-climate-action - accessed March 2020.
31. European Commission, 2019c. *On Artificial Intelligence - A European approach to excellence and trust.* https://ec.europa.eu/info/sites/info/files/commission-white-paper-artificial-intelligence-feb2020_en.pdf - accessed March 2020.
32. European Commission, 2019d. *Policy and Investment Recommendations for Trustworthy AI.* Independent High-Level Expert Group on Artificial Intelligence set up by the European Commission. https://www.europarl.europa.eu/cmsdata/196378/AI%20HLEG_Policy%20and

%20Investment%20Recommendations.pdf - accessed March 2020.
33. European Commission, 2020. *Blockchain Technologies*. Shaping Europe's digital future. Policy. https://ec.europa.eu/digital-single-market/en/Blockchain-technologies - accessed March 2020.
34. FCAI, 2020. *The European Commission offers significant support to Europe's AI excellence*. https://fcai.fi/news/the-european-commission-offers-significant-support-to-europes-ai-excellence - accessed March 2020.
35. Fryer, E., 2020. *Does streaming really have a dirty secret?* TechUK. https://www.techuk.org/insights/opinions/item/17020-does-streaming-really-have-a-dirty-secret - accessed March 2020.
36. Galvin, R., 2015. *The ICT/electronics question: Structural change and the rebound effect.* Ecological Economics, **120**: p. 23-31.
37. Gartner, G.I. and Green, I., 2007. *The new industry shockwave, presentation at symposium*. in *ITXPO conference, April*.
38. GeSI, 2008. *Smart 2020: Enabling the low carbon economy in the information age.* A report by The Climate Group on behalf of the Global eSustainability Initiative. https://gesi.org/public/research/smart-2020-enabling-the-low-carbon-economy-in-the-information-age - accessed March 2020.
39. GeSI, 2012. *Smarter 2020: The Role of ICT in Driving a Sustainable Future.* A report by Boston Consulting Group on behalf of the Global eSustainability Initiative. https://gesi.org/report/detail/gesi-smarter2020-the-role-of-ict-in-driving-a-sustainable-future - accessed March 2020.
40. GeSI, 2015. *Smarter 2030: ICT Solutions for 21st Century Challenges.* A report by Accenture Strategy on behalf of the Global eSustainability Initiative. http://smarter2030.gesi.org/downloads.php - accessed March 2020.
41. Gilmore, M., 2018. *Final Report.* Expert and Stakeholder Consultation Workshop. Green ICT - Research and innovation activities (2020-2030). European Commission. https://ec.europa.eu/newsroom/dae/document.cfm?doc_id=50342 - accessed March 2020.
42. Gossart, C., 2015. *Rebound effects and ICT: a review of the literature*, in *ICT innovations for sustainability*. Springer. p. 435-448.
43. GSMA, The Enablement Effect, Technical Report, 2019. https://www.gsma.com/betterfuture/enablement-effect, accessed March 2020.
44. Hewlett, O., 2017. *Ensuring Renewable Electricity Market Instruments Contribute to the Global Low-Carbon Transition and Sustainable Development Goals.* Gold Standard. https://www.goldstandard.org/sites/default/files/documents/gs_recs_position_paper.pdf - accessed March 2020.
45. IEA, 2019. *Global Energy & CO2 Status Report.* https://www.iea.org/geco/emissions/ - accessed March 2020.
46. IPCC, 2018. *Special Report – Global Warming of 1.5 ºC.* https://www.ipcc.ch/sr15/ - accessed March 2020.
47. Jevons, W.S., 1865. *The coal question: Can Britain survive?* In: Flux, A.W. (Ed.), The Coal Question: An Inquiry Concerning the Progress of the Nation, and the Probable Exhaustion of our Coal-Mines. Augustus M. Kelley, New York
48. Kamiya, G., 2020. *Factcheck: What is the carbon footprint of streaming video on Netflix?* Carbon Brief. https://www.carbonbrief.org/factcheck-what-is-the-

carbon-footprint-of-streaming-video-on-netflix - accessed March 2020.
49. Kayali, L., Heikkilä, M., and Delcker, J., 2020. *Europe's digital vision, explained.* Politico. https://www.politico.eu/article/europes-digital-vision-explained/ - accessed March 2020.
50. Kennelly, C., Berners-Lee, M., and Hewitt, C., 2019. *Hybrid life-cycle assessment for robust, best-practice carbon accounting.* Journal of cleaner production, **208**: p. 35-43.
51. Lannoo, B., Lambert, S., Van Heddeghem, W., Pickavet, M., Kuipers, F., Koutitas, G., Niavis, H., Satsiou, A., Till, M., and Beck, A.F., 2013. *Overview of ICT energy consumption.* Network of Excellence in Internet Science: p. 1-59.
52. Lopez Yse, D., 2019. Your Guide to Natural Language Processing (NLP). Towards Data Science, Medium. https://towardsdatascience.com/your-guide-to-natural-language-processing-nlp-48ea2511f6e1 - accessed March 2020.
53. Lord, C., Hazas, M., Clear, A.K., Bates, O., Whittam, R., Morley, J. and Friday, A., 2015, April. Demand in my pocket: mobile devices and the data connectivity marshalled in support of everyday practice. *In Proceedings of the 33rd Annual ACM Conference on Human Factors in Computing Systems* (pp. 2729-2738).
54. Lövehagen, N., 2020. *What's the real climate impact of digital technology?* . Ericsson. https://www.ericsson.com/en/blog/2020/2/climate-impact-of-digital-technology - accessed March 2020.
55. Magee, C.L. and Devezas, T.C., 2017. *A simple extension of dematerialization theory: Incorporation of technical progress and the rebound effect.* Technological Forecasting and Social Change, **117**: p. 196-205.
56. Malmodin, J., 2020. The ICT sector's carbon footprint. Presentation at the techUK conference in London Tech Week on 'Decarbonising Data', 2020. https://spark.adobe.com/page/dey6WTCZ5JKPu/ - accessed December 2020.
57. Malmodin, J., 2019. *Energy consumption and carbon emissions of the ICT sector.* Presentation given at Energimyndigheten, Stockholm, 19 Dec 2019.
58. Malmodin, J., Bergmark, P., and Lundén, D., 2013. *The future carbon footprint of the ICT and E&M sectors.* on Information and Communication Technologies: p. 12.
59. Malmodin, J. and Lundén, D., 2018a. *The energy and carbon footprint of the global ICT and E&M sectors 2010–2015.* Sustainability, **10**(9): p. 3027.
60. Malmodin, J. and Lundén, D., 2018b. *The electricity consumption and operational carbon emissions of ICT network operators 2010-2015*. Report from the KTH Centre for Sustainable Communications Stockholm, Sweden.
61. Malmodin, J., Moberg, Å., Lundén, D., Finnveden, G., and Lövehagen, N., 2010. *Greenhouse gas emissions and operational electricity use in the ICT and entertainment & media sectors.* Journal of Industrial Ecology, **14**(5): p. 770-790.
62. Masanet, E., Shehabi, A., Lei, N., Smith, S., and Koomey, J., 2020. *Recalibrating global data center energy-use estimates.* Science, **367**(6481): p. 984-986.
63. Masanet, E., Shehabi, A., Lei, N., Vranken, H., Koomey, J., & Malmodin, J. (2019). Implausible projections overestimate near-term Bitcoin CO 2 emissions. Nature Climate Change, 9(9), 653-654.
64. McMahon, L., 2018. *Is staying online costing the earth?* Technical Report. Policy Connect. https://www.policyconnect.org.uk/appccg/research/staying-online-costing-earth - accessed March 2020.
65. NoCash, 2020. *European Commission about AI: „Europe needs to increase its*

investment levels significantly." Digital Innovation Hubs should provide support to SMEs to understand and adopt AI – at least one innovation hub per Member State. https://nocash.ro/european-commission-about-ai-europe-needs-to-increase-its-investment-levels-significantly-digital-innovation-hubs-should-provide-support-to-smes-to-understand-and-adopt-ai-at-least-one-innovat/ - accessed March 2020.

66. Olivier, J.G.J. and Peters, J.A.H.W., 2019. *Trends in global CO2 and total greenhouse gas emissions: 2019 report.* PBL Netherlands Environmental Assessment Agency, The Hague; 4004. https://www.pbl.nl/sites/default/files/downloads/pbl-2019-trends-in-global-co2-and-total-greenhouse-gas-emissions-summary-ot-the-2019-report_4004.pdf - accessed March 2020.
67. Pineda, A.C. and Faria, P., 2019. *Towards a science-based approach to climate neutrality in the corporate sector.* Science Based Targets report. https://sciencebasedtargets.org/wp-content/uploads/2019/10/Towards-a-science-based-approach-to-climate-neutrality-in-the-corporate-sector-Draft-for-comments.pdf - accessed March 2020.
68. Preist, C., Schien, D., and Blevis, E., 2016. *Understanding and mitigating the effects of device and cloud service design decisions on the environmental footprint of digital infrastructure*. in *Proceedings of the 2016 CHI Conference on Human Factors in Computing Systems*.
69. Preist, C., Schien, D., and Shabajee, P., 2019. *Evaluating Sustainable Interaction Design of Digital Services: The Case of YouTube*. in *Proceedings of the 2019 CHI Conference on Human Factors in Computing Systems*.
70. Sandvine, 2019. *Global Internet Phenomena Report.* https://www.sandvine.com/hubfs/Sandvine_Redesign_2019/Downloads/Internet%20Phenomena/Internet%20Phenomena%20Report%20Q32019%2020190910.pdf - accessed February 2020.
71. Schaffartzik, A., Mayer, A., Gingrich, S., Eisenmenger, N., Loy, C., and Krausmann, F., 2014. *The global metabolic transition: Regional patterns and trends of global material flows, 1950–2010.* Global Environmental Change, **26**: p. 87-97.
72. SBTi (Science Based Targets), 2019. *Science-Based Target Setting Manual.* https://sciencebasedtargets.org/wp-content/uploads/2017/04/SBTi-manual.pdf - accessed March 2020.
73. SBTi (Science Based Targets), 2020. *SBTi Criteria and Recommendations.* https://sciencebasedtargets.org/wp-content/uploads/2019/03/SBTi-criteria.pdf - accessed July 2020.
74. Scott, M., 2019. *New rules to crack down on 'greenwash' in corporate clean energy claims.* Ethical Corporation. http://www.ethicalcorp.com/new-rules-crack-down-greenwash-corporate-clean-energy-claims - accessed March 2020.
75. Shehabi, A., Walker, B., and Masanet, E., 2014. *The energy and greenhouse-gas implications of internet video streaming in the United States.* Environmental Research Letters, **9**(5): p. 054007.
76. Sky, 2020. *Sky Zero.*; Available from: https://www.skygroup.sky/sky-zero - accessed March 2020.
77. Smith, B., 2020. *Microsoft will be carbon negative by 2030.* Microsoft. https://blogs.microsoft.com/blog/2020/01/16/microsoft-will-be-carbon-negative-by-2030/ - accessed March 2020.

78. Sorrell, S., 2009. *Jevons' Paradox revisited: The evidence for backfire from improved energy efficiency.* Energy policy, **37**(4): p. 1456-1469.
79. Sorrell, S., Gatersleben, B. and Druckman, A., 2020. The limits of energy sufficiency: A review of the evidence for rebound effects and negative spillovers from behavioural change. *Energy Research & Social Science*, *64*, p.101439.
80. Stephens, A., 2019. *Net zero: an ambition in need of a definition*. Carbon Trust. https://www.carbontrust.com/news-and-events/insights/net-zero-an-ambition-in-need-of-a-definition - accessed March 2020.
81. Sweney, M., 2020. *Netflix to slow Europe transmissions to avoid broadband overload.* The Guardian. https://www.theguardian.com/media/2020/mar/19/netflix-to-slow-europe-transmissions-to-avoid-broadband-overload - accessed March 2020.
82. The Shift Project, 2019a. *Climate Crisis: The Unsustainable Use of Online Video. The practical case for digital sobriety.* https://theshiftproject.org/en/article/unsustainable-use-online-video/ - accessed March 2020.
83. The Shift Project, 2019b. *Lean ICT: Towards digital sobriety.* https://theshiftproject.org/en/lean-ict-2/ - accessed March 2020.
84. UK Energy Research Council, 2007. *The Rebound Effect: An Assessment of the evidence for economy-wide energy savings from improved energy efficiency.* http://www.ukerc.ac.uk/programmes/technology-and-policy-assessment/the-rebound-effect-report.html - accessed March 2020
85. Van Heddeghem, W., Lambert, S., Lannoo, B., Colle, D., Pickavet, M., and Demeester, P., 2014. *Trends in worldwide ICT electricity consumption from 2007 to 2012.* Computer Communications, **50**: p. 64-76.
86. Vaughan, A., 2019. *Cloud gaming may be great for gamers but bad for energy consumption.* New Scientist. https://institutions.newscientist.com/article/2206200-cloud-gaming-may-be-great-for-gamers-but-bad-for-energy-consumption/ - accessed March 2020.
87. Vereecken, W., Deboosere, L., Simoens, P., Vermeulen, B., Colle, D., Develder, C., Pickavet, M., Dhoedt, B., and Demeester, P., 2009. *Energy efficiency in thin client solutions*. in *International Conference on Networks for Grid Applications*. Springer.
88. Walnum, H.J. and Andrae, A.S., 2016. *The internet: Explaining ICT service demand in light of cloud computing technologies*, in *Rethinking Climate and Energy Policies*. Springer. p. 227-241.
89. Widdicks, K., Hazas, M., Bates, O., and Friday, A., 2019. *Streaming, Multi-Screens and YouTube: The New (Unsustainable) Ways of Watching in the Home*. in *Proceedings of the 2019 CHI Conference on Human Factors in Computing Systems*.
90. Wiebe, K.S., Bjelle, E.L., Többen, J., and Wood, R., 2018. *Implementing exogenous scenarios in a global MRIO model for the estimation of future environmental footprints.* Journal of Economic Structures, **7**(1): p. 20.
