# Supplementary material for: The real climate and transformative impact of ICT: A critique of estimates, trends, and regulations
Source: Patterns (N Y). 2021 Sep 10;2(9):100340. doi: 10.1016/j.patter.2021.100340 (PMC8441580; doi:10.1016/j.patter.2021.100340)
Supplement: Document S2. Article plus supplemental information [file mmc2.pdf]

Patterns

CellPress
OPEN ACCESS

Review

The real climate and transformative impact of ICT: A critique of estimates, trends, and regulations

Charlotte Freitag,[1] Mike Berners-Lee,[1] Kelly Widdicks,[2,*] Bran Knowles,[2] Gordon S. Blair,[2] and Adrian Friday[2]
[1]Small World Consulting, Gordon Manley Building, Lancaster Environment Centre, Lancaster University, Lancaster, Lancashire LA1 4YQ, UK
[2]School of Computing and Communications, InfoLab21, Lancaster University, Lancaster, Lancashire LA1 4WA, UK
*Correspondence: k.v.widdicks@lancaster.ac.uk
https://doi.org/10.1016/j.patter.2021.100340

THE BIGGER PICTURE To avoid catastrophic consequences from climate change, all sectors of the global economy, including *Information Communication Technology* (*ICT*), must keep their greenhouse gas (GHG) emissions in line with the Paris Agreement. We examine peer-reviewed estimates of ICT's GHG emissions, which put ICT's share of global GHG emissions at 1.8%–2.8%. We find pronounced differences and much debate concerning the underlying assumptions behind the peer-reviewed studies, which could suggest that global emissions from ICT are as high as 2.1%–3.9%. All study analysts agree that ICT emissions *will not reduce* without major concerted political and industrial efforts, and we provide three reasons for anticipating that ICT emissions are actually going to *increase* without intervention. Our analysis suggests not all ICT carbon pledges are ambitious enough to meet climate targets, and that policy mechanisms for enforcing sector-wide climate target compliance are lacking. Without a global carbon constraint, sector-wide regulations are required to keep ICT's carbon footprint aligned with the Paris Agreement. With a global carbon constraint, ICT would be a greater enabler of productivity and utility, creating opportunity for the sector to be financially successful as a critical part of a global net zero society.

SUMMARY

In this paper, we critique ICT's current and projected climate impacts. Peer-reviewed studies estimate ICT's current share of global greenhouse gas (GHG) emissions at 1.8%–2.8% of global GHG emissions; adjusting for truncation of supply chain pathways, we find that this share could actually be between 2.1% and 3.9%. For ICT's future emissions, we explore assumptions underlying analysts' projections to understand the reasons for their variability. All analysts agree that ICT emissions will not reduce without major concerted efforts involving broad political and industrial action. We provide three reasons to believe ICT emissions are going to increase barring intervention and find that not all carbon pledges in the ICT sector are ambitious enough to meet climate targets. We explore the underdevelopment of policy mechanisms for enforcing sector-wide compliance, and contend that, without a global carbon constraint, a new regulatory framework is required to keep the ICT sector's footprint aligned with the Paris Agreement.

INTRODUCTION

The Information and Communication Technology (ICT) sector has seen massive and accelerating growth in the last 70 years. ICT is now so significant that there is an increasing awareness of the potential environmental effects of ICT, particularly on climate change. ICT has a growing "carbon footprint" arising from greenhouse gases (GHG) released from all its life cycle stages. This includes embodied emissions (the GHG emissions released from the extraction of raw materials required, the manufacturing process and transport to the business or user), use or operational emissions (from energy use and maintenance) and end-of-life emissions (disposal). Yet estimates of ICT's footprint and whether it is in fact growing in impact, or held stable or even reducing by efficiency gains and Moore's Law, is very much a topic of lively debate. Many increasingly point also to ICT's potential to decarbonize other sectors. It is argued that this "enablement" is a key ingredient in the pathway to carbon neutrality, and in many ways exempts or justifies the footprint of ICT itself.

In this paper we look at accepted estimates of climate change impacts of ICT now and in the future (Estimating the carbon footprint of ICT) and ask critical questions concerning efficiency:

This is an open access article under the CC BY license (http://creativecommons.org/licenses/by/4.0/).

whether efficiency gains could reduce emissions in the ICT sector and global economy over time, or whether these are more than offset by possible "rebound effects." In this context, we take a broad view of rebound effects to include any increases in emissions due to the introduction of ICT or the efficiencies it enables, and include an example of a rebound effect (Jevons Paradox) in our supplemental information (this supplemental information includes an appendix for this paper, which goes into more depth about our literature review method, analysis, and additional information relevant to this work—specifically: the methodology, estimates of ICT emissions, video streaming, narratives (Six common narratives for ICT's role in climate change), truncation error, the European Commissions' investment in ICT, carbon pledges, renewable energy purchases, and Jevons Paradox). In this paper we also explore the importance of emerging trends in ICT (big data, data science, and artificial intelligence [AI]; the Internet of Things [IoT]; and blockchain) that could provide opportunities for environmental sustainability yet threaten global emissions reduction (ICT Trends: Opportunities and threats), as well as suggest important areas of regulation and governance (Current policy developments and governance in ICT).

Given the topic importance, there are surprisingly few studies analyzing the environmental impact of ICT and they are often characterized by a lack of interrogatability, potential for conflict of interest, a limited scope that leaves out growing ICT trends and an underestimation of ICT's carbon footprint as significant proportions of total emissions are omitted. We draw on peer-reviewed journal articles published from 2015 on the topic (Estimating the carbon footprint of ICT), and analyze trends in ICT and their environmental implications (ICT Trends: Opportunities and threats). For this, we include literature on the energy or carbon impacts of ICT, its major components (e.g., data centers, networks), its major application areas (e.g., AI, IoT), and the impact ICT has on energy or carbon consumption in other sectors. We go also beyond the literature: including consultations with the lead authors of the main studies who are included in this review, as well as other experts, to better assess ICT emission estimations and the associated complexities; and drawing upon research by Small World Consulting (SWC) to account for emissions omitted in many assessment methodologies (see our supplemental information for further details on the model used for assessment). For our policy analysis (European policy and ICT), we focus on European Commission documents and websites, supplemented by an analysis of industry pledges (Self-regulation in the ICT industry) drawn from analysis of annual reports, blog posts, and web pages from major ICT companies.

While there are limitations to our study in review scope and the uncertainties of carbon calculations, we are confident we have captured the main debates, and contribute through our focus on GHG emissions. We specifically focus on GHG emissions rather than electricity consumption as the former drives climate change and the latter does not capture important factors surrounding ICT's environmental impact. Through our analysis, we have found broad agreement on the size of ICT's current carbon footprint, yet there are a range of different views with regard to ICT's future role in climate change, both in terms of ICT's own carbon footprint and its effect on the wider economy's emissions—we discuss the arguments and assumptions underpinning these different views and their policy implications. Nevertheless, analysts included in our investigations agree that ICT emissions will not reduce without major concerted efforts involving broad political and industrial action, and we provide three reasons that indicate ICT emissions are actually going to increase without intervention. It is clear from our study that too much reliance is placed on a switch to renewables, and efficiency gains within and beyond the ICT sector, for achieving carbon targets; significant action through a global constraint (e.g., a carbon cap on extraction), and more assessment of ICT's rebounds and governance are required.

Estimating the carbon footprint of ICT

In this section, we provide a broad overview of the estimates for ICT's carbon footprint before 2015, and an in-depth analysis of three major peer-reviewed studies of ICT's estimated emissions. We identify the key arguments and assumptions underpinning the different estimates, noting the essential points of agreement and crucially the major points for and against growth in ICT sectors emissions into the future.

ICT's carbon footprint

Historically, ICT emissions have grown continuously alongside global emissions. Several studies prior to 2015 have estimated the carbon footprint of ICT (summarized in Figure 1). These show an increase in ICT's carbon footprint over time, even without considering the full life cycle emissions, with the trend line showing a 40% increase 2002–2012. The growth in ICT's emissions has coincided with consistent growth in our total global carbon footprint,[1] where global GHGs have grown by 1.8% per year[2] (approximately 20% per decade). This indicates ICT's footprint has likely grown faster than global emissions, with a very uncertain best estimate of twice as fast. Going back in time further, ICT's footprint will have grown faster than global emissions since the sector started from zero mid-last century.

Scientific debate over ICT's emissions has intensified in the last 5 years. We therefore focus on research since 2015—especially studies by three main research groups led by Andrae,[3–6] Belkhir,[7] and Malmodin.[8,9] Andrae and Edler[3] estimate ICT's emissions for every year 2010–2030, Belkhir and Elmeligi[7] for 2007–2040 and Malmodin and Lundén[8,9] for 2015. Malmodin has also provided additional estimates for 2020 to us in personal communication. We summarize the arguments in this section.

ICT's current carbon footprint

ICT is estimated at ca. 1.8%–2.8% of global GHG emissions in 2020. Estimates of ICT's emissions in 2020 (see Figure 2) vary between 0.8 and 2.3 $GtCO_2e$. The highest estimates (Andrae and Edler[3] "worst case") put ICT's share of global GHG emissions around 6.3%, but Andrae now believes that the Andrae and Edler[3] "best case" scenario of around 1.5% is more realistic for 2020 (personal communication). Belkhir and Elmeligi[7] estimates are higher at 1.9%–2.3%, especially considering they omit TVs in their total estimate. Malmodin's estimates sit in between the others at 1.9% of global emissions. When adjusting for differences in scope, these studies point toward a footprint of 1.0–1.7 $GtCO_2e$ for ICT, TVs, and other consumer electronics in 2020; this is 1.8%–2.8% of global GHG emissions. We stress that this estimate carries some uncertainty but gives us a reasonable idea of the impact of ICT. Across studies, roughly 23% of ICT's total footprint is from embodied emissions, yet the share of embodied emissions for user devices specifically is ca.

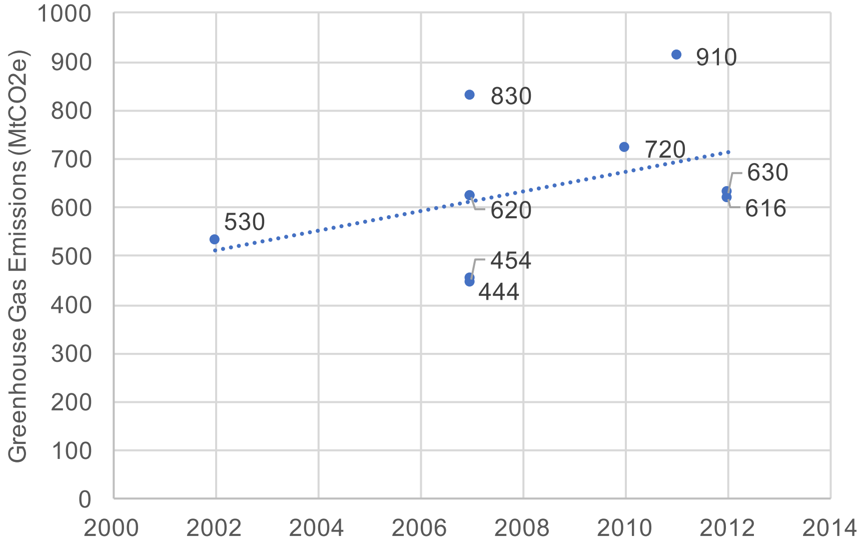

Figure 1. Estimates of ICT's carbon footprint from studies published before 2015
The linear best fit line shows the increase in emissions with time, although the growth is not necessarily linear.

50%. This is because, unlike networks and data centers, user devices are only used for parts of the day and use less electricity, but are exchanged often, especially in the case of smartphones. Electricity consumption of user devices and domestic equipment has decreased over the last 15–20 years driven by legislation and public procurement policy, such as the EU ERP directive and EnergyStar (Chris Preist, personal communication). However, efficiency improvements will not reduce embodied emissions drastically. While production processes are becoming more efficient, the manufacturing footprint of smartphones is increasing because of more advanced integrated circuits, displays, and cameras (Malmodin, personal communication). With a large share of their footprint coming from their manufacture, extending smartphones' lifetime is the best way to reduce their footprint. Most studies reviewed here assume an average lifetime of 2 years, partly driven by phone contracts that promise users the newest models.[7] There are some signs, though, that this might be increasing slightly. For example, the NPD[10] reported that in the US, the average use has increased to 32 months in 2017 up from 25 months in 2016. Legislation encouraging repair, e.g., the EU Waste Electrical and Electronic Equipment Directive, can help, alongside business models centering around service rather than product provision or selling repairable products to markets in the Global South (Preist, personal communication).

There are important differences in how analysts arrived at these estimations. There is a lack of agreement about which technologies ought to be included in calculations of ICT's GHG emissions—particularly TV. All studies include data centers, networks, and user devices as the three main components of ICT, but there are pronounced differences of opinion regarding the proportional impact of each. A comparison of the different proportions in 2020 estimates (excluding TV) is provided below (Figure 3).

Regarding data centers, Belkhir himself noted that his projection of 495 $MtCO_2e$ for data centers in 2020 is overestimated (personal communication). Recent evidence by Masanet et al.[11] of 205 TWh total energy use in 2018 seems to converge with Malmodin's estimate of 127 $MtCO_2e$ in 2020. Assuming a global electricity mix at 0.63 $kgCO_2e$/kWh, Masanet et al.'s[11] estimate comes to ca. 129 $MtCO_2e$ —higher than Andrae and Edler's[3] best case estimate of 217 $MtCO_2e$.

Studies systematically underestimate the carbon footprint of ICT due to the "truncation error." This error arises from the partial exclusion of supply chain pathways by the traditional process of life cycle analysis (LCA). Malmodin's studies are the most comprehensive as they include operator activities and overheads (e.g., offices and vehicles used by data center and network operators), as well as considering life cycle emissions of equipment (i.e., from production, use, to disposal) rather than just production energy[3] or only material extraction and manufacturing energy.[7] However, Andrae and Edler,[3] Belkhir and Elmeligi,[7] and Malmodin and Lundén[8,9] all follow LCA methodologies, which are unable to include the infinite number of supply chain pathways of a product, thereby incurring "truncation error" in their carbon accounting. Similarly, but of less significance, they also do not consider the full supply chain carbon footprint of electricity used to run ICT equipment. However, in the assessment of emissions from products, including electricity, the system boundary can be expanded to include all supply chain pathways by combining traditional LCA with environmentally extended input output (EEIO) methodologies. By mapping the LCA's system boundary onto the EEIO model, an EEIO-based estimate can be made of the truncated supply chain pathways. When truncation error has been adjusted for in this way, the carbon footprint for ICT, including TVs and other consumer electronics, rises to 1.2–2.2 $GtCO_2e$ (2.1%–3.9% of global GHG emissions) in 2020 with ca. 30% coming from embodied emissions and 70% from use phase emissions. We stress once more that these are rough estimates with a significant degree of uncertainty.

ICT's future carbon footprint

There is broad agreement by analysts in the field on certain key assumptions:.

- the world's carbon footprint needs to decrease to avoid climate catastrophe
- data traffic is continuing to grow
- energy demand by ICT is increasing
- demand for data centers and network services will increase
- the shift to smartphones is decreasing emissions from PCs and TVs
- using more renewable energy would reduce ICT emissions
- ICT could reduce emissions in other sectors but not by default and only under certain conditions (contrasting to GeSI[12] SMARTer, 2030 claims)
- ICT has the potential to increase its own emissions and facilitate rising emissions in other sectors

Opinions are more divided regarding future trends in emissions. From 2015 to 2020, Belkhir and Elmeligi's[7] and Andrae and Edler's[3]

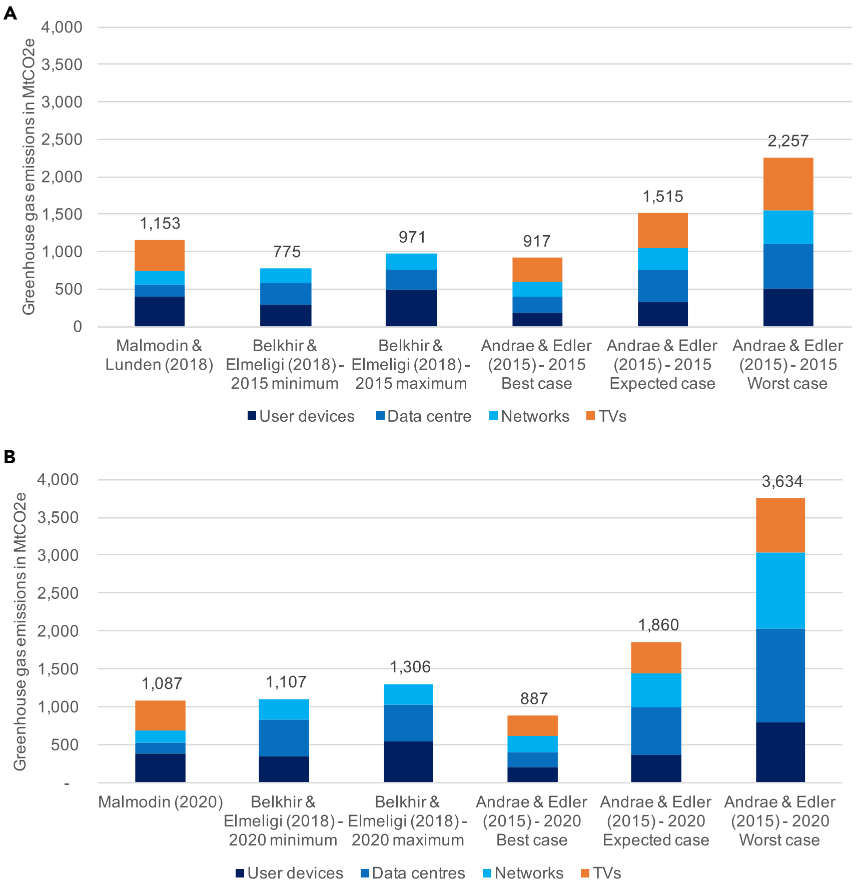

Figure 2. Estimates for global ICT's carbon footprint in 2015 and 2020

(A) Estimates for global ICT's carbon footprint in 2015.
(B) Estimates for global ICT's carbon footprint in 2020. Note that for Malmodin and Lundén's[8,9] estimates, TV includes TV networks and other consumer electronics, whereas for Andrae and Edler's[3] estimates, only TVs themselves and TV peripherals are included. Belkhir and Elmeligi[7] did not include TVs. Malmodin and Lundén's[8,9] original estimates for the ICT and entertainment and media sector includes paper media, which we have excluded here.

estimates of ICT emissions have increased due to an increase in data traffic and the number of user devices (see Figure 2). In contrast, Malmodin's estimates have decreased slightly—mostly for data centers (by 10%) due to an increased adoption of renewable energy, and for networks (by 8%) due to decreases in overheads, despite increases in their electricity consumption.

Malmodin (personal communication) argues that: GHG emissions from ICT have stabilized for now; ICT and the entertainment and media sector growth is starting to decouple from GHG emissions; and that ICT could even halve its 2020 emissions by 2030 through renewable energy transformation and collective effort[13] to 365 $MtCO_2e$ in 2030.[14] In contrast, Belkhir and Elmeligi[7] and Andre and Edler[3] believe that emissions from ICT will continue to grow (see Figure 4).

All analysts think that it would be possible in theory for ICT to decrease its emissions with broad political and industry action—but Malmodin is more optimistic that this will happen than Belkhir and Elmeligi[7] and Andrae and Edler.[3] A recent report by Ericsson[15] based on Malmodin and Lundén[8,9] claims that ICT's emissions could be reduced by 80% if all its electricity came from renewable sources.

Differences in predictions could be due to age of data used. The data underlying Andrae and Edler's[3] and Belkhir and Elmeligi's[7] work is somewhat older (Andrae and Edler[3] use some data from 2011 for data centers and networks, while Belkhir and Elmeligi[7] use data from 2008 for data centers and from 2008 to 2012 for networks) considering ICT's fast pace of development, meaning their projections are potentially based on historical trends that might no longer apply, such as the assumed exponential growth of energy consumption by data centers and networks. In contrast, Malmodin and Lundén[8,9] might better capture recent changes in emission trends given their estimates are based on data measured directly from industry (Malmodin and Lundén's[8,9] estimates are based on 2015 data; Malmodin's more recent estimates provided in personal communication are based on data from 2018 onward). Malmodin and Lundén[8,9] also have the most inclusive scope in terms of ICT equipment, life cycle stages and supply chain emissions considered.

However, this access to industry data inevitably comes at the price of a lack of data interrogatability. Part of Malmodin's data were obtained by ICT companies under confidentiality agreements, preventing others from reviewing the original data and the model's assumptions and calculations. There are also potential risks of conflicts of interest as both authors work for network operators (Malmodin works for Ericsson, Lundén works for Talia). This arguably makes the Malmodin and Lundén[8,9] paper open to concerns that claims are less reliable due to selective reporting and assumptions that cannot be properly assessed. We are not suggesting that they cannot be trusted, but the lack of transparency makes independent data and analysis difficult, and transparency is necessary for important policy decisions. As employees of Huawei, Andrae and Edler[3] also have potential for conflict of interest, but their study is transparent about their data sources, calculations, and assumptions. Belkhir and Elmeligi[7] have no obvious conflict of interest and they use only peer-reviewed and publicly available sources.

Due to the trade-off between data interrogatability and up-to-date data, it is impossible to judge which study makes the most reliable predictions about ICT's future emissions based on methodology alone. It is possible, however, to examine their arguments and the underlying assumptions to assess which projection is more likely.

ICT's future carbon footprint: unpacking the studies' assumptions

In the key studies reviewed here, there is disagreement on whether or not:

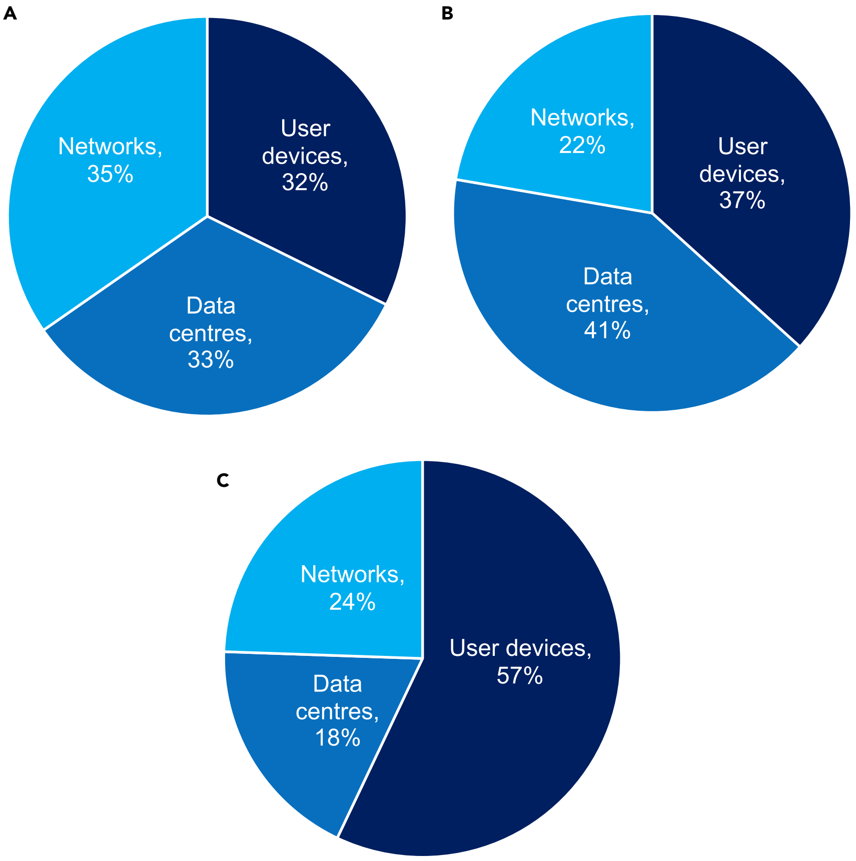

Figure 3. Proportional breakdown of ICT's carbon footprint, excluding TV

(A) Andrae and Edler (2015): 2020 best case (total of 623 $MtCO_2e$).
(B) Belkhir and Elmeligi (2018): 2020 average (total of 1,207 $MtCO_2e$).
(C). Malmodin (2020): 2020 estimate (total of 690 $MtCO_2e$).
Andrae and Edler's[3] best case is displayed because more recent analysis by the lead author suggest that this scenario is most realistic for 2020. Note that Malmodin's estimate of the share of user devices is highest; this is mostly because Malmodin's network and data center estimates are lower than those of the other studies.

- energy efficiencies in ICT are continuing
- energy efficiencies in ICT are reducing ICT's carbon footprint
- ICT's carbon footprint will stabilize due to saturation in ICT
- data traffic is independent of ICT emissions
- ICT will enable emissions savings in other industries
- renewable energy will decarbonize ICT

These assumptions have a critical influence on what we can conclude about ICT's role in climate change. We therefore explore the arguments on both sides of the debate to shed some light on the most likely path of ICT's future emissions. In doing so, we draw on several other much-cited sources and direct consultation with key experts.

Are energy efficiency improvements in ICT continuing?

There has been a long history of ICT equipment becoming more efficient (and thus cheaper and more productive) with time. Moore's Law allowed the ICT industry to exponentially increase chips' performance, speed, and reduce their power consumption. The exponential improvements of processors has kept the exponential growth in demand partly in check in terms of energy consumption.

While Malmodin and Lundén[8,9] acknowledge that Moore's Law has slowed down since 2012, they note that there is usually a time lag before the effects are felt outside of research labs—therefore arguing that efficiencies are continuing for now. Masanet et al.[11] argue that there is scope for further efficiency improvements in data centers through: improvements in server virtualization; efficiency gains in servers, storage devices, and data center cooling technology; and the move toward large data centers that are more energy efficient due to efficiencies of scale and the ability to invest in AI to optimize energy use.

For efficiency improvements in user devices, there is evidence of carbon savings from TVs: older, more energy-intensive CRT and plasma TVs have been replaced by more efficient LED TVs; and TV sales have dropped due to users now watching video on laptops and smartphones (Belkhir and Elmeligi,[7] Malmodin). However, smart TVs could change this trend if they become a popular way to access streamed media (Preist, personal communication).

However, efficiency improvements might be coming to an end—a view echoed by some of the experts we have consulted (e.g., Peter Garraghan, Belkhir, Andrae). As transistors have shrunk in size and increased in speed, they have begun to heat up; this led to manufacturers putting a speed limit on processing in 2004. The problem now is "quantum entanglement" where transistor layers become so thin that electrons jump between them, making transistors increasingly unreliable.[16] Other avenues may exist for improving efficiencies (e.g., decreasing semiconductor use stage power and nanophotonics),[17] but possibly not on the same timescales[18] or with the same efficiency gains.

If processor efficiencies are reaching a limit, data centers' power consumption will likely rise as increasing demand will no longer be counterbalanced by increasing efficiency. Despite some remaining scope for further efficiency improvements, Masanet et al.[11] note that there are limits to efficiency improvements and that energy demand will not stabilize by itself—arguing that urgent policy action and investment are needed to limit increases in energy use driven by increasing demand. Furthermore, efficiencies in ICT do not always guarantee replacement of the older, less efficient equipment (e.g., the development of 5G networks while 2G, 3G, and 4G networks still exist) and new devices or user habits may conflict with replacement gains. For example, some new ICT devices,

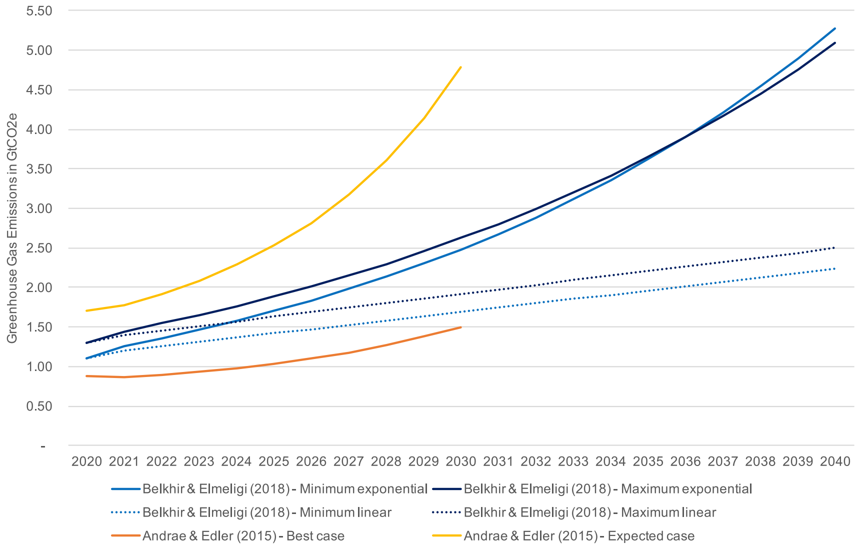

Figure 4. Projections of ICT's GHG emissions from 2020

(A) Andrae, (B) Belkhir, (C) Malmodin, personal communication. Belkhir and Elmeligi[7] judge their exponential scenario as most realistic, while the linear growth scenario is more conservative and reflects the impact of mitigating actions between now and 2040. Malmodin and Lundén[8,9] did not make concrete estimates beyond 2020, but Malmodin suggests that ICT's carbon footprint in 2020 could halve by 2030—offering a 2030 estimate of 365 $MtCO_2e$ in a recent techUK talk.[14]

such as smart watches and smart speakers, are used by people in addition to smartphones and laptops, and Court and Sorrell[19] also highlight the issue of incomplete substitution of e-materialization trends like e-news or e-books. Multiple user devices in the home have also led to a third of UK households watching separate video content simultaneously in the same room once a week[20] where people may have watched content using the same TV before.

Are energy efficiencies in ICT reducing ICT's carbon footprint?

Malmodin argues that so far, efficiency improvements are continuing, and data center emissions are expected to stay at 1% of global electricity and at the same level of emissions as in 2015 in the next 5 years. Furthermore, Masanet et al.[11] reported that data centers' operational energy consumption has increased only marginally from 194 TWh in 2010 to 205 TWh in 2020 despite global data center compute instances increasing by 550% over the same time period—showing the effectiveness of efficiency improvements in ICT. Masanet et al.[11] also note that these efficiency improvements would be able to offset a doubling of data center demand relative to 2018; beyond that point, energy demand will rise rapidly. This is in line with what Belkhir (personal communication) believes, although he is less optimistic about the remaining scope for efficiency improvements.

As highlighted above, ICT has seen rapid and continuous efficiency gains. Yet increases in demand for computation and the number of ICT-enabled devices per person have outpaced these energy efficiency improvements, resulting in growth in ICT's energy consumption and carbon footprint year-on-year. This pattern fits with the rebound effect described by Jevons Paradox whereby an efficiency improvement leads to an even greater proportionate increase in total demand, meaning total resource requirements rise rather than falls, as is often assumed. While Jevons Paradox has not been proved to apply within the ICT industry, it is risky to assume it does not apply given historical evidence of ICT emissions consistently rising despite significant improvements in efficiency (ICT's carbon footprint).

It would be surprising if rebound effects in ICT—and Jevons Paradox in particular—were to end in the future without a foundational change.[21] There is a theoretical alternative scenario (the reverse of Jevons Paradox) where stalled energy efficiency growth leads to a plateau in ICT emissions due to prohibitive costs as increasing demand cannot be counterbalanced by efficiency improvements any longer. There is little precedent for this in prior work.

Are ICT's emissions likely to stabilize due to saturation?

The studies reviewed here all agree that the number of smartphones is increasing. According to Cisco,[22] there will be 5.7 billion mobile subscribers by 2023–71% of the world population. However, within a few years, every person on earth might have a smartphone and the total number might not further increase (Malmodin, personal communication). There is some evidence suggesting that the average lifetime of smartphones is increasing too,[10] which will decrease the yearly embodied carbon associated with people replacing their smartphones. In addition, Malmodin argues that there is a limited time per day that people can be using their phones, theoretically capping energy consumption. The same pattern of saturation could be true for other ICT equipment, which could stabilize ICT's emissions.

However, ICT companies generally have a strong incentive to prevent saturation from happening as this would cut their income growth. There is economic pressure for them to create new technologies for individuals and organizations to buy. An example of this is the increase in IoT devices, which require little person time and can operate in the background, driving both embodied and use phase emissions from the production of billions of IoT devices, the networks allowing them to communicate and from data centers that analyze the IoT data (see The Internet of Things). Other important trends (ICT Trends: Opportunities and threats), such as the growth in AI, would also escape this natural saturation. The history of ICT does not provide precedents for a saturation effect; it is therefore unlikely to occur without active intervention. Furthermore, there is still scope for more ICT infrastructure growth beyond smartphones before this innovation cycle even begins, e.g., for data centers in the Global South (Preist, personal communication).

Is data traffic independent of ICT emissions?

The amount of data traffic on the internet at a given time does not correspond with simultaneous increases in ICT's emissions. Instead, network operators plan capacity for peak data traffic,[23]

meaning emissions from ICT are fixed regardless of the amount of data traffic until growth in peak capacity is required. In Malmodin and Lundén's[8,9] view, data traffic is not directly proportional to emissions due to efficiency gains and use of renewable energy in data centers and networks that allow them to process increasingly more data with similar emissions. Malmodin and Lundén[8,9] (reiterated by Ericsson)[15] believe the energy consumption of ICT is instead linked to the number of users and time spent using ICT because of the energy consumption of user devices and access equipment, such as modems and routers, and that data traffic growth is slowing down to a more linear than exponential growth.

Andrae and Edler[3] and Belkhir and Elmeligi[7] both agree that data traffic is a driver in ICT growth and emissions. Growth in the internet's infrastructure capacity allows for new data-intensive services and applications; these offer more affordances to users, driving demand for the services and therefore further infrastructure growth.[24] Peak data traffic is one driver for this infrastructure growth due to increased demand for data-intensive services; other influences include ensuring technology is always accessible to all users (Preist, personal communication).

Video streaming is a particularly prominent driver in data traffic. During the COVID-19 pandemic, Netflix agreed with EU regulators to reduce their traffic and ease the load on the network, allowing network provision for homeworkers.[25] Belkhir (personal communication) pointed out that this agreement between Netflix and EU regulators makes it difficult to argue that data traffic is independent of ICT infrastructure growth and therefore that data traffic has little effect on emissions.

Is ICT enabling carbon savings in other industries?

In their report SMARTer 2030, the Global eSustainability Initiative,[12] which represents ICT companies, claim that ICT could save 9.1 $GtCO_2e$ in 2020 and 12.08 $GtCO_2e$ in 2030 in other industries, such as health, education, buildings, agriculture, transport, and manufacturing—mostly due to improved efficiency. This would allow a 20% reduction of global CO_2e emissions by 2030, holding emissions at 2015 levels and decoupling economic growth from emissions growth. Relative to their estimate of ICTs own emissions of 1.27 $GtCO_2e$ in 2020 and 1.25 $GtCO_2e$ in 2030, GeSI[12] argue that ICT is net carbon negative and that governments and businesses should invest more into ICT. According to them, already in 2015, ICT saved 1.5 times its own emissions. There is also a strong argument that ICT will accelerate the use of renewable energy in the grid and hence lead to decarbonization of the energy supply.

The GeSI[12] report is sponsored by several large ICT companies and there is a lack of transparency in their analysis, raising concerns over possible conflict of interest. So far, there is little evidence that these predictions have come true. History has shown us that growth in the global economy and its carbon footprint has continuously risen, even with ICT creating efficiencies in other industries. It is risky to assume that further ICT-enabled efficiencies will suddenly start to create significant carbon savings in the wider economy without governance and intervention. Rather, it is more likely that ICT enables emission increases in other sectors because it enables efficiencies, leading to growth in the very areas into which ICT delivers those efficiency gains—including growth in industries that are already carbon-intensive (Preist, personal communication). By efficiencies here, it is important to note that we go beyond just energy-specific efficiencies as described by Jevons Paradox; rather, we take into account ICT's emission impacts and rebound effects more widely[cf.26] and refer to any potential route for rebound ICT brings to our society (e.g., consider how ICT has made it far easier to book flights online, contributing to the growth of the aviation industry).

While GeSI[12] mention rebound effects, this is only in the appendix and given very limited treatment. Their estimate of an increase of global emissions by 1.37 $GtCO_2e$ due to rebound effects is not included in overall calculations for emission savings by ICT and is almost certainly a serious underestimation. This is highlighted by their example of video conferencing[12] estimating that "E-Work technologies like videoconferencing could save around 3 billion liters of fuel." by cutting workers' commutes. It is difficult to quantify the exact balance of ICT-enabled savings and increased emissions, but one clue is that while video traffic has been expanding rapidly to the extent that it is one of the main contributors of internet traffic,[22] emissions from flights were simultaneously increasing (save for pandemics).[27] Therefore, ICT only enables efficiencies in other industries if it completely substitutes more traditional carbon-intensive activities rather than being offered in addition to them.

Will renewable energy decarbonize ICT?

While the exact share of renewable energy used for the ICT sector is not known, some ICT operators generate renewable energy on-site and the ICT sector overall is a major purchaser of renewable energy—leading the way for a global shift to this energy source. In a recent Ericsson blogpost building on Malmodin's work, Lövehagen[28] claims that ICT's carbon footprint could be reduced up to 80% if all electricity came from renewable energy. Renewable energy has a much lower carbon footprint than fossil fuel energy at ca. 0.1 $kgCO_2e/kWh$. Compared to 0.63 $kgCO_2e/kWh$ for the global electricity mix, a switch to 100% renewable energy would reduce emissions by ca. 86%. Both of these $kgCO_2e/kWh$ figures are based on SWC's EEIO model that draws on official data from the UK government's Department for Business, Energy and Industrial Strategy.

With unlimited growth in energy demand, even the relatively small carbon footprint from renewable energy compared to fossil fuel would add up significantly. In addition, there might be limits to the amount of renewable energy that can be generated with present technology, such as the availability of silver, which is used in photovoltaic panels. An average solar panel requires ca. 20 g of silver[29] and there are currently 2.6 billion solar panels in the world generating a total of 865 TWh.[30] From 2019 to 2020, 135 TWh of solar energy was added; the manufacture of these requires 52,000 tons of silver. Worldwide, 27,540 tons of silver were being mined in 2020, and the amount increases by ca. 2% every year.[30] On this trajectory, solar panels would use 100% of global silver supplies in 2031 leaving none for electric car batteries and other uses.

While investments into renewable energy currently have the effect to reduce the price of renewable energy for other sectors, as soon as there are limits to the amount of renewable energy that can be generated, any additional energy used by ICT will take energy away from other purposes. There are also practical constraints on the extent that renewable energy can be used to power ICT equipment. Even data centers that are powered by

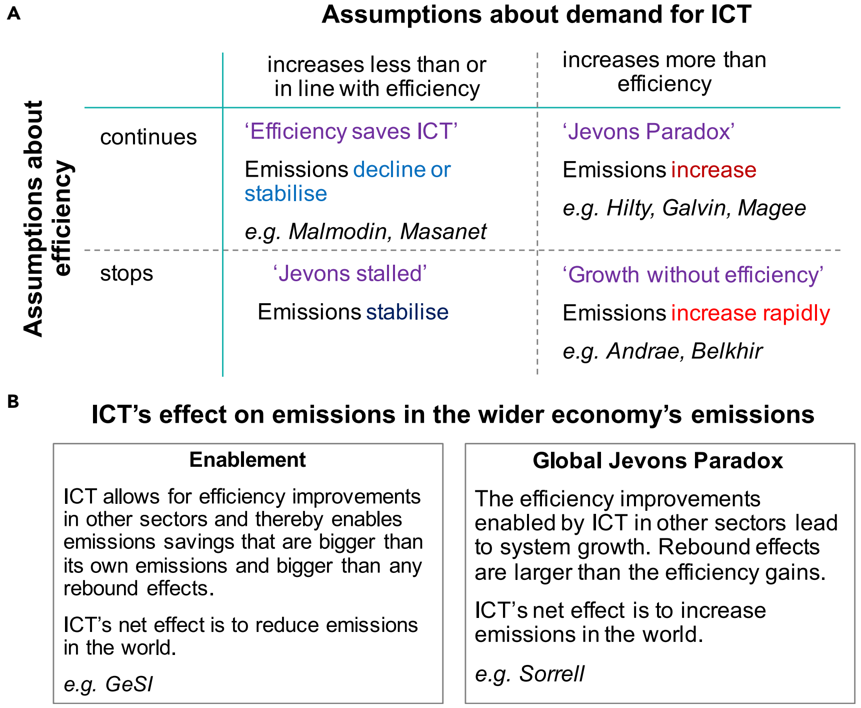

Figure 5. Narratives of ICT's role in climate change and the critical assumptions underlying these

(A) ICT's carbon footprint.

(B) ICT's effects on emissions in the wider economy. The proponents of each narrative are in italics. Efficiency is here defined as GHG emissions per equivalent ICT use. This includes Moore's Law but also higher renewable energy use, energy efficiency of the infrastructure, etc.

100% renewable energy usually have fossil fuel-powered backups for unexpected demand increases. Powering networks with renewable energy is a lot harder due to their decentralized nature,[7] and powering user devices depends largely on the greening of national grids—a trend that is ongoing in the UK but still far from complete. Thus, while a shift to more renewable energy is crucial, it does not provide an unlimited supply of energy for ICT to expand into without consequences.

Six common narratives for ICT's role in climate change

The assumptions from the studies and unpacked in this section can be summarized into six narratives of ICT's future role in climate change (see Figure 5): four around future trends in efficiency and demand and their effect on ICT's own emissions, and two on ICT's effect on emissions in the wider economy.

Summary of ICT's carbon footprint

To meet climate change targets, the ICT sector needs to drastically decrease its own emissions and deliver vast savings in other sectors. Despite some variability in estimates, research studies reviewed here agree that ICT is responsible for several percent of global GHG emissions and that its footprint has grown until recently. The world needs to reduce its GHG emissions to stay within 1.5°C warming.[31] If the ICT sector should decrease its emissions in line with other parts of the economy, it would have to: reduce its CO_2 emissions by 42% by 2030, 72% by 2040, and 91% by 2050 (see Figure 6) and net zero by 2050;[32] or deliver equivalent savings in other sectors in addition to the savings these sectors will have to deliver themselves to meet these targets, making sure that rebound effects do not offset these savings. Global CO_2 emission cuts to 2050 needed to stay within 1.5°C warming by 2100 are based on modeling by Baskerville-Muscutt[33] based on the Shared Socio-Economic Pathway 2 as outlined by the International Institute of Applied Systems Analysis;[34] this is the "middle of the road" or average scenario for the trajectory the world will follow, and cuts are relative to global CO_2 in 2010. Note that this is CO_2 only, assuming ICT emissions are mostly CO_2 as a large part if electricity and there are no agricultural components. The comparison to CO_2 emissions was chosen because reliable budgets do not exist for GHG emissions at this point.

Under business as usual, increases in emissions are likely. Major concerted effort would be needed to reduce emissions. All the analysts we spoke to agree that to decrease ICT's emissions—even assuming emissions have stabilized—a strong and unified effort would be needed (Current policy developments and governance in ICT). Without this effort, even if ICT's emissions were to stay stable at the 2020 level over the next decades, the relative share of ICT's emissions in global emissions would increase to more than a third as other sectors reduce their emissions in line with 1.5°C warming (see Figure 6).

There are three reasons to believe that ICT's emissions are higher than estimated and that they are going to increase. *Reason 1: rebound effects have occurred since the beginning of ICT, and they will likely continue without intervention.* Even if efficiency improvements are continuing (see Are energy efficiency improvements in ICT continuing?), this will not completely counterbalance growth in demand for ICT; in fact, efficiency gains might spur further growth in emissions by allowing the ICT sector to grow further due to rebound effects (see Are energy efficiencies in ICT reducing ICT's carbon footprint?). We believe that a natural peak in ICT emissions due to saturation of demand is unlikely (see Are ICT's emissions likely to stabilize due to saturation?). To the extent that ICT enables efficiency gains in other sectors, there is the risk that rebound effects more than offset any savings following Global Rebounds (see Is ICT enabling carbon savings in other industries?). Renewable energy will help decarbonize ICT but is not a silver bullet (see Will renewable energy decarbonize ICT?).

Reason 2: current studies of ICT's carbon footprint make several important omissions surrounding the growth trends in ICT. The studies reviewed here make several important omissions in areas of ICT growth, such as blockchain and partial consideration of IoT. This leads to an incomplete picture. Some analysts argue that blockchain is not part of ICT because it requires specific hardware, not regular servers. However, we believe that it should be in scope of ICT as it is an ICT-facilitated

CellPress
OPEN ACCESS

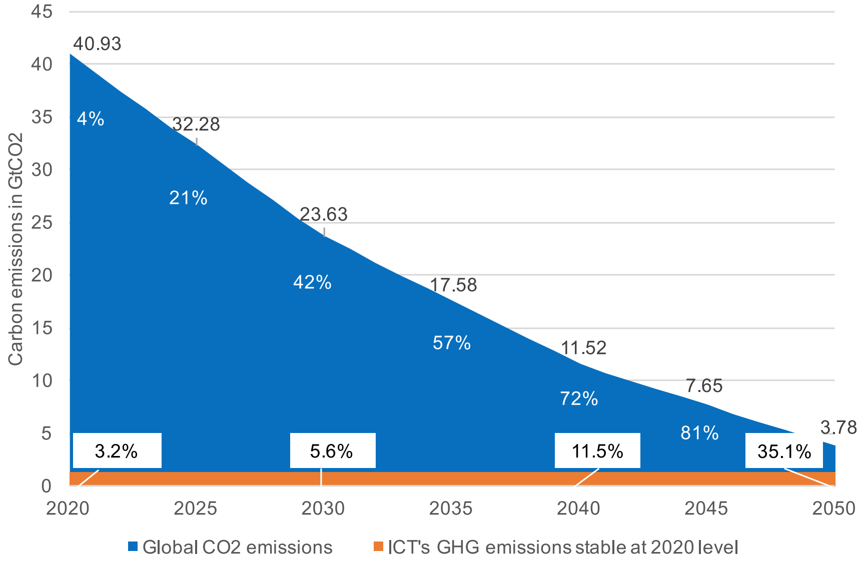

Figure 6. ICT emissions, assuming the 2020 level (adjusted for truncation error) remains stable until 2050, and global CO_2 emissions reduced in line with 1.5°C under scenario SSP2-19

Numbers on the blue slope indicate global CO_2 cuts needed relative to 2010 and labels at the bottom indicate ICT's share of global CO_2 emissions in percent. We assume most of ICT's emissions are from CO_2 because a large proportion of its footprint is from electricity consumption and there are no agricultural components. The comparison to CO_2 emissions was chosen because reliable budgets do not exist for GHG emissions at this point.

algorithm (see Blockchain); having specific hardware for blockchain is similar to how graphics-intensive services (e.g., online games) require graphics processing units (Preist, personal communication). Malmodin and Lundén[8,9] include some IoT and concluded that the impact of IoT is small. However, this is a small share of all IoT and they only accounted for the connected devices, not the energy consumption that IoT creates in data centers and networks (based on the assumption that data traffic and energy are not closely related, see Is data traffic independent of ICT emissions?). Such trends, as well as AI, could help reduce global carbon emissions, but they will also add to ICT's carbon footprint; we discuss this trade-off for prominent ICT trends in the next section (see ICT Trends: Opportunities and threats).

Reason 3: there is significant investment in developing and increasing uptake of blockchain, *IoT and AI.* Despite questionable evidence that ICT growth trends will save more carbon emissions than it will introduce (see ICT Trends: Opportunities and threats), blockchain, IoT, and AI are seeing increased investment and uptake. As we explore in Current policy developments and governance in ICT', the European Commission discuss these trends as a way to spur economic growth and yield emission reductions; yet, they expect ICT will only enable 15% reductions, which is insufficient for meeting climate change targets (see European policy and ICT). Some large technology corporations are setting their own carbon pledges, which might help reduce the emissions from ICT's growth trends; however, these pledges are often not ambitious enough to meet net zero emissions by 2050 (see Self-regulation in the ICT industry). Until ICT corporations become net zero, any investment in the ICT industry will be associated with an increase in emissions.

With a global carbon constraint, ICT will be a vital sector to ensure transition to a net zero world. If a global carbon constraint was introduced, we could be certain that rebound effects would not occur, meaning that productivity improvements through ICT-enabled efficiencies both within the ICT sector and the wider economy would be realized without a carbon cost. Under these conditions, ICT would be a key means by which productivity is maintained or increased despite the carbon constraint, and therefore ICT's role in enabling the whole economy can be expected to be even greater than it is today. Given these reasons, under a carbon constraint, ICT's share of global emissions could justifiably be allowed to rise.

ICT trends: Opportunities and threats

Three recent and emerging innovations may have profound implications for the carbon footprint of the ICT sector: (1) big data, data science, and AI; (2) the IoT; and (3) blockchain and cryptocurrencies. In this section, we explore the opportunities and threats for each, as well as the potential mitigation of such threats.

Big data, data science, and AI

Big data is one of the most significant technology trends, made possible by the vast data and computational capabilities of cloud computing. Arguments have been made for both the opportunities of realizing a "smart" future and potential growth in ICT's carbon footprint.

Opportunities

Big data, data science, and AI could contribute to a lower carbon smart future. Big data/data science/AI and IoT can help bring about a smart and sustainable future encompassing smart grids, cities, logistics, agriculture, homes, etc.[35–38] For example, by finding optimal routes through cities and reducing traffic congestion, or by optimizing energy use for building heating and lighting. As these areas rely on IoT, we defer discussion on these opportunities until Internet of Things.

There is a willingness across industry and academia to apply such technologies for the benefits of society. There is a significant move toward data science and/or AI for social good, including applications in health[39] and the environment, although this work is in its infancy and generally not in everyday practice. The role of big data in supporting green applications has been discussed in the areas of energy efficiency, sustainability, and the environment;[40] and the field of computational sustainability is emerging, using technologies, such as AI, in support of the United Nations (UN) sustainable development goals.[41] There is also an emerging research community looking at the role of such technologies in supporting environmental sciences as

they seek a deeper understanding of our changing natural environment. See, for example, research in Toronto, Exeter and the Center of Excellence in Environmental Data Science, a joint initiative between Lancaster University and UK Center For Ecology & Hydrology program called ''data science for social good.''

Threats

The world's data are doubling every 2 years. Data has been described as ''the new oil''[42] given its commercial impact—yet as data storage and data centers grow to meet demand, this description could have a double meaning due to its environmental impacts. Data can help solve complex world problems, but there are concerns over the resources required to facilitate data science and AI, especially the carbon footprint of data centers (see Estimating the carbon footprint of ICT). The total size of the world's digital data was estimated to be 59 zettabytes in 2020, with the amount of data created in the following 3 years expected to be more than the data created in the last 30 years.[43] AI and data science are therefore an important trend that drives growth in data storage and processing (data processing will be the larger contributor to ICT's energy use, as simply storing data is environmentally cheap in comparison [Preist, personal communication]) and in data centers, which some experts argue leads to an increase in ICT's carbon footprint (Is data traffic independent of ICT emissions?).

Emissions associated with processing this data are increasing due to growing computational complexity. Data science and AI offer additional threats over and above the potential growth of data center emissions. AI has the greatest potential for impact given the complexity of training and inferencing on big data, and especially so-called deep learning. Researchers have estimated that 284,019 kg of CO_2e are emitted from training just one machine learning algorithm for natural language processing, an impact that is five times the lifetime emissions of a car.[44] While this figure has been criticized as an extreme example (a more typical case of model training may only produce around 4.5 kg of CO_2),[45] the carbon footprint of model training is still recognized as a potential issue in the future given the trends in computation growth for AI:[45] AI training computations have in fact increased by 300,000× between 2012 and 2018 (an exponential increase doubling every 3.4 months).[46] Further adding to the threat of AI, ICT companies have been found to use such computationally intensive algorithms for advancing the fossil fuel industry.[47]

Threat mitigation

Sustainability needs more consideration in ethical guidelines of AI. Due to this growth of computation, Schwartz et al.[48] argue the need for ''Green AI'' that focuses on increasing the efficiency of AI computation rather than the current focus on what they describe as ''Red AI,'' i.e., accurate AI models trained without consideration of resource costs. Sustainability is currently one of the least represented issues associated with ethics guidelines in AI,[49] although a framework and ''leaderboard'' to track the energy consumption and carbon emissions of machine learning has recently been offered in the hope that this will encourage energy efficiency to be considered.[50] Improvements in efficiency and opportunities may exist, such as addressing the processing requirements of AI algorithms by using idle PCs as a distributed supercomputer.[51] However, we reiterate the earlier concerns that an efficiency-focused endeavor without a carbon or consumption constraint may fail to mitigate rebound effects (see Are energy efficiencies in ICT reducing ICT's carbon footprint?).

The IoT

The IoT represent a set of everyday internet-connected objects from wearable technologies through to appliances, cars, and other transport vehicles. This has led to a substantial and ongoing growth of the internet as documented below.

Opportunities

IoT technologies can enable efficiency improvements outside of the ICT sector. IoT applications are often viewed as ''smart technology,'' especially when combined with data science/AI in ways that optimize energy usage more widely. Smart cities aim to provide better public services at a lower environmental cost,[52] e.g., location-based services from smart city IoT sensing and data analysis can reduce transportation pollution through more efficient driving routes.[53] Govindan et al.[54] also investigate how such developments can support smarter logistics, including reducing energy requirements. As mentioned in Will renewable energy decarbonize ICT?, ICT has the potential to decarbonize the energy supply and a combination of IoT and the power grid has real potential to enable the Smart Grid, e.g., by dealing with intermittency of renewable supply.[55] IoT deployments have been tested in schools with the aim of raising awareness of energy consumption and ''promoting sustainable behaviors,''[56] and IoT has also been harnessed to enable energy efficiency improvements within ICT, e.g., by using IoT to reduce air conditioning for data centers.[57] These few examples highlight the breadth of IoT opportunities to reduce GHG emissions, as long as the IoT applications *substitute* more carbon-intensive activities rather than act alongside them.

Threats

IoT enablement comes at a cost of rapidly rising numbers of devices, device traffic, and associated emissions. The sheer number of IoT devices and the associated data traffic is growing significantly. Innovation in IoT is expected to create a 5-fold increase from 15.41 billion internet-connected devices in 2015 to 75.44 billion in 2025.[58] Cisco estimate machine-to-machine (M2M) connections will grow from 6.1 billion in 2018 to 14.7 billion by 2023 (a compound annual growth rate [CAGR] of 19%), representing 1.8 M2M connections per member of the global population in 2023.[22] The majority of these connections is expected to be formed by IoT in the home for automation, security, and surveillance (48% of connections by 2023), yet connected cars (30% CAGR between 2018 and 2023) and cities (26% CAGR) are the fastest growing IoT sectors.[22]

IoT's carbon footprint is under-explored, but will have significant implications for embodied emissions. While the footprint of IoT is uncertain and often unexplored in studies of ICT carbon emissions (Are ICT's emissions likely to stabilize due to saturation?), it has been estimated that the energy footprint of IoT semiconductor manufacturing alone might be 556 TWh in 2016 and increase 18-fold to 722 TWh in 2025.[59] This does not include other aspects of embodied carbon in IoT, such as material extraction and transport, or sources of GHG emissions other than electricity; it also does not consider energy use of running systems, although Das[59] estimates that this would be a lot smaller than the embodied carbon in manufacturing, at perhaps 118 TWh in 2016 and decreasing to only 1 TWh in 2025 as we see

more energy efficient technologies. This study has also, however, been questioned as being vastly overestimated by Malmodin (personal communication). Assuming a global electricity mix of 0.63 $MtCO_2e$/TWh, this would be a total of 424 $MtCO_2e$ in 2016 and 6,125 $MtCO_2e$ in 2025 for the manufacture and use of the semiconductors; this is without emissions from the entire IoT device, associated sensors, and the emissions in data centers and networks that IoT communicate with. It is also worth noting that the introduction of IoT could lead to an initial rise in obsolescence for other non-ICT products, as society makes the transition to an IoT-focused life (e.g., replacing a working kettle with an internet-connected kettle).

Threat mitigation

Lower energy IoT systems are a way forward, but may lead to energy-intensification and fuel greater emissions overall. Researchers are already looking to create lower energy IoT systems, considering both devices[60] and communication technologies. One focus is on Low Power Wide Area Networks (LPWANs)[61] to reduce the energy requirements of M2M communication, but at a trade-off of lower bandwidth. There is an associated field of study referred to as "Green IoT,"[62–65] which focuses on ensuring that IoT's own environmental costs are considered as we move toward a smarter society and environment. Yet we should be careful of IoT applications that could lead to rebound effects. For example, smart home technologies have the potential to reduce energy consumption (e.g., through remote-controlled heating or lighting), but could perhaps lead to "energy-intensification" once adopted through offering new services (e.g., pre-heating homes, continuously running security systems) or intensifying current services (e.g., internet connectivity, audio/visual entertainment)[66]—the latter adding to ICT's carbon footprint through additional user devices and data traffic.

Blockchain

Blockchain is an example of a decentralized algorithm designed to avoid a centralized authority or central point of failure. Blockchain allows for potentially important new uses, e.g., for decentralized financial systems. Cryptocurrencies are the most popular application for blockchain, with Bitcoin being the biggest cryptocurrency available today.

Opportunities

Blockchain could offer some opportunities for reducing carbon, but there are no emissions-reducing applications of these technologies yet. A decentralized electronic currency could offer a real disruption in the management of market transactions and in the possibility of handling decentralized energy exchanges,[67] although there are no real examples of demonstrable emissions savings yet. Kouhizadeh and Sarkis[68] discuss the potential of blockchain technologies to enhance sustainability in the supply chain, for example, by supporting transparency in the early stages of supply chain management (e.g., vendor selection and evaluation); this work, however, is speculative at this stage, leading to researchers offering directions to further explore adoption of blockchain in this domain.[69]

Threats

The energy consumed by single cryptocurrency is equivalent to that of entire nations. Blockchain is underwritten by energy: the algorithm, if based on "proof of work," creates high levels of replication and redundant computation.[70] The methodology and assumptions behind Mora et al.'s[70] projections of blockchain's future energy use have been questioned by Masanet et al.,[11] but proof of work is widely accepted to be energy-intensive. Energy consumption can also increase through escalation of the "mining arms race" due to improving risk sharing for proof of work blockchains.[71] Focusing on cryptocurrencies, one study indicates that Bitcoin's annual electricity requirements of 68.7 TWh in 2020 are equivalent to powering 7 million US households,[72] associated with a footprint of 44 $MtCO_2$. This is based on a global average electricity intensity of 0.63 $kgCO_2e$/kWh, which is likely an underestimate since the energy used to mine Bitcoin often draws on a higher share of coal than the global average.[73] Due to the inefficiency of transactions, a single transaction could be ca. 750 kWh, enough to power 23 households for 1 day,[72] or 473 $kgCO_2e$—also based on the (likely underestimated) 0.63 $kgCO_2e$/kWh global average electricity intensity. Bitcoin currently has a market dominance of 64% of all cryptocurrencies.[74] Under the assumption that other cryptocurrencies have the same carbon intensity as Bitcoin, the carbon footprint of all cryptocurrencies would be ca. 69 $MtCO_2e$, 0.1% of global emissions. Another study estimated the Bitcoin network electricity consumption at 2.55 gigawatts (GW) in 2018 (a value that is nearly as much as Ireland at 3.1 GW), but that this could rise to 7.67 GW in the future (making it comparable with Austria at 8.2 GW).[75] Other researchers argue an annual electricity consumption of 48.2 TWh and annual carbon emissions ranging from 23.6 to 28.8 $MtCO_2$ for Bitcoin in 2018.[73] Stoll et al.[73] also estimated that other cryptocurrencies would add another 70 TWh in 2018, bringing the total carbon footprint to ca. 73 $MtCO_2e$ in 2018.

Threat mitigation

Fiscal policy intervention may be needed to mitigate energy consumption of decentralized algorithms. Alternatives to proof of work exist that could reduce the resources required for blockchain, e.g., proof of stake reduces computation and Byzantine protocols remove consensus mining.[76,77] Carbon offset mechanisms for blockchain also exist, such as SolarCoin, whereby solar energy producers are rewarded with a free SolarCoin for each MWh of solar-based electricity they produce.[78] Renewable energy can also be used to power these technologies and it is argued to form 73% of Bitcoin's mining,[79] although it is important to note that CoinShares Research who published the report run a cryptocurrency investment fund, so there is a potential conflict of interest. However, de Vries[80] does not think Bitcoin can be sustainable due to: (1) the seasonality of hydropower in Sichuan, China (a region that supposedly supports nearly half of global mining capacity)[81] meaning energy is required from alternative sources such as coal; and (2) the e-waste associated with mining machines once they reach their end-of-life (if the cryptocurrency collapses, mining machines cannot be repurposed as a generic data centers since they are so specialized [Preist, personal communication]), estimated at an annual 10,948 metric tons (comparable to Luxembourg at 12 kt) assuming Koomey's efficiencies law.[82] Despite being the most popular use of blockchain technology, there are, and will continue to be, blockchain applications beyond Bitcoin and cryptocurrencies. To mitigate the energy consumption of blockchain technologies and applications, Truby[83] has proposed a series of fiscal policy options, such as

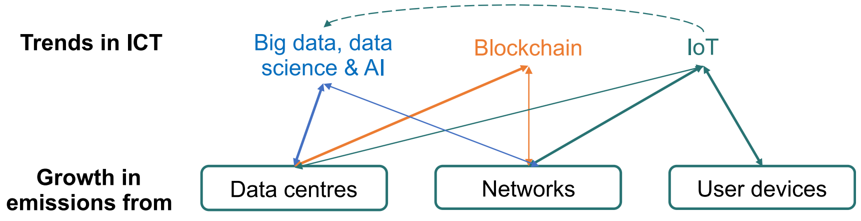

Figure 7. The impacts that trends in ICT have on growth in emissions from data centers, networks, and devices
Note that the thicker lines depict prominent threats, thinner lines depict secondary threats, and the dotted lines depict the links between the trends.

introducing a customs duty or excise tax on imports of miners' verification devices based on its energy consumption.

Summary of ICT trends

If unchecked, ICT trends could drive exponential growth in GHG emissions. The three trends we have discussed could lead to substantial growth in ICT's footprint (see Figure 7 and note that in this section we expand ''user devices'' to ''devices'' to include embedded devices). While we have discussed the trends independently, it is important to note that these trends are in fact interlinked. For example, IoT involves collecting more data from sensors, requiring more analytics and adding to the issues raised by big data, data science, and AI, with the potential to further increase ICT's emissions. Such growth trends will also be facilitated through innovations in the ICT infrastructure, e.g., the move from 4G to 5G cellular networks would enable faster, data-intensive network transmissions for IoT devices—allowing for even more data to be collected, communicated, and processed. If not restrained, these above trends all have potential to help drive further exponential growth, unlikely to be outweighed by the ICT-enabled carbon reductions in other sectors.

COVID-19 has shown a consumption constraint that could disrupt these trends. As many activities have been restricted or avoided during the pandemic, ICT has shown the significant benefits and value it can bring to society—allowing families to communicate, people to work from home, and conferences to be held online. Under these circumstances, ICT serves as *a substitution* rather than *an addition* to our regular activities. Coinciding with this, there has been a temporary drop in carbon emissions. A recent study in Nature estimates that daily global CO_2 emissions temporarily decreased by 17% in early April relative to 2019 levels, largely due to changed transport and consumption levels, and that 2020 annual emissions could decrease by 4% if restrictions remain in place until the end of 2020, and 7% if restrictions end in June relative to 2019.[84] However, this is negligible if it does not lead to lasting changes after the pandemic. The key question is what society will do when the COVID-19 crisis is over. Will the world embrace some of the new ways of living and working instead of their traditional counterparts and reap the carbon benefits, or return to the old ways, or a mix of the two?

There are important policy decisions to be made that determine the future of ICT's carbon footprint. There is an increasing awareness of the impacts of ICT, but we note the need to expand our awareness to the full range of narratives and their underlying assumptions (see ICT's future carbon footprint: Unpacking the studies' assumptions). We also note that ICT and its trends can bring a lot of value to many people worldwide. Society is very much at a crossroads in terms of the choices faced, and there are some positive signals. For example, in AI research, there have been calls for the EU to incentivize AI applications that are ''socially preferable (not merely acceptable) and environmentally friendly (not merely sustainable but favorable to the environment),'' recognizing the need for a methodology to assess these characteristics.[85]

Without a global carbon constraint, avoiding unsustainable growth in ICT becomes a debate of what we should prioritize in the ICT sector, what problems can and should be solved using computing, and who can access the required ICT resources for such solutions—supporting valued use of ICT (for example, for uses that lead to carbon reductions in the economy) while constraining consumption and minimizing the ICT sector's carbon footprint. An example of such prioritization in practice is the recent Netflix agreement with EU regulators to reduce its bitrate to ease the burden on the internet during the COVID-19 outbreak, enabling more people to work online from home.[25] This in turn places the spotlight on policy makers and governance structures at all levels, including in industry, governments, and academia. We look at this important issue in the next section.

CURRENT POLICY DEVELOPMENTS AND GOVERNANCE IN ICT

Self-governance and the policy landscape is changing. Europe is leading the world in implementation of and experimentation with climate policy,[86] making the EU Green Deal and the European Commission's (EC) rhetoric particularly worthy of analysis as a bellweather of global climate policy. In this section, we explore such European policy, and also look at self-regulation of ICT emissions by top technology companies to understand whether they are sufficiently ambitious to meet carbon targets without the need of top-down regulation.

European policy and ICT

ICT is a central pillar of Europe's climate strategy. Under the EC's Green Deal, Europe is committed to becoming carbon neutral by 2050, and climate neutral later this century.[87] The EC use the term ''carbon neutral'' to refer to no net emissions of carbon dioxide, and the term ''climate neutral'' to refer to no net emissions of GHG emissions. This is different from the way most ICT companies use the term ''carbon neutral,'' which includes all GHG emissions. ICT features prominently in policymaking around the climate: (1) because of recent efforts to lead the world in a sustainable, human-centric approach to innovation,[88] and (2) to drive down GHGs across the economy.

European ICT emissions policy emphasizes efficiency, renewables, and circular waste. The EC's official figures put ICT's current share of global GHG emissions at more than 2%,[89] and a study commissioned by the EC anticipates that ''the energy consumption of data centers and telecommunication networks will grow with an alarming rate of 35% and 150% respectively over

CellPress
OPEN ACCESS

9 years" (from 2018).[90] Rather than seeking to directly affect this consumption trend, policy focuses on mitigating the impacts of rising consumption, specifically through improved efficiency and renewable energy. Three fundamental assumptions are evident in this approach: (1) there is scope for energy efficiency improvements in ICT to continue, at least through 2050 (Are energy efficiency improvements in ICT continuing?); (2) energy efficiency gains in ICT can reduce ICT's carbon footprint (Are energy efficiencies in ICT reducing ICT's carbon footprint?); and (3) renewable energy will decarbonize ICT (Will renewable energy decarbonize ICT?). As we have discussed in Summary of ICT's carbon footprint, there are strong arguments against each of these premises that may impede successful decarbonization of ICT unless simultaneously curbing demand or adding a global carbon constraint. However, publicly facing policy statements do not attend to these counter-assumptions.

Data centers are a particular focus of European policy. The EC has committed to carbon neutral data centers by 2030, through a mixture of continued efficiency improvements, transitioning toward reliance on renewable energy sources, and developing methods of reusing the heat that servers generate.[91] This is an ambitious proposal, as currently there is no indication that data center emissions are decreasing despite continuous efficiency improvements (see ICT's carbon footprint). The EC also does not specify whether this must be achieved through on-site renewables or can include purchasing of offsets.

Other noteworthy policy covers e-waste, which is recognized by the World Economic Forum as the fastest growing category of waste.[92] As part of Europe's New Circular Economy Action Plan, the EC plans to put forward a "Circular Electronics Initiative" by the end of 2021 to improve the lifespan, repairability, and recyclability of ICT products.[93] This initiative would help decrease the embodied carbon of ICT but would be partly offset if the total number of devices continues to increase (i.e., innovation will prohibit saturation in ICT, see Are ICT's emissions likely to stabilize due to saturation?).

Except for this Circular Electronics Initiative, which will likely include a reward scheme for consumers who recycle their old devices,[94] the Green Deal is notable for its lack of clear incentivization or enforcement mechanisms regarding decarbonization of ICT. It may be believed that efficiency naturally improves as technology advances (e.g., through Moore's Law), and/or that market forces will compel industry to drive these improvements, as there is no discussion of either penalties to be applied or assistance to be offered to the sector toward achieving carbon neutrality by 2050. Also not provided within the Green Deal are estimates of the emissions reductions needed within the ICT sector itself to meet this ambition, which may be incompatible with continuing growth expected of ICT's electricity consumption (see ICT's carbon footprint).

Europe seeks to supercharge enablement through significant investment in ICT. While policies clearly acknowledge ICT's share of global emissions and commit to reducing them, the primary thrust of Europe's climate strategy is the use of ICT to enable emissions savings in other industries ("enablement"). An EC commissioned report states vaguely that ICT "probably saves more energy than it consumes."[90] The wording of the Green Deal, however, is unambiguous: "Digital technologies are a critical enabler for attaining the sustainability goals of the Green deal in many different sectors."[95] This includes various initiatives and major funding schemes intended to foster innovation in and uptake of AI, IoT, and blockchain.

The Green Deal does not provide a detailed roadmap for how these technologies will in fact deliver against these goals, nor figures regarding expected savings to be achieved. These are undoubtedly difficult to estimate, but as yet there is no evidence in the multi-decade history of ICT-driven efficiency savings that enablement works for reducing overall emissions (see Is ICT enabling carbon savings in other industries?). In the absence of an intervention, such as the introduction of a global carbon constraint, claims of the feasibility of this strategy should be approached with skepticism. As a baseline, staying below 1.5°C warming would require the global economy to reduce by 42% by the year 2030, including the ICT sector (see Summary of ICT's carbon footprint); so if ICT's emissions do not shrink by 42% by 2030, then it would have to enable reductions in other sectors—beyond the 42% that other industries will have to cut anyway—to compensate for this shortfall. This may prove a delicate balancing act. To facilitate this work, complete and accurate estimates of ICT's footprint need to be captured regularly, alongside careful accounting of the emissions ICT is driving or saving in other sectors, with sector targets adjusted accordingly to ensure regional and global targets are met. For this, consistent carbon accounting standards would need to be established across the sector; this would avoid the variability of carbon estimations, as we found with current studies in Estimating the carbon footprint of ICT, from differences in the approaches, boundaries, and data used.

We note the competing policy priorities of the EC. Europe faces pressures to remain competitive in the global technology market and seeks to lead the way in rapidly growing technologies that would otherwise be capitalized by Asian and US competitors.[96] By stimulating innovation in these areas, Europe seeks to maintain both the health of its economy and the health of the planet. But critically, in the current policy environment, and lacking a global carbon constraint, economic growth would likely further spur consumption and therefore emissions.

Self-regulation in the ICT industry

Companies need net zero carbon targets that cover supply chain emissions. Several big ICT companies have recently announced carbon pledges to self-regulate their emissions (e.g., Amazon, Apple, BT, Microsoft, Sky). These pledges fall into three main categories: (1) carbon neutral (least ambitious); (2) net zero; and (3) carbon negative (most ambitious). To limit global warming to 1.5°C,[31] we will need to reach net zero emissions by 2050 globally.[97] Companies should aim for net zero or, even better, carbon negative. To make this possible carbon neutral targets are not enough because they do not cover supply chain emissions. Yet only a few firmly aim to be net zero (e.g., Microsoft, Sky, Amazon, BT), and only Microsoft aims to be carbon negative.[98]

Carbon offsetting requires truly additional carbon removal methods. Companies need to prioritize reducing the total emissions as much as possible[99]—only then should the rest of their emissions be offset by permanent, verifiable, and additional carbon removal methods. For a company's emissions to be truly offset, the same amount of carbon that the company emits

needs *to be removed* from the atmosphere (e.g., through afforestation, reforestation, planting seagrass, taking in landfill gas), not simply avoided. An example of an avoided emission offset is an area of forest that is protected from logging; the amount of carbon that would have been released if the forest was cut down is counted as offset. However, there needs to be some certainty that it would have been removed if it had not been purchased, otherwise these offsets cannot be considered additional. Even genuine "avoided" emissions may end up "leaking" out at another point in the system (e.g., a protected area of forest may just lead to more logging somewhere else in the world).[100]

Only 2% of offsets result in truly additional removals.[101] Furthermore, some offsetting projects may not be permanent: where forests or peatlands are used to sequester carbon, these carbon stores must be protected from fires or logging—otherwise the carbon removals are negated. Efficiency enablement cannot count as offsetting because it is hard to show that any enabled savings are not negated by rebound effects (see Is ICT enabling carbon savings in other industries?).

Only some renewable energy helps to cut emissions. Some companies also claim, or aim for, power provision from 100% renewable energy without specifying whether they aim to cut emissions. Companies need to detail which type of renewable energy they use (e.g., biofuels, solar, wind, hydro), and what proportion of their renewable energy comes from on-site renewable power generation, Power Purchasing Agreements (PPAs), and Renewable Energy Guarantees of Origin (REGOs), as these differ in their additionality. For a company to claim they are 100% renewable, they should source 100% of their energy through PPAs, on-site renewables, and investment in off-site projects but not unbundled REGOs, because the latter cannot claim additionality. Renewable energy projects should not be considered a removal but rather a scope 2 reduction (see Will renewable energy decarbonize ICT?).

The new ITU standard encourages ICT companies to become net zero by 2050. In collaboration with GSMA, GeSI, and SBTi, the International Telecommunication Union (ITU),[32] a UN agency focused on the ICT industry, released a new standard in February 2020. The standard aims to reduce ICT's GHG emissions by 45% by 2030, and net zero by 2050, in line with limiting global warming to 1.5°C. The scope of ITU's recommendation includes "mobile networks, fixed networks, data centers, enterprise networks, and end-user devices, but excludes ICT services." The "voluntary" standard comes with reduction targets for each ICT sub-sector for the next decade. Sub-sectors are defined as per other ITU documentation, specifically clauses A2 to A6 of ITU-TL.1450.[102] Data center operators adopting the science-based target will need to reduce emissions by at least 53%, mobile network operators by 45% and fixed network operators by 62%.[12] The targets have been approved by the SBTi and require companies to set targets for scope 1 and 2 emissions and some supply chain scope 3. Most of these reductions between 2020 and 2030 are expected to come from a shift to more renewable and other low-carbon energy sources. The targets are less ambitious than pledges by individual companies, such as BT, Sky, and Microsoft, which commit to reach net zero by 2030 or 2040, but they send a strong signal that the world needs net zero and science-based targets and provide a template that policy makers could adopt.

Key implications for policy moving forward

The full climate impacts of ICT need to be considered systematically, accounting for end-to-end life cycles and supply chain emissions. It is critical that complete and accurate estimates are used to guide climate policy making and target setting within the sector. Studies of ICT's carbon footprint should strive for interrogatability, but also need to disclose potential conflicts of interest that may affect boundary setting for such calculations. Where technologies are unlikely to be included within the estimates of other sectors' carbon footprints, it is essential that they are included in estimates of ICT's footprint so that climate impacts can be accurately monitored across the economy. It is also vital that calculations do not conflate efficiency improvements with emissions reductions, and that they use methods that allow for objective, high-quality, and up-to-date data and analysis—rectifying the issues of current estimates (see ICT's carbon footprint). This also supports the recommendations by Dobbe and Whittaker[47] who lobby for carbon transparency, as well as consideration of the full supply chain and rebound effects in carbon accounting.

While ICT offers opportunities to enable reductions in CO_2 emissions in other sectors, evidence does not support their ability to achieve the significant carbon savings required by 2050. It is important not to overhype ICT's potential to reduce emissions across the economy, thus additional research is sorely needed to provide robust estimates to policy makers. Continued growth in the carbon footprint of the ICT sector cannot be justified on the basis that these technologies may enable sufficient savings in other sectors—particularly as estimations of ICT-enabled emissions savings in other sectors fall short of what is required for meeting agreed targets, and there is a risk that ICT's expansion into other sectors could increase those sectors' emissions (see European policy and ICT). This fundamentally calls into question the presumed role of efficiency within climate strategy. There is clear need to detail sector by sector the savings ICT is expected to produce—reflecting careful balancing of sector footprints within the contexts of regional and global targets—along with developing a detailed roadmap toward delivering on those expectations.

The ICT sector must adopt science-based net zero targets in line with, or better than, the ITU standard; but industry self-regulation may not be sufficient to yield necessary emissions reductions. With growing awareness of the climate emergency, public pressure may be enough to get more ICT companies to announce net zero emissions by 2050. However, there is a lack of net zero pledges thus far. Some companies that have pledged net zero are not on target, or do not have detailed and transparent action plans. Note that this piecemeal approach of individual companies making commitments also comes at a competitive cost for the forериders, with others gaining financially from being free from such commitments. The way forward for a reduction in ICT's emissions is a sector-wide commitment to net zero that is enforced through incentives and compliance mechanisms, such as procurement clauses that set out carbon criteria and consequences for non-compliance. We flag this as an important issue for the sector but detailed consideration of the form of regulation is beyond the scope of this paper. We also note that an ICT-focused net zero commitment is unlikely to limit

CellPress
OPEN ACCESS

the emissions from ICT's impact on the wider economy, unless upstream scope 3 emissions are included in the targets.

There is a pressing need to devise a strategy for constraining consumption of ICT so that efficiency improvements lead to actual emissions reductions and enable productivity to be maintained in a carbon-constrained world. It is likely that unabated growth in demand for ICT will more than offset the emissions saved through improved efficiency of these technologies. The only condition under which these rebound effects would not apply is if a constraint were applied, such as a constraint on consumption or an economic constraint through rising carbon costs (e.g., a carbon tax or a cap on emissions). Policy-enforced carbon caps on global emissions, or carbon pricing for all industries, would help avoid the risk of Global Rebounds; but without a global carbon constraint, policies will be needed to enforce credible and ambitious carbon pledges within the ICT sector (see Self-regulation in the ICT industry). We have outlined below five criteria specifically for ICT sector targets, all of which will need to pervade the ICT sector and be subjected to tough, well-resourced, and independent scrutiny:

1. targets should be inclusive of scope 1, 2, and 3 emissions
2. reduction trajectories should be in line with IPCC recommendations for limiting warming to 1.5°C
3. where transition to renewable energy is part of the decarbonization pathway, a careful test should be applied that the renewables are provably additional
4. emissions offsets need to pass tests of *permanence*, *verifiability*, and *additionality*
5. where "net zero" or "carbon neutral" targets are announced, these should be disaggregated into an emissions reduction component and an offsetting component so that offsets are not allowed to replace reduction responsibilities
6. emission reduction targets should not be replaced by enablement claims due to the risk of rebound effects

Top-down, deliberate direction of ICT research and development may be needed to meet global carbon targets. In a world where consumption of ICT needs to be constrained, "worthy" uses of ICT may need to be weighed against other "less worthy" ones. The ICT sector plays an essential role in helping people live better, and it needs to continue to do so while carefully managing demand. Binding commitments to emissions targets for the ICT sector are needed to force decision making that prioritizes the environment over profit when these are in conflict. Unprecedented coordination across the sector in collaboration with policy makers is required to design and enact a plan for achieving net zero emissions from ICT by 2050.

DISCUSSION AND CONCLUSIONS

As we have explored in this report, there are two central issues for the ICT industry with respect to the climate emergency: ICT's own carbon footprint; and ICT's carbon impact on the rest of the global economy. There has been surprisingly little research into these questions given their significance in response to climate change. The evidence that does exist needs to be interpreted with awareness of problems arising from the following issues: (1) the age of the data; (2) a lack of data interrogatability; (3) a potential for conflict of interest (especially where researchers are employed by ICT companies, and data and analysis is not freely available); and (4) varying approaches to, and lack of agreement on, the boundaries of the analysis of specifically what constitutes the ICT industry in terms of inclusion in estimates of its carbon footprint (e.g., whether or not growth trends in ICT such as blockchain are included, how scope 3 emissions in the supply chain are included to avoid truncation error).

Historically we can be sure that four phenomena have gone hand in hand: ICT has become dramatically more efficient; ICT's footprint has risen to account for a significant proportion of global emissions; ICT has delivered increasingly wide-ranging efficiency and productivity improvements to the global economy; and global emissions have risen inexorably despite this.

Looking to the future, our concerns are that this growth in emissions will continue at a time when emissions *must shrink*. All analyses reviewed in this report concur that ICT is not on a path to reduce emissions in line with recommendations from climate science *unless additional steps* are taken by the sector, or legislators, to ensure that this happens. Prevalent policy emphasis on efficiency improvements, use of renewables and circular electronics is likely insufficient to reverse ICTs growth in emissions. There are real concerns that the period governed by Moore's Law is coming to an end, and there is huge investment in trends that can significantly increase the carbon footprint of ICT, including in AI, IoT, and blockchain. Recently there are encouraging signs that some ICT giants may be moving in a positive direction (e.g., through net zero and carbon-negative targets that include their supply chains), yet there is a lack of policy mechanisms for enforcing sector-wide climate target compliance. Our hope is that with the right policy to enforce these commitments, ICT companies will be able to deliver on their pledges and that other industries will follow ICT's example, allowing us to stay within 1.5°C warming.

Based on the evidence available, it is also key that regulators move away from the presumption that ICT *saves more emissions than it produces*—at the very least it would seem unsafe to assume that ICT efficiencies bring about carbon savings by default. While ICT offers opportunities to enable reductions in GHG emissions in other sectors, evidence does not support their ability to achieve the sustained significant carbon savings we require by 2050. And while ICT might make lower carbon living possible, this will not in itself help to bring about a cut in carbon, and conceivably may lead to rebound effects leading to higher emissions overall. The argument of enablement simply does not exempt the ICT sector from addressing its own emissions, and the sector could certainly do more to understand its enablement and rebound effects. To ensure current technologies have a truly positive impact on the environment, the climate emergency requires a global constraint such as a carbon cap on extraction, a price on carbon emissions, or a constraint on consumption, to rule out rebounds in emissions. With this in place, the ICT-enabled carbon reductions could be realized, and the ICT industry could become a vital sector for the transition to a net zero world.

EXPERIMENTAL PROCEDURES

Resource availability

Lead contact

Any queries related to our review resources should be directed to Kelly Widdicks (k.v.widdicks@lancaster.ac.uk).

Materials availability

No new unique reagents were generated as a result of our review.

Data and code availability

The data from our figures is available on Lancaster University's Pure research repository here: https://doi.org/10.17635/lancaster/researchdata/477. Belkhir requested their raw data were kept confidential for Figure 4, so this is not available for the relevant.csv file in the repository. No code was used for the analysis of the data in this review, but we did draw on research by Small World Consulting (SWC) Ltd. into sector emissions to adjust estimates by the key studies in Estimating the carbon footprint of ICT for truncation error; details about this research are provided in the supplemental information (Appendix A.5.4).

SUPPLEMENTAL INFORMATION

Supplemental information can be found online at https://doi.org/10.1016/j.patter.2021.100340.

ACKNOWLEDGMENTS

This work was developed following discussions at the Royal Society project on Digital Technology and the Planet. The research was partially supported by the DT/LWEC Senior Fellowship (to G.S.B.) in the Role of Digital Technology in Understanding, Mitigating and Adapting to Environmental Change, EPSRC: EP/P002285/1, and by the EPSRC Doctoral Prize (to K.W.) to enable K.W. to continue her research in sustainability and digital wellbeing, EPSRC: EP/R513076/1. The research was also partially sponsored by an independent scientific academic body committed to the advancement of high-quality science, who would prefer not to be named. We thank the experts we consulted for this research as well as those who provided feedback, particularly: Anders Andrae, Lotfi Belkhir, Livia Cabernard, Peter Garraghan, Jens Malmodin, and Chris Preist. We also thank the *Patterns* reviewers for their insightful comments and feedback.

AUTHOR CONTRIBUTIONS

Conceptualization, all authors; methodology, C.F. and M.B.-L.; investigation, C.F., M.B.-L., K.W., B.K., and G.S.B.; data curation, C.F., M.B.-L., and K.W.; writing – original draft, all authors; visualization, C.F.; supervision, B.K., G.S.B., M.B.-L., and A.F.; project administration, C.F. and M.B.-L.; funding acquisition, C.F., M.B.-L., G.S.B., K.W., and B.K.

DECLARATION OF INTERESTS

Charlotte Freitag is an employee at Evenlode Investment Ltd. Mike Berners-Lee is the founder and principle consultant of Small World Consulting. Bran Knowles is a member of the ACM Europe Council, and the ACM Europe Technology Policy Committee, where she leads the standing group on climate change. Gordon Blair is a Research Fellow in the UK Center for Ecology and Hydrology (UKCEH) and is a member of the *Patterns* advisory board.

REFERENCES

1. Ritchie, H., and Roser, M. (2019). How Have Global CO_2 Emissions Changed over Time? (Our World in Data). https://ourworldindata.org/co2-and-other-greenhouse-gas-emissions#how-have-global-co2-emissions-changed-over-time.
2. Jarvis, A.J., Leedal, D.T., and Hewitt, C.N. (2012). Climate–society feedbacks and the avoidance of dangerous climate change. Nat. Clim. Change *2*, 668–671.
3. Anders, S.G.A., and Edler, T. (2015). On global electricity usage of communication technology: trends to 2030. Challenges *6*, 117–157.
4. Anders, S.G.A. (2019). Prediction studies of electricity use of global computing in 2030. Int. J. Sci. Eng. Invest. *8*, 27–33.
5. Anders, S.G.A. (2019). Comparison of several simplistic high-level approaches for estimating the global energy and electricity use of ICT networks and data centers. Int. J. *5*, 51.
6. Anders, S.G.A. (2019). Projecting the Chiaroscuro of the Electricity Use of Communication and Computing from 2018 to 2030 (Researchgate.net).
7. Belkhir, L., and Ahmed, E. (2018). Assessing ICT global emissions footprint: trends to 2040 & recommendations. J. Clean. Prod. *177*, 448–463.
8. Malmodin, J., and Lundén, D. (2018). The energy and carbon footprint of the global ICT and E&M sectors 2010–2015. Sustainability *10*, 3027.
9. Malmodin, J., and Lundén, D. (2018). The Electricity Consumption and Operational Carbon Emissions of ICT Network Operators 2010-2015, Technical report (Report from the KTH Centre for Sustainable Communications Stockholm).
10. NPD (2018). The Average Upgrade Cycle of a Smartphone in the U.S. Is 32 Months, According to NPD Connected Intelligence. https://www.npd.com/wps/portal/npd/us/news/press-releases/2018/the-average-upgrade-cycle-of-a-smartphone-in-the-u-s–is-32-months—according-to-npd-connected-intelligence/.
11. Masanet, E., Shehabi, A., Lei, N., Smith, S., and Koomey, J. (2020). Recalibrating global data center energy-use estimates. Science *367*, 984–986.
12. GeSI Smarter. (2020). ICT Solutions for 21st Century Challenges, Technical report, 2015. http://smarter2030.gesi.org/downloads.php.
13. Malmodin, J. (2019). Energy Consumption and Carbon Emissions of the ICT Sector (Energimyndigheten).
14. Malmodin, J. (2020). The ICT Sector's Carbon Footprint. Presentation at the techUK Conference in London Tech Week on 'decarbonising Data'. https://spark.adobe.com/page/dey6WTCZ5JKPu/.
15. Ericsson. (2020). A Quick Guide to Your Digital Carbon Footprint – Deconstructing Information and Communication Technology's Carbon Emissions, Technical report.
16. Waldrop, M.M. (2016). The chips are down for Moore's Law. Nature https://www.nature.com/news/the-chips-are-down-for-moore-s-law-1.19338.
17. Anders, S.G.A. (2020). Hypotheses for Primary Energy Use, Electricity Use and CO_2 Emissions of Global Computing and its Shares of the Total between 2020 and 2030 (WSEAS Transactions on Power Systems).
18. Simonite, T. (2016). Moore's Law is dead. Now what? Technol. Rev. https://www.technologyreview.com/s/601441/moores-law-is-dead-now-what/.
19. Court, V., and Sorrell, S. (2020). Digitalisation of goods: a systematic review of the determinants and magnitude of the impacts on energy consumption. Environ. Res. Lett. *15*, 043001.
20. Ofcom. (2017). Box Set Britain: UK's TV and Online Habits Revealed. https://www.ofcom.org.uk/about-ofcom/latest/media/media-releases/2017/box-set-britain-tv-online-habits.
21. Hilty, L., Lohmann, W., and Huang, E.M. (2011). Sustainability and ICT—an overview of the field. Notizie di Politeia *27*, 13–28.
22. Cisco. Cisco annual internet report (2018-2023). Technical report, 2020. accessed February 2020.
23. Sandvine. (2014). 1h 2014. Global Internet Phenomena Report, Technical report (Sandvine Incorporated ULC.).
24. Preist, C., Schien, D., and Blevis, E. (2016). Understanding and mitigating the effects of device and cloud service design decisions on the environmental footprint of digital infrastructure. In Proceedings of the 2016 CHI Conference on Human Factors in Computing Systems, pp. 1324–1337.
25. Sweney, M. (2020). Netflix to Slow Europe Transmissions to Avoid Broadband Overload (The Guardian). https://www.theguardian.com/media/2020/mar/19/netflix-to-slow-europe-transmissions-to-avoid-broadband-overload.
26. Rivera, M.B., Håkansson, C., Svenfelt, Å., and Finnveden, G. (2014). Including second order effects in environmental assessments of ICT. Environ. Model. Softw. *56*, 105–115, Thematic issue on Modelling and evaluating the sustainability of smart solutions.
27. Graver, B., Zhang, K.K., and Rutherford, D. (2018). CO_2 Emissions from Commercial Aviation, Technical report.

28. Lövehagen, N. (2020). What's the Real Climate Impact of Digital technology? (Ericsson). https://www.ericsson.com/en/blog/2020/2/climate-impact-of-digital-technology.

29. Apergis, I., and Apergis, N. (2019). Silver prices and solar energy production. Environ. Sci. Pollut. Res. *26*, 8525–8532.

30. IEA (2019). Solar Pv. Technical Report. https://www.iea.org/reports/tracking-power-2019/solar-pv.

31. IPCC (2018). Special Report – Global Warming of 1.5°C, Technical report. https://www.ipcc.ch/sr15/.

32. ITU. ICT (2020). Industry to Reduce Greenhouse Gas Emissions by 45 Per Cent by 2030, Technical report. https://www.itu.int/en/mediacentre/Pages/PR04-2020-ICT-industry-to-reduce-greenhouse-gas-emissions-by-45-percent-by-2030.aspx.

33. Baskerville-Muscutt, K. (2019). *Setting Science-Based Supply Chain Greenhouse-Gas Emission Targets: Aligning Corporate Climate Action with the 1.5°C Target*, Thesis.

34. International Institute of Applied Systems Analysis (2018). Welcome to the Ssp Database (Shared Socioeconomic Pathways) - Version 2.0. https://tntcat.iiasa.ac.at/SspDb/dsd?Action=htmlpage&page=40.

35. Al-Ali, A.-R., Zualkernan, I.A., Rashid, M., Gupta, R., and AliKarar, M. (2017). A smart home energy management system using IoT and big data analytics approach. IEEE Trans. Consum. Electron. *63*, 426–434.

36. Minoli, D., Sohraby, K., and Occhiogrosso, B. (2017). IoT considerations, requirements, and architectures for smart buildings—energy optimization and next-generation building management systems. IEEE Internet Things J. *4*, 269–283.

37. Sofana Reka, S., and Dragicevic, T. (2018). Future effectual role of energy delivery: a comprehensive review of Internet of Things and smart grid. Renew. Sustain. Energy Rev. *91*, 90–108.

38. Saleem, Y., Crespi, N., Rehmani, M.H., and Copeland, R. (2019). Internet of Things-aided smart grid: technologies, architectures, applications, prototypes, and future research directions. IEEE Access *7*, 62962–63003.

39. Raj, P., Raman, A., Nagaraj, D., and Duggirala, S. (2015). Big data analytics for healthcare. In High-Performance Big-Data Analytics, P. Raj, A. Raman, D. Nagaraj, and S. Duggirala, eds. (Springer), pp. 391–424.

40. Wu, J., Guo, S., Li, J., and Zeng, D. (2016). Big data meet green challenges: big data toward green applications. IEEE Syst. J. *10*, 888–900.

41. Gomes, C., Dietterich, T., Barrett, C., Conrad, J., Dilkina, B., Ermon, S., Fang, F., Farnsworth, A., Fern, A., and Fern, X. (2019). Computational sustainability: computing for a better world and a sustainable future. Commun. ACM *62*, 56–65.

42. James, J. (2019). Data as the New Oil: The Danger behind the Mantra (The Enterprises Project). https://enterprisersproject.com/article/2019/7/data-science-data-can-be-toxic.

43. IDC. (2020). IDC's Global Datasphere Forecast Shows Continued Steady Growth in the Creation and Consumption of Data. https://www.idc.com/getdoc.jsp?containerId=prUS46286020.

44. Strubell, E., Ganesh, A., and McCallum, A. (2019). Energy and policy considerations for deep learning in NLP. arXiv.

45. Biewald, L. (2019). Deep Learning and Carbon Emissions. Towards Data Science. https://towardsdatascience.com/deep-learning-and-carbon-emissions-79723d5bc86e.

46. Amodei, D., and Hernandez, D. (2019). AI and Compute. https://openai.com/blog/ai-and-compute/.

47. Dobbe, R., and Whittaker, M. (2019). AI and Climate Change: How They're Connected, and what We Can Do about it (AI Now Institute). https://medium.com/@AINowInstitute/ai-and-climate-change-how-theyre-connected-and-what-we-can-do-about-it-6aa8d0f5b32c.

48. Schwartz, R., Dodge, J., Smith, N.A., and Etzioni, O. (2019). Green AI. arXiv.

49. Jobin, A., Ienca, M., and Vayena, E. (2019). The global landscape of AI ethics guidelines. Nat. Mach. Intelligence *1*, 389–399.

50. Henderson, P., Hu, J., Romoff, J., Brunskill, E., Jurafsky, D., and Pineau, J. (2020). Towards the systematic reporting of the energy and carbon footprints of machine learning. arXiv.

51. Folding@Home. Folding@home, 2020. https://foldingathome.org/, accessed March 2020.

52. Mohanty, S.P., Choppali, U., and Kougianos, E. (2016). Everything you wanted to know about smart cities: the Internet of Things is the backbone. IEEE Consum. Electron. Mag. *5*, 60–70.

53. Elias Bibri, S. (2018). The IoT for smart sustainable cities of the future: an analytical framework for sensor-based big data applications for environmental sustainability. Sustain. Cities Soc. *38*, 230–253.

54. Govindan, K., Cheng, T.C.E., Mishra, N., and Shukla, N. (2018). Big data analytics and application for logistics and supply chain management. Transport. Res. E Log. Transport. Rev. *114*, 343–349.

55. Collier, S.E. (2017). The emerging enernet: convergence of the smart grid with the internet of things. IEEE Ind. Appl. Mag. *23*, 12–16.

56. Mylonas, G., Amaxilatis, D., Chatzigiannakis, I., Anagnostopoulos, A., and Paganelli, F. (2018). Enabling sustainability and energy awareness in schools based on IoT and real-world data. IEEE Pervasive Comput. *17*, 53–63.

57. Liu, Q., Ma, Y., Alhussein, M., Zhang, Y., and Peng, L. (2016). Green data center with IoT sensing and cloud-assisted smart temperature control system. Comput. Netw. *101*, 104–112.

58. Statista Research Department (2020). Internet of Things (IoT) Connected Devices Installed Base Worldwide from 2015 to 2025 (In Billions). https://www.statista.com/statistics/471264/iot-number-of-connected-devices-worldwide/.

59. Das, S. (2019). Global Energy Footprint of IoT Semiconductors, Technical report.

60. Kaur, N., and Sood, S.K. (2015). An energy-efficient architecture for the Internet of Things (IoT). IEEE Syst. J. *11*, 796–805.

61. Raza, U., Kulkarni, P., and Sooriyabandara, M.h. (2017). Low power wide area networks: an overview. IEEE Commun. Surv. Tutorials *19*, 855–873.

62. Karim Shaikh, F., Zeadally, S., and Exposito, E. (2015). Enabling technologies for green Internet of Things. IEEE Syst. J. *11*, 983–994.

63. Arshad, R., Zahoor, S., Shah, M.A., Wahid, A., and Yu, H. (2017). Green IoT: an investigation on energy saving practices for 2020 and beyond. IEEE Access *5*, 15667–15681.

64. Alsamhi, S.H., Ou, M., Ansari, M.S., and Meng, Q. (2019). Greening internet of things for greener and smarter cities: a survey and future prospects. Telecommun. Syst. *72*, 609–632.

65. Solanki, A., and Nayyar, A. (2019). Green Internet of Things (G-IoT): ICT technologies, principles, applications, projects, and challenges. In Handbook of Research on Big Data and the IoT (IGI Global), pp. 379–405.

66. Wilson, C., Hargreaves, T., and Hauxwell-Baldwin, R. (2017). Benefits and risks of smart home technologies. Energy Policy *103*, 72–83.

67. Enerdata. Between 10 and 20% of electricity consumption from the ICT* sector in 2030? Technical report, 2018. accessed March 2020.

68. Kouhizadeh, M., and Sarkis, J. (2018). Blockchain practices, potentials, and perspectives in greening supply chains. Sustainability *10*, 3652.

69. Saberi, S., Kouhizadeh, M., Sarkis, J., and Shen, L. (2019). Blockchain technology and its relationships to sustainable supply chain management. Int. J. Prod. Res. *57*, 2117–2135.

70. Mora, C., Rollins, R.L., Taladay, K., Kantar, M.B., Chock, M.K., Shimada, M., and Franklin, E.C. (2018). Bitcoin emissions alone could push global warming above 2°C. Nat. Clim. Change *8*, 931–933.

71. Cong, L.W., He, Z., and Li, J. (2019). Decentralized Mining in Centralized Pools, Technical Report 0898-2937 (National Bureau of Economic Research).

72. Digiconomist. (2019). Bitcoin Energy Consumption Index.

73. Stoll, C., Klaaßen, L., and Gallersdörfer, U. (2019). The carbon footprint of bitcoin. Joule *3*, 1647–1661.

74. CoinMarketCap. Global charts, 2020. https://coinmarketcap.com/charts/, accessed March 2020.

75. Alex de Vries. (2018). Bitcoin's growing energy problem. Joule *2*, 801–805.

76. Monrat, A.A., Schelén, O., and Andersson, K. (2019). A survey of blockchain from the perspectives of applications, challenges, and opportunities. IEEE Access *7*, 117134–117151.

77. Saleh, F. (2021). Blockchain without waste: Proof-of-stake. The Review of financial studies *34* (3), 1156–1190. https://academic.oup.com/rfs/article-abstract/34/3/1156/5868423.

78. Peter, H. (2019). Tackling climate change with blockchain. Nat. Clim. Change *9*, 644–645.

79. Bendiksen, C., and Gibbons, S. (2019). The Bitcoin Mining Network: Trends, Average Creation Costs, Electricity Consumption & Sources. December 2019 Update, Technical report (CoinShares Research).

80. Alex de Vries. (2019). Renewable energy will not solve Bitcoin's sustainability problem. Joule *3*, 893–898.

81. Bendiksen, C., Gibbons, S., and Lim, E. (2018). The Bitcoin Mining Network. Technical Report (CoinShares Research).

82. Koomey, J.G., Berard, S., Sanchez, M., and Wong, H. (2011). Web extra appendix: implications of historical trends in the electrical efficiency of computing. IEEE Ann. Hist. Comput. *33*, S1–S30.

83. Truby, J. (2018). Decarbonizing Bitcoin: law and policy choices for reducing the energy consumption of Blockchain technologies and digital currencies. Energy Res. Soc. Sci. *44*, 399–410.

84. Le Quéré, C., Jackson, R.B., Jones, M.W., Smith, A.J.P., Abernethy, S., Andrew, R.M., et al. (2020). Temporary reduction in daily global CO_2 emissions during the covid-19 forced confinement. Nat. Clim. Change *10*, 647–653.

85. Floridi, L., Cowls, J., Beltrametti, M., Chatila, R., Chazerand, P., Dignum, V., Luetge, C., Madelin, R., Pagallo, U., and Rossi, F. (2018). AI4people—an ethical framework for a good AI society: opportunities, risks, principles, and recommendations. Minds Mach. *28*, 689–707.

86. Delbeke, J., and Vis, P. (2016). EU Climate Policy Explained, Technical report.

87. European Commission (2018). A Clean Planet for All. A European Strategic Long-Term Vision for a Prosperous, Modern, Competitive and Climate Neutral Economy, Technical report.

88. European Commission (2019). On Artificial Intelligence - a European Approach to Excellence and Trust, Technical report. https://ec.europa.eu/info/sites/info/files/commission-white-paper-artificial-intelligence-feb2020_en.pdf.

89. European Commission (2020). Supporting the Green Transition, Technical report. https://ec.europa.eu/commission/presscorner/detail/en/fs_20_281.

90. PEDCA (Pan-European Data Centre Academy) (2015). Final Report Summary, Technical report (European Commission). https://cordis.europa.eu/project/id/320013/reporting.

91. Kayali, L., Heikkilä, M., and Delcker, J. (2020). Europe's Digital Vision, Explained (Politico). https://www.politico.eu/article/europes-digital-vision-explained/.

92. World Economic Forum (2019). A New Circular Vision for Electronics. Time for a Global Reboot, Technical report. http://www3.weforum.org/docs/WEF_A_New_Circular_Vision_for_Electronics.pdf.

93. European Commission (2020). Changing How We Produce and Consume: New Circular Economy Action Plan Shows the Way to a Climate-Neutral, Competitive Economy of Empowered Consumers. https://ec.europa.eu/commission/presscorner/detail/en/ip_20_420.

94. European Commission (2020). Eco-innovation at the Heart of European Policies (Rewards for Recycling). https://ec.europa.eu/environment/ecoap/about-eco-innovation/policies-matters/rewards-recycling_en.

95. European Commission (2019). The European Green Deal, Technical report. https://ec.europa.eu/info/sites/info/files/european-green-deal-communication_en.pdf.

96. Palmer, M. (2019). Eu Launches €2bn AI and Blockchain Fund (Sifted). https://sifted.eu/articles/eu-2bn-ai-blockchain-fund/.

97. Carrillo Pineda, A., and Faria, P.O. (2019). Towards a Science-Based Approach to Climate Neutrality in the Corporate Sector, Technical report (Science Based Targets report). https://sciencebasedtargets.org/wp-content/uploads/2019/10/Towards-a-science-based-approach-to-climate-neutrality-in-the-corporate-sector-Draft-for-comments.pdf.

98. Smith, B. (2020). Microsoft Will Be Carbon Negative by 2030 (Microsoft). https://blogs.microsoft.com/blog/2020/01/16/microsoft-will-be-carbon-negative-by-2030/.

99. Stephens, A. (2019). Net Zero: An Ambition in Need of a Definition (Carbon Trust). https://www.carbontrust.com/news-and-events/insights/net-zero-an-ambition-in-need-of-a-definition.

100. Childs, M. (2020). Does Carbon Offsetting Work? (Friends of the Earth). https://friendsoftheearth.uk/climate-change/does-carbon-offsetting-work.

101. Cames, M., Harthan, R.O., Füssler, J., Lazarus, M., Lee, C.M., Erickson, P., and Spalding-Fecher, R. (2016). How Additional Is the Clean Development Mechanism? Analysis of the Application of Current Tools and Proposed Alternatives, Technical report.

102. ITU (2018). ITU Recommendations: Itu-T I.1450 (09/2018), Technical report. https://www.itu.int/ITU-T/recommendations/rec.aspx?id=13581.

Patterns, Volume *2*

Supplemental information

The real climate and transformative impact of ICT: A critique of estimates, trends, and regulations

Charlotte Freitag, Mike Berners-Lee, Kelly Widdicks, Bran Knowles, Gordon S. Blair, and Adrian Friday

Supplementary Material: Appendix

A Methodology

A.1 Definitions

Table A.1 *Definitions for terms used throughout the report. Unless a reference is provided, these are pulled or adapted from the Cambridge Dictionary [2020] or Berners-Lee [2011].*

Term	Definition
1.5°C	1.5 degrees Celsius global warming has far fewer climate-related risks in terms of sea level rise, drought, hot weather and precipitation extremes than 2 degrees Celsius. For this reason, world leaders agreed to limit global warming to well-below 2 degrees Celsius and 'in pursuit' of 1.5 degrees Celsius at the 2015 United Nations Climate Change Conference in Paris [IPCC 2018].
2G/3G/4G/5G	Second, third, fourth and fifth generation communication technology.
Artificial Intelligence (AI)	The study of how to produce machines that have some of the qualities that the human mind has, such as the ability to understand language, recognize pictures, solve problems, and learn.
Algorithm [in the context of Blockchain/AI/Natural Language Processing]	A set of mathematical instructions or rules that, especially if given to a computer, will help to calculate an answer to a problem.
Augmented reality	Images produced by a computer and used together with a view of the real world.
Big data	Very large sets of data that are produced by people using the internet, and that can only be stored, understood, and used with the help of special tools and methods.
Bitcoin	A type of cryptocurrency.
Blockchain	A decentralised algorithm. In the context of cryptocurrencies: a system used to make a digital record of all the occasions a cryptocurrency is bought or sold, and that is constantly growing as more blocks are added.
Cap and trading scheme (for carbon)	A cap is set on the total amount of certain GHGs that can be emitted. Within this cap, companies buy or receive emission allowances, which they can trade with one another. At the end of the year, a company must give up enough allowances that cover all its emissions or face a fine. Any spare allowances can be kept to cover future emissions or sold to other companies.
Carbon	A shorthand for all the different global-warming greenhouse gases.

Carbon footprint	A best estimate for the full climate change impact of something, including all greenhouse gases, expressed in carbon dioxide equivalent (the amount of carbon dioxide that would have the same impact as the specific greenhouse gas associated with a thing); the central climate change metric.
Carbon intensity	The amount of greenhouse gas emissions associated with an activity.
Carbon negative	The process by which an activity sequesters more greenhouse gas emissions than are emitted through said activity.
Carbon neutral	Releasing no net greenhouse gas emissions into the atmosphere. Typically achieved by reducing emissions and using offsets to counterbalance any emissions generated.
Climate change	Changes in the earth's weather, including changes in temperature, wind patterns, and rainfall, especially the increase in the temperature of the earth's atmosphere that is caused by the increase of particular gases, especially carbon dioxide.
Cloud computing	The use of services, computer programs, etc. that are on the internet rather than ones that you buy and put on your computer.
CO_2	Carbon dioxide, the most common greenhouse gas.
CO_2e	Carbon dioxide equivalent. Different greenhouse gases have different global warming potentials. CO_2e expresses the climate change impact of all greenhouse gases emitted in association with an activity as the amount of carbon dioxide that would have the same climate change impact.
Cryptocurrency	A digital currency produced by a public network, rather than any government, that uses cryptography to make sure payments are sent and received safely.
Data centre	A place where a number of computers that contain large amounts of information can be kept safely.
Data science	The use of scientific methods to obtain useful information from computer data, especially large amounts of data.
Data traffic/Internet traffic	The activity of data and messages passing through an online communication system or the number of visits to a particular website.
Decarbonising	Reducing the carbon footprint of an activity.
Dematerialisation	Reducing the amount of material needed to produce a product.
Downstream traffic	Data traffic that is moving in a downstream direction (i.e. being downloaded).
Economy-wide impacts of ICT	The impact the ICT industry has on other industries, for example through allowing for efficiencies, providing additional products and/or replacing more traditional technologies, but also allowing intensified activity or growth in other areas of the economy. The effect can be both to

	increase or decrease impact and those other industries. Differentiated from ICT's impact within the ICT industry. The net effect of ICT depends on the impact it has in both areas and their balance.
Emissions	A shorthand for greenhouse gas emissions.
Entertainment and Media (E&M) sector	A sector category used by Malmodin and colleagues; it covers TV, consumer electronics (such as cameras and audio systems in a car and portable GPS) and print media.
Environmentally Extended Input Output (EEIO) analysis	A "top-down" approach for estimating life cycle emissions, capable of capturing impacts from the entire supply chain. See Appendix F for details.
Embedded device/system	A computer system that does a particular task inside a machine or larger electrical system, or physical object.
Embodied carbon/emissions	The greenhouse gas emissions released from the extraction of raw materials required, the manufacturing process and transport and distribution of a product. It includes a share of all the activities required to take goods and services at the point of sale, but excludes the product use phase. It can be from cradle to factory gate, from cradle to site of use or from cradle to grave – in the latter case, end of life emissions are included. In this report, we assume cradle to point of sale unless otherwise stated.
Enablement	The avoidance of emissions in the wider economy through ICT applications, including through improved efficiency.
End of life emissions (see lifecycle stages)	Emissions after disposal of a product, after the end of the use phase.
Energy footprint	The amount of energy used by a product, activity or industry.
Exponential growth	A rate of increase which becomes quicker and quicker as the thing that increases becomes larger.
Fossil fuel	Fuels, such as gas, coal, and oil, that were formed underground from plant and animal remains millions of years ago.
GB (Gigabytes)	A unit of computer information consisting of 1,000,000,000 bytes.
Greenhouse gas (GHG) emissions, or emissions for short	Gases that contribute to global warming, including carbon dioxide (CO_2), methane (CH4), nitrous oxide (N2O) and fluorinated gases.
ICT's own impact	The impact the ICT industry has in terms of its energy use or GHG emissions through the entire lifecycle of its products, including their manufacture, operation and disposal. Differentiated from the effect ICT has on other industries, that is, economy-wide impacts.
Information Communication Technology (ICT)	The use of computers and other electronic equipment and systems to collect, store, use, and send data electronically.
Internet of Things (IoT)	Objects with computing devices in them that are able to connect to each other and exchange data using the internet.

Life Cycle Analysis (LCA)	The detailed study of the series of changes that a product, process, activity, etc. goes through during its existence and the resulting environmental impact.
Lifecycle stages (Material extraction, manufacturing, transport, use phase, end of life)	The stages of resource use and environmental releases associated with an industrial system from the extraction of raw materials from the Earth and the production and distribution, through the use, and reuse, and final disposal of a product.
Machine-to-Machine (M2M) communication	The act of sending data between machines or computers.
Machine Learning (ML)	The process of computers changing the way they carry out tasks by learning from new data, without a human being needing to give instructions in the form of a program.
Moore's Law	The observation by Gordon Moore of Intel Corporation that the cost of a computer chip for a particular amount of processing power will continue to fall by half every two years.
Natural Language Processing (NLP)	A field of Artificial Intelligence that gives the machines the ability to read, understand and derive meaning from human languages [Lopez Yse 2019].
Net zero	Having no net climate change impact through greenhouse emissions in a company's value chain. This is achieved by reducing greenhouse gas emissions in the value chain and removing the remaining emissions through additional carbon removals.
Network	A number of computers that are connected together so that they can share information.
Offset	A mechanism to negative a certain amount of GHG emissions either through avoiding emissions elsewhere (e.g. through protecting a forest from logging) or removing emissions form the atmosphere (for example through natural carbon sequestration, such as peatland restoration or reforestation projects). There is debate whether avoided emissions should count as additional and whether they are permanent.
Operational emissions	See use phase emissions.
Operator activities	Activities by operators of manufacturing plants, data centres and networks, such as office heating and lighting, business travel, maintenance of equipment, a share of which should be allocated to the lifecycle emissions of equipment using data centre and network services.
Proof of Work / Proof of Stake	Types of Blockchain consensus algorithms which are processes in computer science used to achieve agreement on a single data value among distributed systems [CoinBundle Team 2018].
Rebound effect	The way in which micro-actions can be nullified by counter balancing adjustments elsewhere in the global system. Often used to refer to the way increased energy efficiency leads to more energy usage overall.

Renewable energy	Energy that is produced using the sun, wind, etc., or from crops, rather than using fuels such as oil or coal.
Router	A piece of electronic equipment that connects computer networks to each other, and sends information between networks.
Scope 1 emissions	Direct emissions from burning of fossil fuels on site (includes company facilities and vehicles)
Scope 2 emissions	Indirect emissions from purchased electricity and gas.
Scope 3 emissions	All other indirect emissions in a company's value chain; including upstream emissions in the supply chain (e.g. emissions from purchased goods and services, transportation of these goods to the company, use of leased assets such as offices or data centres, business travel and employee commuting) and downstream emissions (from transportation, distribution, use and end of life treatment of sold products, investments and leased assets). Scope 3 emissions form the majority of a company's emissions.
Semiconductor	A material, such as silicon, that allows electricity to move through it more easily when its temperature increases, or an electronic device made from this material.
Server	A central computer from which other computers get information.
Set top box	An electronic device that makes it possible to watch digital broadcasts on ordinary televisions.
Smart technology [e.g. smart grids/cities/ logistics/agriculture]	An object/city/process etc. that is internet-connected and therefore able to make intelligent decisions.
Supply chain emissions	Emissions that occur upstream of a company's own operations, including emissions from purchased goods and services, transportation of these goods to the company, use of leased assets such as offices or data centres, business travel and employee commuting. See upstream scope 3 emissions.
Truncation error	In the context of carbon accounting, truncation error describes the omission of some proportion of the total carbon footprint by LCAs because this approach is unable to track all the supply chain pathways associated with a thing. That means, they disregard or *truncate* the pathways that individually only contribute a small share of the total, often set to less than 1%, even though these can make up sizeable share of the total if they are all added up. See Appendix F for more detail.
Upstream traffic	Data traffic that is moving in an upstream direction (i.e. being uploaded).
Use phase/Operational emissions	Emissions associated with the use of a product, mainly from energy use and maintenance.
Value chain emissions	Emissions occurring in a company's value chain, both upstream in the supply chain (from manufacture and

	transport), from its operations and downstream (from product use by customers).
Video-on-demand services	Services (e.g. Netflix, Amazon Prime, BBC iPlayer) that provide a system for watching films or recorded programmes on the internet or television at any time.
Virtual reality	A set of images and sounds, produced by a computer, that seem to represent a place or a situation that a person can take part in.
Virtualisation/ Server virtualisation	The process of changing something that exists in a real form into a virtual version.

A.2 Abbreviations

Table A.2 Abbreviations used throughout the report.

Abbreviation	Term
AI	Artificial Intelligence
A&E	Andrae and Edler (2015)
BEIS	Department for Business, Energy and Industrial Strategy
B&E	Bekhir and Elmeligi (2018)
CEH	UK Centre for Ecology and Hydrology
CRT	Cathode Ray Tube
DAC	Direct Air Capture
EC	European Commission
EEIO	Environmentally Extended Input Output
GeSI	Global e-Sustainability Initiative
GHG	Greenhouse gas emissions
GSMA	Global System for Mobile Communications Association
ICT	Information and Communication Technology
IoT	Internet of Things
ITU	International Telecommunication Union
LCA	Life Cycle Analysis
LED	Light-Emitting Diode
M&L	Malmodin and Lundén (2018)
PC	Personal Computer
PPA	Power Purchasing Agreements
REGO	Renewable Energy Guarantees of Origin
SBTi	Science Based Targets initiative
SWC	Small World Consulting
TV	Television
UN	United Nations

A.3 Units

Emissions are measured in kilograms (kg), tons (t), kilotons (kt), megatons (Mt) and gigatons (Gt). GHG emissions, for example, are often expressed in $MtCO_2e$ or $GtCO_2e$.

1 Gt = 1,000 Mt; 1 Mt = 1,000 kt; 1 kt = 1,000 t; 1 t = 1,000 kg

Energy consumption is measured in watt-hour (Wh), kilowatt-hour (kWh), megawatt -hour (MWh), gigawatt -hour (GWh) and terawatt -hour (TWh).

1 TWh = 1,000 GWh; 1 GWh = 1,000 MWh; 1 MWh = 1,000 kWh; 1 kWh = 1,000 Wh

A.4 Scope

For the purposes of this report, we have adopted a broad definition of ICT to include all types of data centres, networks and user devices used for processing, storing, sending and receiving digital information. This includes data centres of all scales (i.e. servers run by companies in cupboard up to large data centres), all major types of networks (telephony, mobile and broadband data, TV), and a wide range of digital end user devices, such as PCs, laptops, tablets, mobile and fixed phones, TVs, displays and gaming equipment (see Appendix B.2.1). We included all stages of equipment lifecycle, from the extraction of the raw materials, manufacture, transport and use to end of life. For networks and data centres, we included infrastructure (such as the construction and running of the building housing the servers, including cooling, and the digging down of network cable tracks) and operator activities (e.g. business travel, office heating and lighting etc.).

A.5 Method

For the literature review, we built on our collective knowledge of the literature and carried out additional literature searches using Google Scholar, the ACM Digital Library and the citation information from relevant papers. Note that this was not a systematic literature review. For the main review of ICT's carbon impact (Section 2), we included peer-reviewed journal articles published from 2015 onwards with the key words outlined below. These key words were also drawn upon to facilitate our analysis of the trends in ICT and their environmental implications (Section 3). For our policy analysis (Section 4.1), we focused solely on European Commission documents and websites. Our analysis of industry pledges (Section 4.2) draw on a survey of annual reports, blog posts and web pages for 18 major ICT companies (Microsoft, Sky, Vodafone, Apple, Amazon, Netflix, Facebook, Tesla, Google, Samsung, Ericsson, Spotify, Huawei, Cisco, Sony, Nintendo, Intel and IBM).

A.5.1 Key words

- Sustainability
- Energy consumption/ energy
- Carbon emissions
- GHG (Greenhouse gas) emissions
- LCA (Life-Cycle Analysis)
- Efficiency
- ICT/ IT (information technology)/ digital technologies

- User devices
- Internet traffic/ Internet
- Data traffic
- Data centres/ data centers
- Communication networks/ networks
- Big data
- AI (Artificial Intelligence)
- Machine learning
- Data science
- IoT (Internet of Things)
- Smart (home, grid, city)
- Cryptocurrencies
- Blockchain
- Bitcoin
- Video streaming/ video
- YouTube
- Video-on-demand
- TV/ television
- Cloud computing/ services
- Jevons Paradox
- Rebound effect

A.5.2 Selection of key papers

Articles were selected guided by the following questions:

- Does the paper focus on the energy or carbon impacts of ICT, its major components (e.g. data centres, networks), or its major application areas (e.g. AI, IoT)?
- Does the paper focus on the impact ICT has on energy or carbon consumption in other sectors?

A.5.3 Consultation with key experts

In addition to this, we consulted with the following leading experts based on their extensive knowledge on the carbon impacts of ICT through video conference calls:

- Dr. Lotfi Belkhir (Associate Professor at W Booth School of Engineering Practice and Technology, McMaster University)
- Dr. Anders Andrae (Senior Expert at Huawei Technologies)
- Jens Malmodin (Senior Specialist at Ericsson)
- Dr. Peter Garraghan (Reader in Distributed Systems at the School of Computing and Communications, Lancaster University)
- Livia Cabernard (PhD student at the Institute of Science, Technology and Policy, ETH Zurich)
- Prof. Chris Preist (Professor of Sustainability and Computer Systems at University of Bristol)

We discussed their research in relation to ICT's carbon footprint, their opinion of other prominent studies, their response to criticism from the other experts, their view on the

future of ICT's emissions and on the trends posing risks and opportunities for ICT's impact on climate change.

A.5.4 Other sources of information

For this report, we drew on research by Small World Consulting (SWC) Ltd. into sector emissions to adjust estimates by the key studies in Section 2 for truncation error.

SWC developed an environmentally extended input output (EEIO) model (described in detail by Berners-Lee et al. [2011] and Kennelly et al. [2019]) that uses data from the Office of National Statistics on the expenses and GHG emissions from 105 industries in the UK to calculate the carbon intensity per Pound spent. This allows us to model carbon flows in the UK economy and the upstream scope 3 emissions of an industry in its supply chain, by tracking the economic activity stimulated by each sector in other sectors. In contrast to LCAs, SWC's EEIO model tracks 100% of all supply chains associated with a sector. It can be used to estimate the truncation error of LCAs for a particular sector; that is, the percentage of the total emissions that is typically omitted by an LCA. We note that SWC's EEIO model is based on UK emissions data which are not representative of other economies, yet it provides a good–enough estimate to help understand the potential truncation error incurred by LCA estimates (Appendix F).

For manufacture of ICT equipment, these omissions include radiative forcing, manufacture of buildings and machines, of mining equipment and of transport vehicles and other operator activities and overheads associated with the manufacture of a product. We also know that most LCAs do not include pathways that contribute less than 1% of the total carbon footprint. In total, these excluded pathways make up 40% of the total embodied carbon.

For the operation of ICT equipment, electricity is the most important source of GHG emissions. Based on a hybridised EEIO-LCA model SWC developed from scope 1 and 2 emissions data from BEIS [Department for Business, Energy and Industrial Strategy 2019] and the IEA [2019], SWC estimates that the carbon intensity of global average grid electricity in 2018 was 0.63 kgCO_2e/kWh or MtCO_2e/TWh. The carbon intensity factor for electricity used in most LCAs includes emissions from electricity generation and transmission and distribution losses, but not extraction and transportation of fuel to the plant, the manufacture of equipment used in these processes and operator activities. Based on this, we estimate that LCAs omit 18% of the use phase carbon. Our truncation error mark-up applied to the key studies reviewed here is based on the difference between the specific electricity intensity factor they report and SWC's factor of 0.63 kgCO_2e/kWh.

All percentages out of global GHG emissions are based on a total of 57.9 GtCO_2e in 2020. This is based on 55.6 GtCO_2e GHG emissions, including land use change, in 2018 [Olivier and Peters 2019], assuming the growth rate of 2% in 2018 applies to 2019 and 2020. Note that this extrapolation did not consider the impact of Covid-19 on emissions.

A.6 Limitations

This report is not based on a systematic literature review but rather built on our own knowledge of the sector alongside strategic literature searches aimed at covering the

main studies in the field. We have focused on critically analysing the main arguments surrounding the ICT sector's environmental footprint and trends. We have not scrutinised reports about the impact of individual components of ICT (e.g. the carbon impacts of servers alone) or covered the full breadth of research papers within ICT on IoT, Blockchain and AI that do not take an environmental position (e.g. instead focus solely on health, finance, etc.). We have limited our discussion of impacts on the wider economy to the ICT sector's potential to enable efficiencies or drive emissions in other sectors; a full, economy-wide assessment of ICT's environmental impacts globally was deemed out of scope for this study. We are confident, however, that we have captured the main academic debates and the most relevant non-academic publications on the climate change impact of the ICT sector as a whole and the impacts of prominent ICT trends going forward. We call for future work to fully assess the Enablement and Global Rebounds narratives (see Figure 5) on the world's economy.

Carbon accounting is a rather imprecise science due to the complexity of the supply chain emissions pathways and issues with how to allocate emissions to a particular product, activity or sector. For each carbon footprint calculation, there is a margin of error. The uncertainty increases even further for projections of future emissions, as these are influenced by the actions of companies, policy makers, individual users and unforeseen events like natural catastrophe and pandemics. There are several unknowns including what changes future innovations might bring or the carbon footprint of activities which are largely undocumented (e.g. the dark web). The carbon footprint of some of the emerging ICT trends are also difficult to calculate, e.g. IoT and Blockchain due to their hidden and distributed nature.

We have tried to make this uncertainty clear throughout the report. The carbon footprints calculations in this report serve as approximations indicating the order of magnitude and important trends in emissions that can guide decision-making about the effects that different courses of action could have on climate change. Furthermore, the lack of coherent standards for carbon accounting leads to different approaches, scopes and assumptions being used by different studies. We have attempted to make these explicit and compare the different methodologies used by the key studies reviewed in Section 2.2.

For reasons outlined above, emission estimates are more uncertain than estimates of electricity consumption. Nevertheless, an assessment of ICT's climate change impact needs to focus on GHG emissions rather than electricity consumption alone because it is emissions that ultimately drive climate change, and electricity consumption itself does not capture the impact of factors such as energy source mix and emissions in the energy generation supply chain. Since most studies focus on ICT's energy consumption, we felt that we could most usefully contribute to the scientific debate by applying our expertise in supply chain emission accounting to clarify some of the complexities around the emission footprint from energy use and other sources of GHG.

B Estimates of ICT Emissions

B.1 Historical Estimates of ICT's GHG footprint

Table B.1 *Historical estimates of ICT's GHG footprint. Unless otherwise stated, all estimates include embodied (based on LCAs) and use phase GHG emissions.*
**Based on 670 TWh in 2007 and 930 TWh in 2012 [Lannoo et al. 2013] and 0.68 $MtCO_2e$/TWh (SWC estimate).*
***Based on 655 TWh in 2007 and 909 TWh in 2012 [Van Heddeghem et al. 2014] and 0.68 $MtCO_2e$/TWh (SWC estimate).*

Study	Year	$MtCO_{2e}$	Scope for emissions
Gartner [2007]	2007	620	CO_2 emissions only; use phase and emissions for phones, PCs, printers, data centres and networks
GeSI [2008]	2002	530	Desktop PCs and laptops and PC peripherals (monitors, printers), data centres, telecoms networks and devices
	2007	830	
	2020	1430	
Malmodin et al. [2010]	2007	1,150	Phones, PCs, modems, networks and data centres (630 $MtCO_2e$); TVs, TV peripherals and TV networks (390 $MtCO_2e$); other E&M equipment, including audio devices, cameras and gaming consoles (130 $MtCO_2e$)
GeSI [2012]	2011	910	PCs (desktops, laptops), mobile devices (tablets, smartphones, regular mobile phones), and peripherals (external monitors, printers, set-top boxes, routers, IPTV boxes); fixed and wireless networks (excluding local WiFi networks), data centres (servers, storage and cooling)
	2020	1270	
Lannoo et al. [2013]*	2007	454	Emissions from electricity and use phase only; computers, data centres, networks
	2012	630	
Malmodin et al. [2013]	2020	2,200	Phones (fixed, mobile), PCs (desktops, laptops), modems, networks and data centres (1,100 $MtCO_2e$); TVs, TV networks and TV peripherals (1,100 $MtCO_2e$); other E&M equipment, including audio devices, cameras and gaming consoles (420 $MtCO_2e$)
Van Heddeghem et al. [2014]**	2007	444	Emissions are use phase electricity only; desktops, laptops, monitors, networks and data centres
	2012	616	
Malmodin [2019]	2010	720	Phones (fixed, smartphones, other mobile), tablets, PCs (desktops, laptops), displays, modems, some IoT, networks and data centres

B.2 Detailed Review of the Key Studies

This report focuses on reviewing peer-reviewed studies by three main research groups published from 2015 that estimate ICT's carbon footprint from 2015 onward. Here, we

include a summary of the studies scope and assumptions (B.2.1), then follow with an overview of estimates (B.2.2) and a detailed review of relevant studies by researchers around Andrae (B.2.3), Belkhir (B.2.4) and Malmodin (B.2.5).

B.2.1 Overview of scope and methodological differences

Studies on the energy and carbon footprint of IT can be classed as either bottom-up (based on LCAs, energy use reports for certain devices and company reports, combined with data on the number of devices produced and used in a given year and the number of network subscriptions), or top-down (based on national or global statistics and input-output analysis). The latter is often difficult to obtain. Most studies use a bottom-up approach in combination with some top-down data, for example combining LCAs for user devices with global statistics for data traffic, such as from Cisco. Using a combined method is probably the best approach to assess emissions accurately.

The studies reviewed for this study use different methodological approaches. Some only include emissions from electricity (e.g. A&E), presenting a more limited picture, while others also include other sources of GHGs (e.g. B&E; Malmodin's research), such as fossil fuel backup power for data centres, fuels used by vehicles and other sources of emissions in the process of mining.

All the key studies include use phase emissions but studies vary as to the other lifecycle stages considered. On one end of the spectrum, in addition to the use phase, A&E only include production energy, just one aspect of embodied emissions. On the other end, Malmodin's research includes end of life emissions, that is, the emissions associated with waste management. The stages of the equipment lifecycle covered by the different studies in this review as well as the scope and assumptions applied are summarised in Table B.*2*.

Table B.2 *Scope matrix for studies included in this review. T&D = Transmission and distribution losses in electricity grids. Note that Malmodin includes some 'Other digital technologies or trends', specifically: wearables such as smart watches and fitness trackers, smart energy meters, control units, surveillance cameras, public displays, payment terminals and the internet-connected communication device in vending machines.*
**Included in the E&M sector estimates, not ICT estimates.*

Component of ICT sector	Andrae and Edler (2015)	Belkhir and Elmeligi (2018)	Malmodin and Lundén (2018)
**User devices**			
Smartphones	✓	✓	✓
Nonsmart mobile phones	✓	✕	✓
Fixed phones	✕	✕	✓
Tablets	✓	✓	✓
Phablets	✓	✓	✕
Laptops/Notebooks	✓	✓	✓
Desktop PCs	✓	✓	✓
Displays	✓	✓	✓
Computer peripherals (e.g. mouse and keyboard)	✕	✕	✓
Projectors	✕	✕	✓*

Cameras	✗	✗	✓*
Home media players/audio systems/traditional speakers	✓	✗	✓*
Portable media players, e.g. iPods	✗	✗	✓*
Smart speakers	✗	✗	✗
Smart watches/fitness trackers	✗	✗	✓*
Headphones/Earphones	✗	✗	✓*
Game consoles	✓	✗	✓*
Arcade game machines	✗	✗	✓*
Customer premises equipment (routers, modems)	✓	✓	✓
**Networks**			
Fixed telephony	✓	✓	✓
Mobile	✓	✓	✓
Fixed access wired	✓	✓	✓
Fixed access WiFi	✓	✓	✓
Enterprise networks	✗	✓	✓
Lower power, lower bandwidth device networks for IoT	✗	✗	✗
**Data centres**			
Servers	✓	✓	✓
Buildings that house servers	✗	✓	✓
Cooling	✓	✓	✓
Backup power supplies	✗	✓	✓
Operator activities, such as offices, business travel, maintenance of equipment	✗	✗	✓
**TVs, TV peripherals and TV networks**			
TVs	✓	✗	Yes*
Set top boxes	✓	✗	Yes*
Aerials	✗	✗	-
Satellite dishes	✗	✗	Yes*
DVD/BD players	✓	✗	Yes*
TV networks	✗	✗	Yes*
>>Cable	✗	✗	Yes*
>>Satellite	✗	✗	Yes*
>>DTT	✗	✗	Yes*
**Other digital technologies or trends**			
Cryptocurrencies/Blockchain	✗	✗	✗

AI/Machine Learning	✗	✗	✓
IoT	✗	✗	✓ (some)
Satellites	✗	✗	✗
Radio (device+networks)	✗	✗	✗
Embedded devices, e.g. sensors for smart cities, smart home tech, M2M communication	✗	✗	✓ (some)
Private internet, e.g. for military purposes	✗	✗	✗
**Trends considered for future projections**			
Blockchain	✗	✗	✗
Artificial Intelligence/Deep learning/Machine Learning	✓	✗	✗
IoT	✓	✗	✓
Video	✓	✗	✗
**Assumptions**			
Electricity carbon intensity ($kgCO_2e/kWh$)	Varies by scenario; 0.61 in 2015; 0.6-0.61 in 2020; 0.55-0.65 in 2030	0.5	0.6
Aspects of electricity likely covered (inferred from number)	generation, well-to-tank and T&D losses	generation only	generation, well-to-tank and T&D losses
Use phase included	Yes	Yes	Yes
Embodied included (based on LCAs)	Yes	Yes	Yes
Embodied carbon included	Production electricity only; no transport or end of life considered and no other sources of GHG other than electricity	Material extraction and manufacturing energy, not transport and end of life	Material acquisition, parts and component production and assembly, transport and end of life

B.2.2 Estimates for ICT's GHG Emissions in 2015 and 2020

Table B.**3** below estimates summaries by the key studies for 2015 and 2020.

Table B.3 *Estimates of GHG emissions from the ICT sector in a) 2015 and b) 2020.*

GHG emissions from ICT in 2015 ($MtCO_2e$)	User devices	Data centres	Networks	Total without TV	TVs	Total with TV
M&L 2015	395	160	180	733	420	1,153
B&E 2015 minimum	290	281	204	775	-	N/A
B&E 2015 maximum	485	281	204	971	-	N/A
B&E 2015 average (calculated)	388	281	204	873	-	N/A
A&E 2015 best case	186	213	190	589	329	917
A&E 2015 expected case	324	441	287	1,052	463	1,515
A&E 2015 worst case	514	582	454	1,550	706	2,257
**GHG emissions from ICT in 2020 ($MtCO_2e$)**	**User devices**	**Data centres**	**Networks**	**Total without TV**	**TVs**	**Total with TV**
Malmodin 2020	392	127	168	690	400	1,090
B&E 2020 minimum	343	495	269	1,107	-	N/A
B&E 2020 maximum	542	495	269	1,306	-	N/A
B&E 2020 average	443	495	269	1,206	-	N/A
A&E 2020 Best case	201	216	206	623	264	887
A&E 2020 Expected case	369	448	631	1,448	413	1,860
A&E 2020 Worst case	790	1,001	1,251	3,042	711	3,634

The studies vary in the scope with B&E only including user devices, data centres and networks, A&E including TVs and M&L including other consumer electronics such as cameras and audio systems in a car and portable GPS. In order to make estimates more comparable, we have brought them to the same 'system boundary', by adding Malmodin's estimate for the E&M sector (400 $MtCO_2e$; excluding print media) to B&E's and A&E's estimates (after subtracting emissions from TV from A&E's total estimates, using information provided in their supplementary information). The results are shown below. Considering that Andrae judges his Best case to be most realistic for 2020

[personal communication], the most likely range is 1.0-1.7 $GtCO_2e$ for ICT, TVs and other consumer electronics in 2020; this is 1.8-2.9% of global GHG emissions.

Table B.4 *Estimates of GHG emissions from the ICT sector in 2020 after adjusting for scope to include TVs and other consumer electronics.*

	$MtCO_2e$	Share of total GHG emissions
Malmodin 2020	1,090	1.9%
B&E 2020 Minimum	1,507	2.6%
B&E 2020 Maximum	1,706	2.9%
A&E 2020 Best case	1,023	1.8%
A&E 2020 Expected case	1,848	3.2%
A&E 2020 Worst case	3,442	5.9%

B.2.3 Research by Andrae and colleagues

Approach

Andrae and Edler [2015], from here on A&E, used a hybrid top-down bottom-up approach to model the production and use phase electricity use of user devices, networks, data centres and TVs between 2010 and 2030. User device emissions are modelled bottom-up from predicted production numbers and estimates for production and use phase energy use derived from LCAs. Estimates for the use phase electricity consumption of data centre and network are based on top-down data traffic trends based on Cisco data and estimates for electricity per data unit from the literature, while estimates for production electricity use are based on a fixed share of total electricity use by networks and data centres (5%, 10% and 15% for best, expected and worst case, respectively) – a method that seems somewhat imprecise. Their model also considers changes in energy efficiency (1% annually in the worst case, 3% in the expected case and 5% in the best case) and in electricity carbon intensity based on projected share of renewables which vary by year and scenario.

Findings

A&E's estimates for 2030 vary by a factor of 13, yet all scenarios show an increase relative to 2020 (see Figure B.1 Andrae and Edler's projections for GHG emissions from ICT by year.). While a growth trend in data traffic underlies the increase in total emissions, the large uncertainty in the size of this trend leads to the wide range of estimates. While the footprint of user devices is becoming less important, partly due to a shift from desktops and laptops to smaller devices like smartphones, and networks and data centres will contribute an increasing share of the total emissions over the next decade, due to the increase in data traffic. A&E argue that this growth in data traffic is driven by the popularity of video streaming, especially over mobile data, and emerging new data-heavy technologies, such as cloud computing. In more recent papers [2019a, 2019b, 2019c], Andrae also argues that AI and deep learning, IoT, Blockchain, virtual and augmented reality, facial recognition, and the rollout of 5G could lead to an explosion of data traffic over the next decade. In addition, IoT devices could increase the production footprint of ICT.

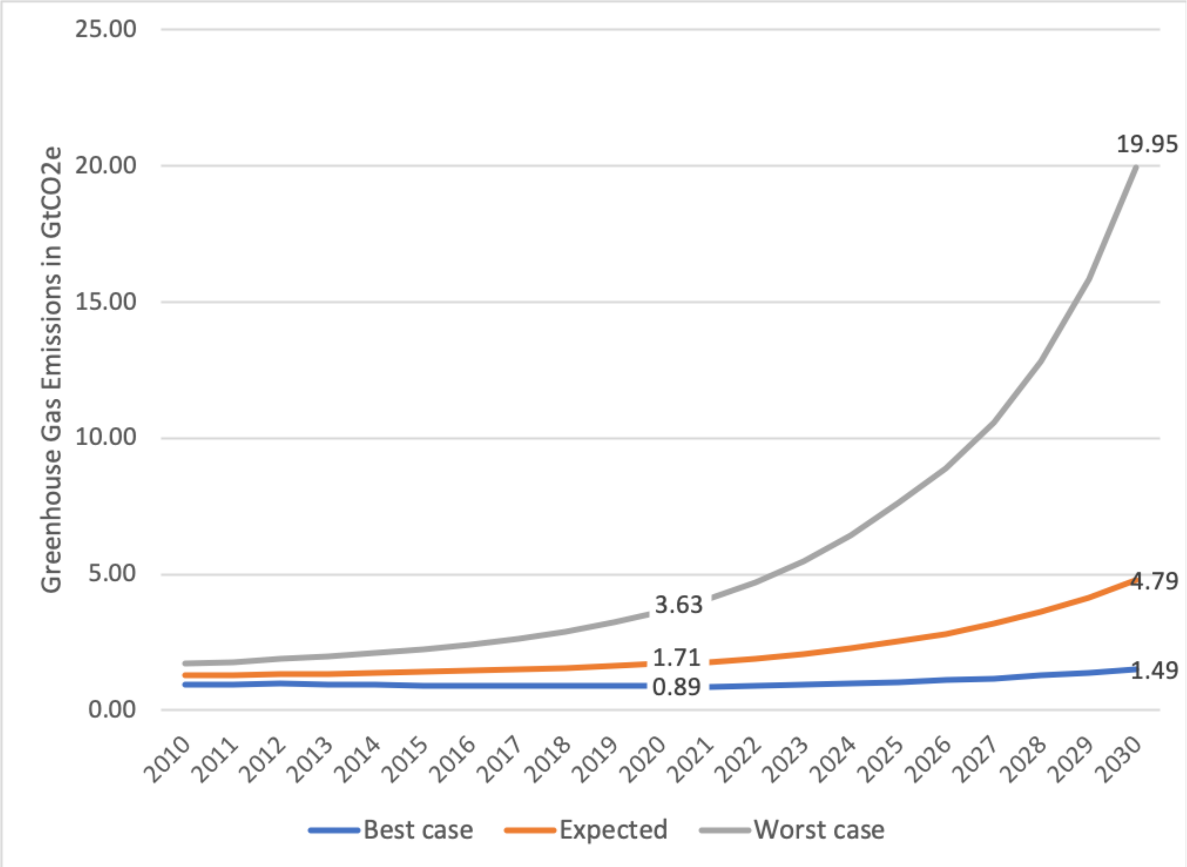

Figure B.1 *Andrae and Edler's projections for GHG emissions from ICT by year.*

A&E's worst case paints a dark picture and has been criticised as unrealistic by B&E and Malmodin [personal communication]. In personal communication, Andrae noted that A&E's study overestimated the carbon footprint of fixed wired and WiFi networks quite much even in the best case but underestimated mobile networks. Thus, for wireless and fixed access, the best case is the most relevant while for mobile, the expected case is the most likely. In 2019c, Andrae also notes that he overestimated production electricity of networks and data centres and that the ratio of production electricity to use phase electricity should be 2% instead of 5% used for the best case. Andrae's revised estimates for 2020 and 2030 (see Table B.**5**) are close to the best case scenario, partly thanks to increasing awareness of ICT's large energy footprint. But while ICT is saving electricity, those savings are used for further expansion, such as in cloud computing. If the trends discussed above take off unexpectedly, Andrae believes that data centre electricity use could be more than 4000 TWh in 2030 [personal communication].

Table B.5 *Andrae's revised estimates for 2020 and 2030 [personal communication], based on Andrae [2019b, 2019c, 2020]. Andrae uses an electricity carbon intensity of 0.55 $MtCO_2e$/TWh in 2020 and 0.54 $MtCO_2e$/TWh in 2030. Consumer devices including WiFi modems and TVs. *Use phase only*

Year	2020			2030		
**Metric**	**TWh**	**Range of $MtCO_2e$**	**Avg. $MtCO_2e$**	**TWh**	**Range of $MtCO_2e$**	**Avg. $MtCO_2e$**
Consumer devices*	600-1000	330-550	440	400-1000	216-540	378
Networks*	200-270	110-149	129	330-870	178-470	324
Data centres*	290-300	160-165	162	600-1000	324-540	432
Production of the above	250-380	138-209	173	180-300	97-162	130
	**1300-1900**	**715-1045**	**880**	**1500-3200**	**810-1728**	**1269**

In summary, Andrae believes that ICT's carbon footprint will continue to grow if not for major breakthroughs, albeit at a lower rate for the next few years than previously estimated. While the absolute total will increase, ICT's share of global electricity might stay stable if there are interventions or breakthroughs but this is unlikely under business as usual. Emissions might only reduce if data centres and production facilities are run entirely on renewable energy and if data intensity grows slower than expected [Andrae 2020]. Andrae [2020] notes that there are several potential 'engineering tricks' that might uphold efficiency gains even though Moore's Law has ended, such as decreasing semiconductor use stage power and nanophotonics, but it is unclear to what extent they have already been exploited.

Limitations and Criticism

One important difference to the other studies reviewed here is that only production electricity is included but not transport and material extraction, nor other sources of GHG other than electricity. Using the example of smartphones, they estimate between 18.8 (best case) and 37.5 (worst case) $kgCO_2e$ in 2010 but with efficiency gains, this decreases to 14.1-35.0 $kgCO_2e$ in 2015 and 10.6-33.0 $kgCO_2e$ in 2020. The assumption of decreasing embodied emissions for devices is problematic because smartphones are getting bigger and computationally more powerful, counteracting efficiency gains and greening of the electricity grid. In comparison, M&L estimated 45 $kgCO_2e$ for the average smartphone embodied footprint and B&E estimated 24.5-45.3kg. Thus, the study likely underestimates the embodied carbon footprint of ICT.

A&E's study has also been criticised by B&E for using a variety of device lifetimes in their scenarios that are not based on the published literature (e.g. 1, 2 and 3 years for smartphones, nonsmartphones, phablets and tablets for worst, expected and best case, respectively), thereby increasing the variance of embodied emission estimates.

In their calculations, A&E use the same number of device units for both the production and operational energy. In a sense, they are calculating how much electricity was used to produce all devices in use in a given year, regardless of when these devices were produced. That assumption is flawed as the same energy efficiency assumptions are applied to all devices used in a given year, even though older devices produced in earlier years will have not benefitted from these efficiency gains. Their figures might therefore underestimate the production energy for user devices further.

However, this paper has to be credited as the most transparent of all the papers reviewed, as the authors lay out clearly their assumptions and calculations in the supplementary information, broken down to the individual device and network type for every year between 2010 and 2030. It is also well-grounded in the previous published literature. The biggest criticism is probably the wide range of projections which leads to a difference between the best and worst case by a factor of 13.4; that said, a high degree of uncertainty does exist especially in such a rapidly developing sector as ICT.

B.2.4 Research by Belkhir and colleagues

Approach

Belkhir and Elmeligi (2018), from here on B&E, used a bottom-up approach for user devices and a top-down approach for data centres and networks and for total projections beyond 2020. The footprint of user devices is calculated by multiplying the number of phones sold in a given year by the lifecycle annual emissions, including embodied carbon spread over the expected lifetime and annual electricity consumption. One of the strengths of this study lies in the systematic review of useful life estimates in the literature for user devices which is more rigorous and provides a smaller range of estimates for user device embodied footprints than A&E more arbitrary useful life estimates. Data centres estimates are based on data from 2008 by Vereecken et al. [2009] and network estimates are based on 2008-2012 data from Van Heddeghem et al. [2014] which B&E projected to grow linearly.

B&E provide a breakdown of emissions for user devices, data centres and networks for each year 2007-2020, but model only the total carbon footprint of ICT from 2020 to 2040 by fitting a linear and an exponential growth curve to their estimates of total emissions from 2007 to 2020.

Findings

B&E estimate ICT's footprint in 2020 at between 1.11 $GtCO_2e$ (minimum) and 1.31 $GtCO_2e$ (maximum). The range is considerably smaller than in A&E and is due to uncertainties in the carbon footprint of user devices, mainly desktops and displays. Data centre and network estimates are the same for both minimum and maximum estimates. The authors suggest that including TVs could add another 435 TWh for operational energy use alone (assuming a global electricity carbon intensity of 0.6 $MtCO_2e$/TWh, this would add 261 $MtCO_2e$), assuming a 2% growth per year in number of TVs.

With regards to projections beyond 2020, both the linear and exponential curve show an increase (see Figure 4 in Section 2.1.2). The authors note that exponential growth, which would lead to between 2.48 and 2.62 $GtCO_2e$ in 2030 and 5.1 and 5.3 $GtCO_2e$ in 2040, is the most realistic, and that growth is highly likely if business as usual

continues. These predictions necessarily assume that trends active over the last decade continue for the next two decades and they assume unchecked growth. With an explosion of data traffic driven by trends like AI, Blockchain and IoT and the slowing down of efficiency improvements, there could be an additional jump in ICT's emissions within the next 3-5 years and an overall higher growth in emissions than modelled in their projections. However, while data centres will likely increase in power consumption, they might decrease in GHG emissions, if the trend of powering them with renewable energy continues [Belkhir in personal communication].

The predictions beyond 2020 are limited to totals and are not broken down by component but in their paper, the authors discuss the trend of wireless and mobile communications, cloud-based computing and IoT driving increases in data centres and networks. One of B&E main findings is the disproportionate impact of smartphones, whose footprint they estimate at 125 MtCO2e in 2020.[1] Most of this is due to their embodied footprint which is a concern in combination with their short average lifetime of 2 years. As many online data-heavy activities such as social media and video streaming are accessed by consumers on their mobile phones, the emissions associated with the data centres behind platforms like Facebook can be seen as a knock-on effect of mobile phone usage [Belkhir in personal communication].

Limitations and Criticism

B&E included the production and operational energy of ICT for user devices but they only considered the operational energy of data centres and networks, ignoring their embodied carbon because they found it to be negligibly small and excluding operator activities, potentially leading to a slight underestimate of total emissions.

B&E do not consider efficiency improvements in their estimates. For user device footprints this assumption might hold approximately as devices are 'upgraded' with more functionalities and a correspondingly higher footprint. However, for data centres, their estimates are based on extrapolation from 2008 emissions from data centres with a power usage effectiveness of 2, much higher than most modern data centres, without adjusting for efficiency improvements. This might explain why B&E's data centre estimate of 495 $MtCO_2e$ in 2020 is at the higher end relative to the other studies discussed here. Belkhir himself noted that their projection for data centres in 2020 is overestimated as efficiency improvements have unexpectedly been able to keep up with growing demand, even though this counterbalancing effect will soon come to an end as efficiency improvements slow down according to Belkhir.

While transparent about their sources, all peer-reviewed articles and publicly accessible industry reports, they did not make available supplementary data with the raw data for the total carbon footprint of ICT (with the exception of figures for 2020, 2030 and 2040) and user devices by year or their calculations. However, in personal communication with the lead author, we were able to get access to the raw data.

---

[1] This is based on 3.6 billion smartphones, including phablets, in 2020. Note that B&E did not include traditional mobile phones or fixed phones in the study's scope. B&E estimate 5.6 billion mobile phones to be in operation in 2030 and 8.7 billion in 2040, although data from Cisco [2020] suggests that the number of mobile phones could rise to 8.3 billion in 2023 already so there might actually be an even steeper rise in carbon emissions from smartphones.

B.2.5 Research by Malmodin and colleagues

Approach

Malmodin and Lundén [2018], hereafter M&L, use a hybrid top-down/bottom-up approach and draw on primary industry data from major manufacturers, sales statistics and LCAs for equipment. Emissions of user devices were modelled: 1) bottom-up based on the number of shipped and in-use devices in 2015 and the embodied and use phase emissions per unit estimated in LCAs, and 2) top-down the energy and carbon footprints of 35 major ICT and E&E manufacturers (reported in the supplementary materials) which were extrapolated based on those companies' share of revenue. Embodied footprints of user devices are the most uncertain part of their study.

Network footprints are based on a top-down analysis of network electricity consumption published by the authors [Malmodin and Lundén 2018b] which draw on anonymised operator data covering 70% of subscriptions, which was extrapolated to the global level. Data centre emissions are based on a mix of public and anonymised operator data. The authors note that it was not easy to get hold of primary data from networks and data centres operators because it is considered competitive. The operator data are therefore anonymised; however, they report data collected from public reports in the supplementary materials.

M&L only estimated ICT's global emissions for 2015 but in personal communication, Malmodin has shared his more recent and yet unpublished estimates for 2018 and 2020 with us (see Table B.**6** and Table B.**7**) which follow the same approach as M&L but are based on more recent operator data. We are also drawing on a presentation given by Malmodin at Energimyndigheten in 2019 [Malmodin 2019].

Table B.6 *Breakdown of ICT's carbon footprint as provided by Malmodin [personal communication].*

$MtCO_2e$	2015	2018	2020
ICT without TV	730	705	690
Networks	182	173	168
Data centres	141	129	127
Enterprise networks	17	16	15.5
User devices	392	380	375
TVs, TV networks and other consumer electronics	420	N/A	400
ICT with TV	1,153	N/A	1,087

Table B.7 *Operational electricity consumption by the ICT industry as provided by Malmodin [personal communication]. The total is adjusted for double counting.*

Operational TWh	2015	2018	2020
ICT Total	803	836	859

Networks	222	243	257
Data centres	220	225	230
Enterprise networks	25	25	25
User devices	343	349	353

Findings

Malmodin and colleagues argue that ICT's global emissions have broadly stabilised; they increased slightly from 720 $MtCO_2e$ in 2010 to only 733 $MtCO_2e$ in 2015 and decreased to 690 MtCO2e in 2020 (this includes user devices, networks and data centres). Relative to 2015, emissions from data centres have decreased the most (by 10%) followed by enterprise networks (9%) and networks (8%), even though electricity consumption increased in that time period due to the continued build out of 4G and 5G networks and an increasing number of data centre servers. The reason why the increased total energy use by ICT does not translate into higher carbon footprints is that it is partly offset by a higher share of renewable energy, a slight decrease in network overheads and that embodied carbon has stayed largely the same. The E&M sector (specifically, TVs, TV networks and other consumer electronics) add another 420 $MtCO_2e$ in 2015 [M&L] and 400 $MtCO_2e$ in 2020 (see Table B.**3**), showing a slight decrease too.

M&L further argue that ICT and E&M sector growth is starting to decouple from GHG emissions as ICT use is continuing to grow in terms of number of users and data traffic, albeit slower than previously as the world is moving towards saturation.[2] M&L argue that electricity consumption in data centre and networks does not follow the same exponential growth curve because of efficiency gains by servers. They show that the carbon footprint per subscription and per GB of data in networks has decreased fast since the 1990s and argue that while the number of networks users has increased by a factor of 10 and data traffic has increased 10,000 times between 1995 and 2015 if voice traffic is included, yet ICT's carbon footprint has only tripled during that time. They hold that ICT's footprint does increase with use but is better correlated with the number of users rather than data traffic. M&L acknowledge that Moore's Law has slowed down since 2012/13 but note that there usually a time lag until the effects are felt outside of research labs. In personal communication, Malmodin also noted that so far, efficiency improvements are continuing. The decoupling is also helped by enablement of emission savings in other industries, for example from print media as newspapers increasingly shift online, even though they admit that the effect still has to be seen in other sectors like transport.

These predictions stand in contrast to an earlier study [Malmodin et al. 2013] that projected ICT's footprint at 1.1 $GtCO_2e$ in 2020 plus an additional 1.3 $GtCO_2e$ for the E&M sector, including 680 $MtCO_2e$ for TVs and TV peripherals. This study argued that the increase in emissions would be driven mainly by an increase in the number of devices and therefore network subscriptions and data traffic, which will have been partly counterbalanced by energy efficiency improvements in networks, more efficient

[2] Malmodin [2019] argues that data traffic grew 70 times 1995-2000, 15 times 2000-05, 4 times 2010-15, 3 times 2015-20.

TVs, a shift from desktops to laptops and lower standby electricity consumption.

Explaining the discrepancy with earlier predictions, M&L argue that their earlier study was still based on older data which assumed the historical growth of PC and TV sales would continue, whereas more recent research takes into account the peak of PCs around 2011 and a slow decline of TV and tablet sales as well as better power management. The only PC type that is expected to increase is gaming PCs which have a higher carbon footprint. Consumer electronics sales (e.g. cameras, media players) are also declining thanks a move to smaller devices like laptops, tablets and smartphones and in particular the integration of functions into smartphones which *replace* older and less efficient user devices. There is also a shift from traditional storage devices (e.g. memory sticks) to cloud storage.

At the same time, they argue that M2M communication and IoT are only adding a very small footprint. In contrast to earlier studies by Malmodin [e.g. Malmodin et al. 2010, 2013], M&L's study included several IoT devices, including wearables, smart energy meters control units, surveillance cameras, public displays, payment terminals and the internet-connected communication device in vending machines. For 2015, they concluded that their impact on emissions was marginal. However, they did not include other now-common IoTs like smart speakers or any connected devices from other sectors, such as those embedded in vehicles, buildings or IoT used for military, medical, security and industrial purposes, other than those listed above, although they note that these are expected to add to GHG emissions in the future. The authors note that the number of IoT devices might explode and that M2M communication and therefore the number of network subscriptions are likely to increase rapidly in the future too. Already between 2010 and 2015, the number of M2M IoT subscriptions increased from 70 million in 2010 to 350 million in 2015.

A report by Ericsson [2019] presents a 2020 scenario which assumed a large increase in IoT and other new devices, with 500 billion sensors and tags, one billion connectivity boxes in ICT and 27 billion connectivity modules in other sectors, and found that their life cycle emissions were only minimal. The report argues that data traffic is driven more by video than by IoT. However, the methodology underlying this analysis is not provided.

Malmodin [2019] thinks that ICT can halve its emissions by 2030 relative to 2020 through renewable energy transformation and through collective effort. He expects data centres to be 1% of global electricity use even in the future, even though the absolute amount is expected to go up. In a recent Ericsson blogpost building on Malmodin's work, Lövehagen [2020] claims that ICT's carbon footprint could be reduced up to 80% if all electricity came from renewable energy. Importantly, she makes clear that ICT could be both a tool for decreasing or *increasing* global carbon emissions by accelerating carbon-intensive processes, depending on how it is used.

Another reason for stabilisation of emissions for Malmodin is his belief that there are limits to ICT's carbon footprint as smartphone markets saturate and there are a limited number of hours per day that users can use ICT equipment [personal communication]. Malmodin [personal communication] notes that the impact of AI and machine learning

is so far very small even though it has been around for a while, and that emissions from AI are unlikely to explode unless training AI becomes more efficient.

Limitations and criticism

M&L and Malmodin's follow-up research present the most recent research on global ICT emissions reviewed here and the one with the widest scope in terms of ICT equipment, lifecycle stages and supply chain emissions considered.[3] Unlike Belkhir's and Andrae's studies, the estimates are based on more recent and a wider range of measured data directly from industry rather than older data reported in the academic literature. This is valuable as trends in ICT can change fast.

However, this data is also the biggest weakness, as it is not made public and cannot be scrutinised by the reader. Apart from one LCA for smartphones [Ercan et al. 2016], LCAs for device embodied and use phase carbon are not based on peer-reviewed LCA studies but on data by market research companies like IDC, IHS and Gartner whose reports are not available for free, even through university subscriptions. They are therefore not easily accessible for the reader either.

In personal communication, Malmodin noted that data centre emissions might be overestimated by up to 13 $MtCO_2e$ for 2020 if the use of green electricity is taken into account, even though the investigation is still ongoing. For 2015, he notes that M&L should have used 0.63 $kgCO_2e/kWh$ instead of 0.6 $kgCO_2e/kWh$. This would change the use phase carbon footprint upward by 5%.

Malmodin has been challenged about his opinion that ICT's carbon footprint has reached a peak and that energy growth is slowing despite data traffic increases, as he believes energy use is largely unrelated to data traffic, and his assumption that energy efficiency gains will continue [Belkhir and Andrae in personal communication]. This debate is examined in more detail in Sections 2.2.1 and 2.2.4.

B.3 Drivers of change in ICT Future Emissions

A&E predict that over the next decade, user devices will become less important and networks and data centres will become more important for ICT total emissions. This is partly due to: a shift from desktops and laptops to phones; an increase in data traffic through trends like video streaming, cloud computing and emerging new data-heavy technologies; and a higher share of mobile data transmission because of the popularity

[3] Unlike A&E and B&E, M&L consider all stages of ICT equipment lifecycle, that is material acquisition, parts and component production and assembly, transport and end of life. In addition to 'classic' ICT, they include the entertainment and media (E&M) sector, which includes TVs and other consumer electronics, such as cameras and audio systems in a car and portable GPS, e.g. for use in cars (see Table B.*2*). The study also includes operator activities and overheads, such as offices and business travel used by data centre and network operators, and enterprise networks, which are wireless and wired networks within business buildings that are operated by the company. The network emissions include the embodied carbon of infrastructure, like digging cable ducts and constructing antenna towers spread over its lifetime. ICT used by the financial system is also in scope, including computers, TVs, networks and servers, but not cryptocurrencies with the rationale that mining cryptocurrencies required *specific* hardware, not regular servers. This rationale has been challenged by Belkhir [personal communication] as unreasonable as mining computers and servers use GPUs, which are found in gaming and are therefore within the scope of ICT. AI is included indirectly by covering all data centre emissions with a top-down approach.

of smartphones. In more recent papers [Andrae 2019a, 2019b, 2019c], Andrae also argues that trends such as AI and deep learning, IoT, Blockchain, virtual and augmented reality, facial recognition, and the rollout of 5G could lead to an explosion of data traffic over the next decade—increasing the share of data centres in ICT's emissions. While the trend towards smaller devices like smartphones is helping reduce ICT's emissions, we are also adding more devices like smart speakers and IoT.

B&E point to the rising footprint of smartphones, with a share of 11% of total ICT emissions in 2020. As the number of users and the amount of data-heavy activities like video-streaming and social media on smartphones increases, this in turn contributes to an increase in mobile network and data centre use. Due to their short lifetime and increasing energy efficiency, the vast majority of smartphones' emissions are embodied. They predict that data centres' electricity use is going to rise but that emissions might stabilise if the trend of powering them with renewable energy continues. Network emissions are increasing slowly, and PCs' emissions are decreasing.

In contrast, M&L believe that AI and Machine Learning (ML) will not play a large role, unless training AI becomes more energy efficient because it would not be economical to run. Their assessment of IoT (even though with limited scope – see Appendix B.2.5) led them to conclude that the impact of IoT is, and will continue to be, minimal in the foreseeable future due to low data volumes; this is despite a possible explosion in the number of devices and network subscriptions. TV and PC emissions are decreasing due to better power management, lower standby power consumption and decreased sales. Their view is that other electronics are also declining – helped by a shift to smartphones which integrate functions such as video streaming, cameras, and portable media players, into one device. They highlight the large energy consumption of user access equipment that is on 24/7, such as modems, routers and set top boxes. They argue that the growth in data traffic is slowing down and that data centres and networks electricity consumption will not grow exponentially alongside the growth of data traffic because of efficiency gains and shifts to renewable energy.

B.4 Reports out of scope of the review

There have been several reports in recent years on the topic of ICT's emissions, including on behalf of the Global eSustainability Initiative (GeSI), which represents ICT companies. In their report SMART 2020, produced by The Climate Group, they estimated ICT's emissions at 530 MtCO_2e in 2002 and 830 MtCO_2e in 2007 and projected ICT's footprint to rise to 1,430 MtCO_2e in 2020 under business as usual. In a later report compiled by BCG, SMARTer 2020 [GeSI 2012], they estimated emissions in 2011 at 0.91 GtCO_2e and revised their 2020 projection to 1.27 GtCO_2e. Their latest report, SMARTer 2030 [GeSI 2015], compiled by Accenture, extends their earlier projections to 2030 with an estimate of 1.25 GtCO_2e. The scope is summarised in Table B.**1**; for the 2015 report, it also includes 3D printers. In their reports, they also discuss 'abatement potential' by ICT in other industries whereby ICT could save 9.1 Gt CO_2e in 2020 and 12.08 Gt CO_2e in 2030; we explore these trends in more detail in Section 2.2.5. Another report claiming emission reductions of 2.1 GtCO2e enabled by mobile technology was released by GSMA [2019], which represents mobile operators.

Policy Connect, a London-based thinktank, produced a report *Is Staying Online Costing The Earth?* [McMahon 2018] sponsored by Sony in 2018 that concluded that energy consumption by ICT is not necessarily going to rise due to efficiency gains, renewable energy, a trend to smaller devices and ICT-enabled carbon savings in other industries.

The report *Lean ICT – Towards Digital Sobriety,* produced by The Shift Project [2019b], a Paris-based thinktank, came to a very different conclusion. They projected that ICT emits between 2.1 and 2.3 $GtCO_2e$ in 2020 and between 3.3 and 4.2 $GtCO_2e$ in 2025, including embodied and use phase carbon for PCs, phones, tablets, TVs, some IoT, networks and data centres. The modelling is based on Andrae and Edler's (2015) study but with updated assumptions, such as data traffic and the number of devices used. These estimates lie between Andrae and Edler's expected and worst case and therefore much higher than what the three main experts whose papers we reviewed above believe. The report points to several important trends, such as the impact of video streaming and short-lifespan devices, the underestimation of ICT's emissions by consumers because the underling infrastructure is invisible, and the unequal distribution of data consumption with high-income countries benefitting and thus emitting more than low-income countries.

These reports have not been included in the detailed review as they are not peer-reviewed. In addition, there are potential conflicts of interest where reports are sponsored by ICT companies [e.g. McMahon 2018; GeSI 2015; GSMA 2019]. Policy Connect's report largely relies on M&L's study, which is included in our detailed review, rather than offering original insights. For The Shift Project's [2019b] report, new modelling was done based on A&E's study but the findings have been discredited by all of the experts consulted (including Andrae, Belkhir, Malmodin and Preist). In the case of GeSI, the modelling behind the report is not transparent and assumptions are not made clear so it cannot be fully assessed.

C Video Streaming

Video streaming has become the dominant driver of data traffic consumption - forming 60% of downstream traffic and 22% of upstream traffic globally in 2018 [Sandvine 2019]. This traffic demand has been driven by adoption of video-on-demand services offered by companies such as Netflix, Amazon Prime and Disney; the popularity of YouTube and the embedding of video clips into other online services (e.g. social media such as Facebook and Twitter); and the use of video for security surveillance and video conferencing.

If travel is fully replaced by video conferencing, video offers significant carbon savings.

Online video can most prominently provide opportunities for reduction in travel-related carbon emissions. For example, video conferencing for co-locating a conference can create significant emission reductions from flights [Coroama et al. 2013], creating dematerialisation if the potential of this media is *“actively sought and unleashed”* [Coroama et al. 2015]. Video streaming has shown how useful it is during the Covid-19 outbreak, allowing entertainment during isolation as well as supporting home working. During the pandemic: replacing physical face-to-face meetings, for example, will reduce the travel-based emissions from business flights and peoples' commutes to work; we have also seen academic conferences moving online. However, as highlighted in Section 2.2.5, the rise in video traffic and availability of video

conferencing has not yet led to a reduction in air travel [Graver et al. 2019], although this may change following the Covid-19 crisis.

Video is accelerating data traffic.

Video is clearly a prominent driver in data traffic which could significantly add to ICT's growth and emissions (Section 2.2.4). For example, higher streaming qualities such as High Definition (HD) and Ultra HD (UHD) can have a *"multiplier effect on traffic"*: 4K (UHD) doubles the bit rate of HD video and multiplies the bit rate of Standard Definition (SD) by nine [Cisco 2020]. Streaming qualities also affect device adoption, e.g. 66% of flat-panel TV sets are expected be UHD in 2023 (doubling the 33% share in 2018) [Cisco 2020], therefore impacting the embodied emissions of video-focused devices as users replace older TVs with newer models. In addition, faster infrastructure (e.g. 5G, fibre to the home) enables applications such as UHD cameras and VR streaming [Cisco 2020], multiple simultaneous streams within households [Widdicks et al. 2019], and now data-intensive gaming activities [Vaughan 2019] – driving the demand for video related network traffic and high performance streaming infrastructure such as content delivery networks and data centres further.

Changes are required to stop continuous video and internet infrastructure growth.

The Shift Project [2019a] estimated that 300 $MtCO_2e$ was generated in 2018 due to online video and argue that this is comparable to annual emissions of Spain in 2010. These estimates have come under scrutiny [Kamiya 2020] due to arguments that they were based on old data, that energy impacts of the internet are much lower [cf. Shehabi et al. 2014] and that energy intensities of data transmissions are halving every two years [cf. Aslan et al. 2017] – following the 'Efficiency saves ICT' narrative (Section 2.2.2). These arguments also underpin some criticisms by TechUK [Fryer 2020] on a recent documentary BBC iPlayer [2020] "Dirty Streaming", arguing the documentary provides misleading or incorrect information on ICT's environmental impacts.

The Shift Project may overestimate absolute emissions due to the direct processing of video traffic – especially as the energy per bit does improve over time, there is evidence that data traffic, including video, links more potently to growth in infrastructure and capacity. Preist et al. [2016] argue that growth in the internet's infrastructure capacity allows for new data-intensive services and applications (of which video is a part) – offering new affordances to users, in turn driving demand for these services and therefore further infrastructure growth. Peak data traffic is one driver for this infrastructure growth due to increased demand for data-intensive services; other influences include overprovisioning the infrastructure to ensure these services are always available to all users even at peak times [Preist, personal communication]. Growth begets more growth, unless we put a ceiling on absolute demand. In addition, Belkhir [personal communication] highlighted that the agreement between Netflix (a major video streaming service) and EU regulators to ease Netflix's load on the network during the Covid-19 pandemic [Sweney 2020] makes it difficult to argue data traffic is not interlinked with ICT infrastructure growth.

This is where changes in online service design may have a positive impact, e.g. turning off the video for a large portion of YouTube users who are only *listening* to the content [Lord et al. 2015, Widdicks et al. 2019] can have comparable emission reductions to running data centres on renewable energy [Preist et al. 2019] — but much more will need to be done to mitigate the significant growth of video streaming.

D Narratives

The assumptions about efficiency improvements and demand for ICT and predictions about ICT's impact on emissions in the scientific literature and non-scientific reports and the media can be summarised in the form of six common 'narratives', as detailed below. Note that Rebounds in ICT, Rebounds stalled and Global Rebounds are theoretical possibilities for which there is some evidence in the scientific literature (Appendix I); however, they are not commonly discussed in the literature on ICT's emissions.

The first four narratives relate to ICT's own emissions, and the final two relate to ICT's impact on the rest of the global economy. The arguments underlying each narrative are underlined; we explore these in Section 2.2.

Efficiency saves ICT

Efficiency improvements are continuing; in combination with a shift towards more renewable energy, this will offset increases in ICT's energy use, stabilising ICT's emissions at the current level or even decreasing it in the future. Emissions are not so much influenced by the increasing data traffic but rather by the number of users, which will naturally level off soon as the world reaches saturation for personal ICT devices.

E.g. research by Malmodin and colleagues, Masanet and colleagues

Growth without efficiency

The growth in data traffic will lead to increases in network and data centre energy use, while the growth in IoT will lead to increases in embodied device emissions.

A) In combination with efficiency improvements slowing down, this will lead to an exponential growth in ICT's emissions.

B) Even if efficiency improvements continue, they will lead to further emission growth because of Jevon's paradox (Appendix I), unless emissions are capped.

E.g. research by Andrae and colleagues, Belkhir and colleagues

Rebounds in ICT

The efficiency improvements enabled by ICT in other sectors lead to system growth within ICT. Under current conditions, rebound effects are greater than 100%. Therefore, the net effect of efficiency through ICT's is a rise in global emissions. If efficiencies continue, ICT's emissions will also increase unless they are deliberately constrained.

Rebounds stalled

If efficiency improvements stall (for example because Moore's Law reaches its quantum limit), this will lead to a plateau of emissions because growth requires efficiency gains.

Enablement

Because ICT enables carbon savings in other industries, the net effect of ICT is to lower global emissions despite growth in the ICT sector's own footprint.

E.g. GeSI's SMARTer 2030, GSMA's The Enablement Factor report

Global Rebounds

ICT enables efficiencies in other sectors which lead to growth in the wider economy. Rebound effects are larger than the efficiency gains (i.e. greater than 100%) and lead to an overall increase in global emissions.

E Truncation Error

There are two core methodologies for estimating the embodied carbon: the more commonly used Life Cycle Analysis (LCA) and Environmentally Extended Input Output (EEIO) analysis. LCA has potential for greater specificity as it is tailored to specific models, such as an iPhone 11, but inevitably incurs a truncation error; an underestimation arising from LCAs being unable to include the infinite number of supply chain pathways. To illustrate, a factory manufacturing computers will itself use computers to manage the production, a small share of whose embodied carbon needs to be attributed to the factory's output. Most of the literature assessing the embodied carbon in ICT is LCA-based.

EEIO offers a much more generic estimate, based on macro-economic modelling of financial and carbon flows between industrial sectors. It provides estimates of the total carbon emissions resulting from production of different types of goods per unit of monetary value. Whilst lacking specificity (i.e. all goods within broad categories, such as 'manufacture of office machinery and computers', have the same carbon footprint per dollar), EEIO-based estimates have the important advantage of taking account of emissions from all supply chain pathways; they do not incur truncation error.

To get some of the best of both approaches, it is possible to combine LCA and EEIO methodologies by approximating and adjusting for the truncation error incurred by LCAs. This can be done by mapping the LCA's system boundaries onto the EEIO model. Such a hybrid methodological approach stands to have both the specificity of LCA and the system-completeness of EEIO. In this report, we have drawn upon work carried out by SWC (see Appendix A.5.4) to derive adjustment factors for LCAs in different product categories and applied these to LCA-based embodied carbon assessments to derive system-complete estimates.

Based on SWC's EEIO model, we estimate that truncation error causes an omission of ca. 40% of the total embodied carbon and ca. 18% of the use phase carbon. When this is factored in, adjusting each study's specific electricity intensity figures, estimates for 2020 are on average 25% higher. Table E.**1** shows A&E, M&L and B&E LCA carbon estimates without adjustment of truncation error; Table E.*2* shows the adjusted estimates when truncation error is taken into account. This is just an approximation. We reiterate the caveat that SWC's EEIO model is based on UK data which is not representative of the world economy yet we have applied it to A&E's, M&L's and B&E's global estimates for a rough estimate of underestimation. We also note that these studies likely incur truncation errors of different sizes due to their differences in methodology. We have only adjusted for these differences with respect to electricity carbon intensity, but not for embodied emissions. Due to its more inclusive scope, M&L's is likely to have a smaller truncation error and A&E likely has a larger one than the average truncation error assumed here.

Table E.1 Original estimates of embodied and use phase carbon for 2020. Malmodin's and A&E's estimates include TVs, B&E's estimates do not.

Study	Embodied ($MtCO_2e$)	Use phase ($MtCO_2e$)	Total ($MtCO_2e$)	$MtCO_2e/TWh$
Malmodin (2020)	300	787	1087	0.60
B&E - 2020 minimum	213	894	1107	0.50
B&E - 2020 maximum	349	957	1306	0.50
B&E - 2020 average (calculated)	281	926	1207	0.50
A&E - 2020 Best case	157	730	887	0.59
A&E - 2020 Expected case	326	1534	1860	0.59
A&E - 2020 Worst case	1024	2610	3634	0.61

***Table E.2** Estimates for 2020 adjusted to include all supply chain pathways.*
**Included in the average. A&E's best case was chosen because Andrae (2020) reported that this is the most realistic for 2020. An average calculated for B&E's minimum and maximum estimates was included sinde B&E did not endorse either scenario and we wanted to avoid skewing the average by considering two of their estimates.*

	Embodied ($MtCO_2e$)	Use phase ($MtCO_2e$)	Total ($MtCO_2e$)	$MtCO_2e/TWh$
Malmodin (2020)*	500	826	1326	0.63
B&E - 2020 minimum	355	1127	1482	0.63
B&E - 2020 maximum	582	1206	1788	0.63
B&E - 2020 average (calculated)*	469	1166	1635	0.63
A&E - 2020 Best case*	262	781	1043	0.63
A&E - 2020 Expected case	543	1628	2171	0.63
A&E - 2020 Worst case	1706	2704	4410	0.63
**Average**	**410**	**925**	**1335**	**0.63**

For ICT, TV and other consumer electronics, the adjusted total ranges between 1.2 and 2.2 $GtCO_2$ in 2020 (2.1-3.9% of global GHG emissions, see Table E.**3**) with 30% coming from embodied carbon and 70% from use phase on average.

Table E.3 Estimates for 2020 adjusted to include all supply chain pathways and brought to the same scope. *Included in the average.

	**Embodied ($MtCO_2e$)**	**Use Phase ($MtCO_2e$)**	**Total ($MtCO_2e$)**
Malmodin (2020)*	500	826	1326
B&E's Minimum + Malmodin's E&M figure	457	1482	1940
B&E's Maximum + Malmodin's E&M figure	684	1562	2246
B&E's Average (calculated) + Malmodin's E&M figure*	571	1522	2093
A&E's Best without TV + Malmodin's E&M figure*	296	898	1194
**Average**	**456**	**1082**	**1538**

This scope does not include some ICT equipment, such as radios, Blockchain and most IoT. Using EEIO, Livia Cabernard from ETH Zurich [Cabernard 2019, Cabernard et al. 2019] estimates that ICT's embodied carbon footprint (including manufacturing and transporting and covering a wider scope of ICT, specifically computers, mobile phones, TVs, radios, office machinery and all embedded ICT) was ca. 1.1 $GtCO_2e$ in 2015. Of the 1.1 $GtCO_2e$ embodied emissions, 27% were from computers, 55% from radio, TV and mobile and 18% from ICT embedded in other end products. Over the last few decades, a production shift to China (61% of ICT production in 2015, 45% in 1995) has increased the embodied carbon footprint of ICT because China's electricity use is mainly from coal [Cabernard in personal communication].

Modelling future population growth, efficiency improvements in relevant sectors, such as steel production, and a transition from coal to renewable energy in line with actions taken to limit global warming to 2°C (6°C), Cabernard [personal communication, based on Wiebe et al. 2018] predicts that ICT's embodied carbon footprint could be 1.38 (1.5) $GtCO_2e$ in 2020, 1.27 (1.56) $GtCO_2e$ in 2025 and 1.16 (1.64) $GtCO_2e$ in 2030 for embodied emissions of ICT manufacturing. That means that the estimates for embodied carbon in Table E.**3** only cover a third of ICT's true embodied carbon footprint, because some ICT equipment, such as radio, Blockchain and embedded ICT, is left out of scope.

In summary, in most research on ICT's emissions, ICT's embodied carbon is considerably underestimated. While users are keeping their PCs and smartphones for slightly longer, the manufacturing footprint of smartphones is increasing because of more advanced integrated circuits, displays and cameras [Malmodin in personal communication]. With the number of IoT devices predicted to grow exponentially, the embodied footprint of ICT is likely to increase in the future.

F European Commission's Investment in ICT

F.1 Artificial Intelligence

The European Commission has ambitions to significantly increase uptake of AI as a way of *"strengthen*[ing] *the competitiveness of European industry"* [European Commission 2019c]. Specific initiatives and funding streams include Digital Innovation Hubs [European Commission 2018b] to *"provide support to SMEs to understand and adopt AI"* [NoCash 2020], and InvestEU (more on this below). The EC recently funded the 50 million Euro project "ELISE" which aims to *"make Europe competitive by setting up a 'Powerhouse of AI'"* [FCAI 2020]. New Common European data spaces are also being set up to enable greater data sharing [Kayali et al. 2020], which may be used by AIs. The Commission also views AI as a *"driving force to achieve the Sustainable Development Goals"* [European Commission 2019c], so is funding *"competitions and missions for AI solutions tackling specific environmental problems"* [European Commission 2019d]. Examples of how AI is expected to produce emissions reductions in other sectors include *"increasing the efficiency of farming, contributing to climate mitigation and adaptation,* [and] *improving the efficiency of production systems through predictive maintenance"* [European Commission 2019c].

F.2 Internet of Things

The European Commission has launched the Alliance for Internet of Things Innovation to *"to support the creation of an innovative industry driven European Internet of Things ecosystem"* [European Commission 2019a]. Within the Digitising European Industry initiative, the Commission identifies three foci: 1) *"a thriving IoT ecosystem"*; 2) *"a human-centred IoT approach"*; and 3) *"a single market for IoT"*. The latter is facilitated through the *"European data economy"* initiative, which *"proposes policy and legal solutions concerning the free flow of data across national borders in the EU, and liability issues"* [European Commission 2019a]. The IoT is seen as playing a key role in the European response to climate crisis through providing an infrastructure to enable the 'smart future' (see, e.g. [ETIP SNET]), and for distributing energy consumption across smaller data centres [Gilmore 2018].

F.3 Blockchain

The European Commission has established the European Blockchain Observatory and Forum to *"accelerate Blockchain innovation and the development of the Blockchain ecosystem within EU and* [to] *help cement Europe's position as a global leader in this transformative new technology"* [EU Blockchain 2020]. Along with AI, Blockchain is the target of the 2 billion Euro joint European Commission and European Investment Fund InvestEU Programme. In addition, the European Blockchain Partnership [European Commission 2018a] commits all member states to *"realising the potential of Blockchain-based services for the benefit of citizens, society and economy"* [European Commission 2020]. As one of its potential benefits, Blockchain is viewed by the Commission as the kind of 'disruptive technology' required by the climate crisis [European Commission 2019b], and the Commission has outlined five core areas they seek to 'unleash' Blockchain technology in service of climate. Based on the idea that Blockchain can *"improve the transparency, accountability and traceability"* of GHGs, these application areas include areas of clean power, smart transport systems, sustainable production and consumption, sustainable land use, and smart cities and homes.

G Carbon Pledges

Company pledges vary in particular with regards to scope of emissions covered (see Table G.1), offsets used and how much emissions are reduced. Note that there are also some companies with plans to cut emissions by a certain percentage but without the sweeping ambitions of the below pledges.

Table G.1 Emissions covered by Scope 1, 2 and 3.

Scope 1 emissions	Direct emissions from burning of fossil fuels on site (includes company facilities and vehicles).
Scope 2 emissions	Indirect emissions from purchased electricity and gas.
Scope 3 emissions	All other indirect emissions in a company's value chain including upstream emissions in the supply chain (e.g. emissions from purchased goods and services, transportation of these goods to the company, use of leased assets such as offices or data centres, business travel and employee commuting) and downstream emissions (from transportation, distribution, use and end of life treatment of sold products, investments and leased assets). Scope 3 emissions form the majority of a company's emissions.

G.1 Carbon Neutral

Carbon neutral means no net release of carbon-dioxide emissions into the atmosphere, through using offsets to counterbalance any emissions generated.[4] For a company to be carbon neutral, they must measure, reduce, and offset their emissions.

There is no set standard on which (if any) scope 3 emissions should be included, how these emissions should be reduced (purchasing credits or on-site renewables, for example), and which offsets are robust and credible.

Companies pledging carbon neutral: Sky (since 2006), Google (since 2007), Microsoft (since 2012), and Apple (by 2030).

G.2 Net Zero

Net zero is defined by the Science Based Targets initiative (SBTi) as a company having no net climate change impact through GHG emissions in a company's value chain. This is achieved by reducing GHG emissions in the value chain and removing the remaining emissions through additional carbon removals. Companies setting a net zero pledge can register with the SBTi if their targets Net zero means no net climate change impact through GHG emissions in a company's value chain, achieved by reducing GHG emissions in the value chain and removing the remaining emissions through additional carbon removals. A net zero target is more ambitious than a carbon neutral target because it specifies that scope 3 emissions need to be included, while for carbon neutral, generally only emissions from business travel and waste are included but not other scope 3 emissions.

[4] Note that most ICT companies refer to carbon dioxide and other GHG when they use the term 'carbon neutral'. This is sometimes called 'climate neutral'.

Organisations can register their net zero targest with the Science Based Target initiative (SBTi) which certifies organisations whose emission targets are in line with the IPCC's recommendations that global warming be limited to 2°C, well below 2°C or more recently to 1.5°C [IPCC 2018, Pineda and Faria 2019]. According to the SBTi, for company pledges validated after July 8th 2020, only two thirds of scope 3 emissions need to be accounted for and scope 3 emissions targets 'generally need not be science-based'. A target needs to be set for reducing scope 3 emissions only if they make up over 40% of a company's total emissions [SBTi 2019]. However, the Carbon Trust believes that net zero should apply to 100% of scope 3 emissions [Stephens 2019].

Net zero is also more ambitious than carbon neutral regarding offsets. SBTi specifies that 'offsets must not be counted as emissions reduction toward the progress of companies' science-based targets' [SBTi 2020]. In the way Sky and Microsoft define net zero, any emissions that cannot be reduced need to be removed from the atmosphere rather than being avoided, whereas carbon neutral can use both emission removal offsets and avoided emission offsets [Sky Zero 2020, Smith 2020].

Companies pledging net zero: Microsoft (by 2030), Sky (by 2030), Amazon (by 2040), BT (by 2045) and Sony (by 2050).

G.3 Carbon Negative

Carbon negative (also sometimes described as 'carbon positive' or 'climate positive') is where an activity removes more emissions than it emits across an entire value chain. This goes further than both carbon neutral and net zero. There is currently no official definition or standards for this apart from the ones used by Microsoft for their carbon negative pledge [Smith 2020].

Companies pledging carbon negative: Microsoft (by 2050).

G.4 100% Renewable

100% renewable means that all of a company's power consumptions comes from renewable sources, such as solar, wind and hydro. It does not specify whether and how much the company's emissions should be cut. Unfortunately, often companies do not provide detail on whether they will generate additional renewable energy on-site, or buy renewable energy certificates, including unbundled REGOs which should not be seen as truly 100% renewable, in the authors' opinion (see Appendix I).

Companies pledging 100% renewable: Netflix (since 2018), Google (2017), Facebook (by 2020), Samsung (by 2020), Microsoft (by 2025), Sky (by 2020), Vodafone (by 2025), Apple (since 2018).

Please note: the achievement of these pledges is self-reported and not externally validated.

G.5 Why scope matters

Carbon neutral pledges are not enough for the world to limit global warming to 1.5°C because they do not require the company to account for scope 3 emissions, which form the majority of ICT's carbon footprint. Voluntary emissions reductions of company

Scope 1 and 2 emissions alone could theoretically be sufficient if every company in the economy played its part. However, without any kind of enforcement or reputational consequences, companies have a competitive advantage if they do not set of meet targets. In addition, companies could lower their scope 1 and 2 emissions by outsourcing carbon-heavy activities to suppliers, which would not decrease overall emissions. By signing up for scope 3 targets, companies take responsibility for their supply chain. They then have an incentive to encourage their suppliers to cut their emissions too, creating a snowballing effect in the economy.

H Renewable Energy Purchases

H.1 On-site Generation

On-site generation of renewable energy is the best option for purchasing renewable energy. This is because, with on-site generation, the company carries the set-up costs of the renewable energy project and there are fewer transmission and distribution losses than when electricity is sourced from the grid. It also ensures that additional renewable energy is created which is needed for a successful global transition away from fossil fuels.

H.2 PPA with Bundled REGOs

Companies can also buy a Power Purchasing Agreement (PPA) for renewable energy. PPAs do not cover the set-up costs of a renewable energy project (unlike on-site generation) but they pay for the cost of the power generation and receive a bundled Renewable Energy Guarantees of Origin (REGOs) that certifies that each 1 MWh of electricity purchased comes from a renewable energy project. REGOs ensure for each unit of renewable energy generated, only one company can claim the environmental benefits, avoiding double counting. PPAs ensure additionality because a company purchasing a PPA pays for additional renewable energy to be generated and fed into the electricity grid. They can also encourage the setup of new renewable energy projects because projects can attract funding from investors more easily with guaranteed buyers of PPAs lined up.

H.3 Unbundled REGO

Companies also have the option of buying an unbundled REGO without the actual energy to lower their scope 2 emissions. In this case, the environmental benefit of renewable energy gets separated from the energy itself. The company generating renewable energy can sell off the environmental benefit represented by the REGO but without selling the actual energy. The company buying the unbundled REGO can claim lower scope 2 emissions. Unfortunately, unbundled REGOs do not encourage greater power generation from renewable sources because the demand for REGOs is currently vastly outstripped by the supply thus making them very cheap. This means they are an ineffective instrument for investment into renewable projects [Scott 2019] and cannot claim additionality. Because unbundled REGOs cannot claim additionality and the company buying it lays a sole claim to the “greenness”, thereby not sharing it with other electricity users, unbundled REGOs raise the carbon footprint of the electricity grid [Hewlett 2017]. The separation also makes it harder to

track what renewable energy projects lie behind each certificate. A company wishing to reduce their Scope 2 emissions and become powered by 100% renewables should therefore look towards investing directly into renewable projects and PPAs in which bundled REGOs are purchased, crucially, with the underlying power.

I Jevons Paradox

In 1865, William Stanley Jevons predicted that as the UK's use of coal became more efficient, it would make coal more attractive and thereby would increase demand for coal rather than reduce it [Jevons 1865]. *Jevons Paradox* refers to a situation in which an efficiency improvement leads to an even greater proportionate increase in total demand, with the result that resource requirement goes up rather than down, as is often assumed. There is evidence that Jevons Paradox applies beyond coal [e.g. Alcott 2005, Sorrell 2009, Schaffartzik et al. 2014]. An example is the increased energy efficiency of new forms of lighting (such as electric lighting compared to gas lighting) which allowed lighting to be used more widely – increasing the total energy consumption from lighting. Another demonstration is the fact that electric trains are vastly more efficient than steam trains, let alone horses, yet the carbon footprint of land transport has continuously risen over the time period that these technological advances took place due to expanded use [Berners-Lee and Clark 2013].

While Jevons Paradox is linked with efficiency as the principal driver of rebound effects, the paradox is frequently linked more broadly to a wide range of socio-economic drivers leading to a perverse increase rather than decrease in input demand. Macro-economic models suggest that this 'backfiring' or *rebound effect* leads to savings being cancelled out completely on average and even *adding input* demand relative to previous levels through a variety of mechanisms.[5] At the global level, efficiency improvements in almost every aspect of life have gone hand in hand with rising energy demand and rising emissions.

It is sometimes argued that without the efficiency improvements, demand would have increased even further; this assumes that demand would rise independently of efficiency. It is also argued by some [e.g. GeSI 2015; UK Energy Research Council 2007] that rebound effects are less than 100% of the efficiency savings, but this often results from an incomplete consideration of rebound pathways, especially macro-economic effects. To assess the full impact of rebound effects, all parts of the economy and a longer timescale need to be considered. The only way to feasibly do this is to analyse the combined effect of all global efficiency gains in all sectors and to track this against global energy use. This analysis yields a total energy rebound averaging

[5] Jevons Paradox and rebound effects are explored in more detail in Berners-Lee and Clark's book *The Burning Question* (2013). Briefly, they argue that when we improve energy efficiency, the available energy becomes more productive and therefore more valuable, leading to increased use. This is because any energy saved bounces back as additional energy elsewhere, either because: 1) efficiency makes the use of the resource cheaper (e.g. lighting, cloud storage of more data than with traditional file storage), 2) the savings are spent on other activities with a carbon footprint, 3) lower resource use leads to lower prices which increases demand for the resource elsewhere, or 4) knock-on effects in other areas of the economy (e.g. when video conferencing enables forming relationships with people on the other side of the world, leading to more air travel to visit them). Resource use can also be displaced into another country (e.g. when burning of fossil fuel domestically is restricted to lower the country's emissions but fossil fuel is continued to be extracted for exports to other countries with fewer environmental concerns).

102.4% over the past 50 years (i.e. the annual global growth in energy use) [Berners-Lee and Clark 2013]. Despite the increasing utility per unit of energy, the world's energy use is increasing. The same holds true for emissions. Over the last 170 years, CO_2 emissions have been rising at 1.8% per year (with only temporary deviations on either side of that trajectory) [Berners-Lee and Clark 2013] alongside the growth of ICT, vast efficiency gains in ICT and other technological advances in other industries.

In terms of the ICT industry, it has been argued that it is through its increasing efficiency that computational power has risen and ICT has been able to become so important in society; the energy consumption of early computers would have been prohibitive for the scale of expansion we have seen over the last decades [Aebischer and Hilty 2015]. An analysis of dematerialisation by Magee and Devezas [2017] found evidence that, in the ICT industry, efficiencies in the material needed for a single product lead to either increasing performance or reduced prices and that this inadvertently leads to increases in demand, resulting in an increase in absolute material consumption. Silicon is one example as it holds a special place in information storage, transmission and computing. Other examples of rebound effects in ICT are provided by Gossart [2015], Galvin [2015] and Walnum and Andrae [2016]. Galvin [2015] estimates that rebound effects in ICT's energy use could range between 115% and 161% based on eight case studies, as efficiency is more than offset by increases in demand.

In addition to efficiencies within the ICT industry, ICT-delivered efficiencies can also have far-reaching effects in other industries – in what we will call *Global Rebounds*. In recent years, ICT has increasingly expanded into other sectors. Common examples include video conferencing technologies or online shopping which could reduce the need to physical travel or reading news on a smartphone. These have the potential to both decrease and increase environmental impact. Where these new technologies evolve to be more energy intensive than their alternatives (e.g. high-quality video streaming), where they are used *in addition* rather than as a substitute (e.g. e-books being used alongside paper books), or where they allow intensified activity or growth in other industries because they are cheaper, more productive or more convenient (e.g. more regular checking of news on a smartphone than with traditional newspapers leading to increased need for news production), the impact of the economy as a whole in terms of energy use, resource use or GHG emissions can increase [Court and Sorrell 2020].

In a systemic review of the direct and economy-wide impact of e-materialisation (such as e-publications, e-games, e-music etc.) on energy consumption, Court and Sorrell [2020] found that studies systematically neglect rebound effects. Most studies assume substitution of old technology with the new digital system where this assumption is not always justified, leading to overestimates of energy savings. Assumptions around the lifetime, the number of users, efficiency of user devices and the replacement of travel lead to a wide range of predictions from 90% decreases to 2000% increases in energy consumption. They conclude that there is no conclusive evidence suggesting significant current or future energy savings from e-materialisation. There is another aspect to efficiency: psychological spillovers through moral licensing where people feel that they have done their part for the environment when increasing efficiency and then go on to have an increased environmental impact elsewhere [Sorrell et al. 2020] - but this is out of scope for this report.

The net effect of ICT depends on the balance of impacts it has both through its own emissions and the effects it has on the wider economy. The economy-wide effects of ICT are difficult to quantify, but in the absence of solid evidence, it would at the very least be risky to assume that the Jevons Paradox and other rebound effects (e.g. time rebounds [Börjesson Rivera et al. 2014]) do not apply to ICT's direct and economy-wide impact.

References

1. Aebischer, B. and Hilty, L.M., 2015. *The energy demand of ICT: a historical perspective and current methodological challenges*, in *ICT Innovations for Sustainability*. Springer. p. 71-103.
2. Alcott, B., 2005. *Jevons' paradox.* Ecological economics, **54**(1): p. 9-21.
3. Andrae, A.S., 2019a. *Prediction Studies of Electricity Use of Global Computing in 2030.* Int J Sci Eng Invest, **8**: p. 27-33.
4. Andrae, A.S., 2019b. *Comparison of Several Simplistic High-Level Approaches for Estimating the Global Energy and Electricity Use of ICT Networks and Data Centers.* International Journal, **5**: p. 51.
5. Andrae, A.S., 2019c. *Projecting the chiaroscuro of the electricity use of communication and computing from 2018 to 2030.* Researchgate. net.
6. Andrae, A.S., 2020. *Hypotheses for primary energy use, electricity use and CO2 emissions of global computing and its shares of the total between 2020 and 2030.* WSEAS Transactions on Power Systems.
7. Andrae, A.S. and Edler, T., 2015. *On global electricity usage of communication technology: trends to 2030.* Challenges, **6**(1): p. 117-157.
8. Aslan, J., Mayers, K., Koomey, J.G., and France, C., 2018. *Electricity intensity of Internet data transmission: Untangling the estimates.* Journal of Industrial Ecology, **22**(4): p. 785-798.
9. BBC iPlayer, 2020. *Dirty streaming: The internet's big secret*. https://www.bbc.co.uk/news/av/stories-51742336/dirty-streaming-the-internet-s-big-secret - accessed March 2020.
10. Belkhir, L. and Elmeligi, A., 2018. *Assessing ICT global emissions footprint: Trends to 2040 & recommendations.* Journal of Cleaner Production, **177**: p. 448-463.
11. Berners-Lee, M. and Clark, D., 2013.*The Burning Question: We can't burn half the world's oil, coal and gas. So how do we quit?* : Profile Books.
12. Berners-Lee, M., Howard, D.C., Moss, J., Kaivanto, K., and Scott, W., 2011. *Greenhouse gas footprinting for small businesses—The use of input–output data.* Science of the Total Environment, **409**(5): p. 883-891.
13. Börjesson Rivera, M., Håkansson, C., Svenfelt, Å. and Finnveden, G., 2014. Including second order effects in environmental assessments of ICT. Environmental Modelling & Software, 56, pp.105-115.
14. Cabernard, L., 2019. *Global supply chain analysis of material-related impacts in ICT (MRIO approach)*. http://www.lcaforum.ch/portals/0/df73/DF73-04_Cabernard.pdf - accessed March 2020.
15. Cabernard, L., Pfister, S., and Hellweg, S., 2019. *A new method for analyzing sustainability performance of global supply chains and its application to material resources.* Science of the Total Environment, **684**: p. 164-177.
16. Cambridge Dictionary, 2020. Available from: https://dictionary.cambridge.org/ - accessed March 2020.

17. Cisco, 2020. *Cisco Annual Internet Report (2018–2023).* https://www.cisco.com/c/en/us/solutions/collateral/executive-perspectives/annual-internet-report/white-paper-c11-741490.pdf - accessed February 2020.
18. CoinBundle Team, 2018. Consensus Algorithms. Securing Blockchain Transactions. Medium. https://medium.com/coinbundle/consensus-algorithms-dfa4f355259d#a76e - accessed March 2020.
19. Coroama, V.C., Hilty, L.M., Heiri, E., and Horn, F.M., 2013. *The direct energy demand of internet data flows.* Journal of Industrial Ecology, **17**(5): p. 680-688.
20. Coroama, V.C., Moberg, Å., and Hilty, L.M., 2015. *Dematerialization through electronic media?*, in *ICT Innovations for Sustainability*. Springer. p. 405-421.
21. Court, V., and Sorrell, S., 2020. *Digitalisation of goods: a systematic review of the determinants and magnitude of the impacts on energy consumption.* Environmental Research Letters, **15**(4): p.043001.
22. Department for Business, Energy and Industrial Strategy, 2019. *Greenhouse gas reporting: conversion factors 2019.* UK Government. https://www.gov.uk/government/publications/greenhouse-gas-reporting-conversion-factors-2019 - accessed March 2020.
23. Ercan, M., Malmodin, J., Bergmark, P., Kimfalk, E., and Nilsson, E., 2016. *Life cycle assessment of a smartphone*. in *ICT for Sustainability 2016*. Atlantis Press.
24. Ericsson, 2019. *Exponential data growth – constant ICT footprints.* https://www.ericsson.com/en/reports-and-papers/research-papers/the-future-carbon-footprint-of-the-ict-and-em-sectors - accessed March 2020.
25. ETIP SNET, *Integrating Smart Networks for the Energy Transition: Serving Society and Protecting the Environment.* Vision 2050. https://www.etip-snet.eu/etip-snet-vision-2050/ - accessed March 2020.
26. EU Blockchain, 2020. *Observatory and Forum*. Available from: https://www.euBlockchainforum.eu/ - accessed March 2020.
27. European Commission, 2018a. *European countries join Blockchain Partnership*. Shaping Europe's digital future. Digibyte. https://ec.europa.eu/digital-single-market/en/news/european-countries-join-Blockchain-partnership - accessed March 2020.
28. European Commission, 2018b. *Digital Innovation Hubs.* https://ec.europa.eu/futurium/en/system/files/ged/digital_innovation_hubs_in_digital_europe_programme_final2_december.pdf - accessed March 2020.
29. European Commission, 2019a. *The Internet of Things.* Shaping Europe's digital future. Policy. https://ec.europa.eu/digital-single-market/en/internet-of-things - accessed March 2020.
30. European Commission, 2019b. *Blockchain Unleashed for Climate Action*. Shaping Europe's digital future. Event, Trade Fair and Congress Center of Malaga. https://ec.europa.eu/digital-single-market/en/news/Blockchain-unleashed-climate-action - accessed March 2020.
31. European Commission, 2019c. *On Artificial Intelligence - A European approach to excellence and trust.* https://ec.europa.eu/info/sites/info/files/commission-white-paper-artificial-intelligence-feb2020_en.pdf - accessed March 2020.
32. European Commission, 2019d. *Policy and Investment Recommendations for Trustworthy AI.* Independent High-Level Expert Group on Artificial Intelligence set up by the European Commission. https://www.europarl.europa.eu/cmsdata/196378/AI%20HLEG_Policy%20and

%20Investment%20Recommendations.pdf - accessed March 2020.
33. European Commission, 2020. *Blockchain Technologies*. Shaping Europe's digital future. Policy. https://ec.europa.eu/digital-single-market/en/Blockchain-technologies - accessed March 2020.
34. FCAI, 2020. *The European Commission offers significant support to Europe's AI excellence*. https://fcai.fi/news/the-european-commission-offers-significant-support-to-europes-ai-excellence - accessed March 2020.
35. Fryer, E., 2020. *Does streaming really have a dirty secret?* TechUK. https://www.techuk.org/insights/opinions/item/17020-does-streaming-really-have-a-dirty-secret - accessed March 2020.
36. Galvin, R., 2015. *The ICT/electronics question: Structural change and the rebound effect.* Ecological Economics, **120**: p. 23-31.
37. Gartner, G.I. and Green, I., 2007. *The new industry shockwave, presentation at symposium*. in *ITXPO conference, April*.
38. GeSI, 2008. *Smart 2020: Enabling the low carbon economy in the information age.* A report by The Climate Group on behalf of the Global eSustainability Initiative. https://gesi.org/public/research/smart-2020-enabling-the-low-carbon-economy-in-the-information-age - accessed March 2020.
39. GeSI, 2012. *Smarter 2020: The Role of ICT in Driving a Sustainable Future.* A report by Boston Consulting Group on behalf of the Global eSustainability Initiative. https://gesi.org/report/detail/gesi-smarter2020-the-role-of-ict-in-driving-a-sustainable-future - accessed March 2020.
40. GeSI, 2015. *Smarter 2030: ICT Solutions for 21st Century Challenges.* A report by Accenture Strategy on behalf of the Global eSustainability Initiative. http://smarter2030.gesi.org/downloads.php - accessed March 2020.
41. Gilmore, M., 2018. *Final Report.* Expert and Stakeholder Consultation Workshop. Green ICT - Research and innovation activities (2020-2030). European Commission. https://ec.europa.eu/newsroom/dae/document.cfm?doc_id=50342 - accessed March 2020.
42. Gossart, C., 2015. *Rebound effects and ICT: a review of the literature*, in *ICT innovations for sustainability*. Springer. p. 435-448.
43. GSMA, The Enablement Effect, Technical Report, 2019. https://www.gsma.com/betterfuture/enablement-effect, accessed March 2020.
44. Hewlett, O., 2017. *Ensuring Renewable Electricity Market Instruments Contribute to the Global Low-Carbon Transition and Sustainable Development Goals.* Gold Standard. https://www.goldstandard.org/sites/default/files/documents/gs_recs_position_paper.pdf - accessed March 2020.
45. IEA, 2019. *Global Energy & CO2 Status Report.* https://www.iea.org/geco/emissions/ - accessed March 2020.
46. IPCC, 2018. *Special Report – Global Warming of 1.5 ºC.* https://www.ipcc.ch/sr15/ - accessed March 2020.
47. Jevons, W.S., 1865. *The coal question: Can Britain survive?* In: Flux, A.W. (Ed.), The Coal Question: An Inquiry Concerning the Progress of the Nation, and the Probable Exhaustion of our Coal-Mines. Augustus M. Kelley, New York
48. Kamiya, G., 2020. *Factcheck: What is the carbon footprint of streaming video on Netflix?* Carbon Brief. https://www.carbonbrief.org/factcheck-what-is-the-

carbon-footprint-of-streaming-video-on-netflix - accessed March 2020.
49. Kayali, L., Heikkilä, M., and Delcker, J., 2020. *Europe’s digital vision, explained.* Politico. https://www.politico.eu/article/europes-digital-vision-explained/ - accessed March 2020.
50. Kennelly, C., Berners-Lee, M., and Hewitt, C., 2019. *Hybrid life-cycle assessment for robust, best-practice carbon accounting.* Journal of cleaner production, **208**: p. 35-43.
51. Lannoo, B., Lambert, S., Van Heddeghem, W., Pickavet, M., Kuipers, F., Koutitas, G., Niavis, H., Satsiou, A., Till, M., and Beck, A.F., 2013. *Overview of ICT energy consumption.* Network of Excellence in Internet Science: p. 1-59.
52. Lopez Yse, D., 2019. Your Guide to Natural Language Processing (NLP). Towards Data Science, Medium. https://towardsdatascience.com/your-guide-to-natural-language-processing-nlp-48ea2511f6e1 - accessed March 2020.
53. Lord, C., Hazas, M., Clear, A.K., Bates, O., Whittam, R., Morley, J. and Friday, A., 2015, April. Demand in my pocket: mobile devices and the data connectivity marshalled in support of everyday practice. *In Proceedings of the 33rd Annual ACM Conference on Human Factors in Computing Systems* (pp. 2729-2738).
54. Lövehagen, N., 2020. *What’s the real climate impact of digital technology?* . Ericsson. https://www.ericsson.com/en/blog/2020/2/climate-impact-of-digital-technology - accessed March 2020.
55. Magee, C.L. and Devezas, T.C., 2017. *A simple extension of dematerialization theory: Incorporation of technical progress and the rebound effect.* Technological Forecasting and Social Change, **117**: p. 196-205.
56. Malmodin, J., 2020. The ICT sector’s carbon footprint. Presentation at the techUK conference in London Tech Week on ‘Decarbonising Data’, 2020. https://spark.adobe.com/page/dey6WTCZ5JKPu/ - accessed December 2020.
57. Malmodin, J., 2019. *Energy consumption and carbon emissions of the ICT sector.* Presentation given at Energimyndigheten, Stockholm, 19 Dec 2019.
58. Malmodin, J., Bergmark, P., and Lundén, D., 2013. *The future carbon footprint of the ICT and E&M sectors.* on Information and Communication Technologies: p. 12.
59. Malmodin, J. and Lundén, D., 2018a. *The energy and carbon footprint of the global ICT and E&M sectors 2010–2015.* Sustainability, **10**(9): p. 3027.
60. Malmodin, J. and Lundén, D., 2018b. *The electricity consumption and operational carbon emissions of ICT network operators 2010-2015*. Report from the KTH Centre for Sustainable Communications Stockholm, Sweden.
61. Malmodin, J., Moberg, Å., Lundén, D., Finnveden, G., and Lövehagen, N., 2010. *Greenhouse gas emissions and operational electricity use in the ICT and entertainment & media sectors.* Journal of Industrial Ecology, **14**(5): p. 770-790.
62. Masanet, E., Shehabi, A., Lei, N., Smith, S., and Koomey, J., 2020. *Recalibrating global data center energy-use estimates.* Science, **367**(6481): p. 984-986.
63. Masanet, E., Shehabi, A., Lei, N., Vranken, H., Koomey, J., & Malmodin, J. (2019). Implausible projections overestimate near-term Bitcoin CO 2 emissions. Nature Climate Change, 9(9), 653-654.
64. McMahon, L., 2018. *Is staying online costing the earth?* Technical Report. Policy Connect. https://www.policyconnect.org.uk/appccg/research/staying-online-costing-earth - accessed March 2020.
65. NoCash, 2020. *European Commission about AI: „Europe needs to increase its*

investment levels significantly." Digital Innovation Hubs should provide support to SMEs to understand and adopt AI – at least one innovation hub per Member State. https://nocash.ro/european-commission-about-ai-europe-needs-to-increase-its-investment-levels-significantly-digital-innovation-hubs-should-provide-support-to-smes-to-understand-and-adopt-ai-at-least-one-innovat/ - accessed March 2020.

66. Olivier, J.G.J. and Peters, J.A.H.W., 2019. *Trends in global CO2 and total greenhouse gas emissions: 2019 report.* PBL Netherlands Environmental Assessment Agency, The Hague; 4004. https://www.pbl.nl/sites/default/files/downloads/pbl-2019-trends-in-global-co2-and-total-greenhouse-gas-emissions-summary-ot-the-2019-report_4004.pdf - accessed March 2020.
67. Pineda, A.C. and Faria, P., 2019. *Towards a science-based approach to climate neutrality in the corporate sector.* Science Based Targets report. https://sciencebasedtargets.org/wp-content/uploads/2019/10/Towards-a-science-based-approach-to-climate-neutrality-in-the-corporate-sector-Draft-for-comments.pdf - accessed March 2020.
68. Preist, C., Schien, D., and Blevis, E., 2016. *Understanding and mitigating the effects of device and cloud service design decisions on the environmental footprint of digital infrastructure*. in *Proceedings of the 2016 CHI Conference on Human Factors in Computing Systems*.
69. Preist, C., Schien, D., and Shabajee, P., 2019. *Evaluating Sustainable Interaction Design of Digital Services: The Case of YouTube*. in *Proceedings of the 2019 CHI Conference on Human Factors in Computing Systems*.
70. Sandvine, 2019. *Global Internet Phenomena Report.* https://www.sandvine.com/hubfs/Sandvine_Redesign_2019/Downloads/Internet%20Phenomena/Internet%20Phenomena%20Report%20Q32019%2020190910.pdf - accessed February 2020.
71. Schaffartzik, A., Mayer, A., Gingrich, S., Eisenmenger, N., Loy, C., and Krausmann, F., 2014. *The global metabolic transition: Regional patterns and trends of global material flows, 1950–2010.* Global Environmental Change, **26**: p. 87-97.
72. SBTi (Science Based Targets), 2019. *Science-Based Target Setting Manual.* https://sciencebasedtargets.org/wp-content/uploads/2017/04/SBTi-manual.pdf - accessed March 2020.
73. SBTi (Science Based Targets), 2020. *SBTi Criteria and Recommendations.* https://sciencebasedtargets.org/wp-content/uploads/2019/03/SBTi-criteria.pdf - accessed July 2020.
74. Scott, M., 2019. *New rules to crack down on 'greenwash' in corporate clean energy claims.* Ethical Corporation. http://www.ethicalcorp.com/new-rules-crack-down-greenwash-corporate-clean-energy-claims - accessed March 2020.
75. Shehabi, A., Walker, B., and Masanet, E., 2014. *The energy and greenhouse-gas implications of internet video streaming in the United States.* Environmental Research Letters, **9**(5): p. 054007.
76. Sky, 2020. *Sky Zero.*; Available from: https://www.skygroup.sky/sky-zero - accessed March 2020.
77. Smith, B., 2020. *Microsoft will be carbon negative by 2030.* Microsoft. https://blogs.microsoft.com/blog/2020/01/16/microsoft-will-be-carbon-negative-by-2030/ - accessed March 2020.

78. Sorrell, S., 2009. *Jevons' Paradox revisited: The evidence for backfire from improved energy efficiency.* Energy policy, **37**(4): p. 1456-1469.
79. Sorrell, S., Gatersleben, B. and Druckman, A., 2020. The limits of energy sufficiency: A review of the evidence for rebound effects and negative spillovers from behavioural change. *Energy Research & Social Science*, *64*, p.101439.
80. Stephens, A., 2019. *Net zero: an ambition in need of a definition*. Carbon Trust. https://www.carbontrust.com/news-and-events/insights/net-zero-an-ambition-in-need-of-a-definition - accessed March 2020.
81. Sweney, M., 2020. *Netflix to slow Europe transmissions to avoid broadband overload.* The Guardian. https://www.theguardian.com/media/2020/mar/19/netflix-to-slow-europe-transmissions-to-avoid-broadband-overload - accessed March 2020.
82. The Shift Project, 2019a. *Climate Crisis: The Unsustainable Use of Online Video. The practical case for digital sobriety.* https://theshiftproject.org/en/article/unsustainable-use-online-video/ - accessed March 2020.
83. The Shift Project, 2019b. *Lean ICT: Towards digital sobriety.* https://theshiftproject.org/en/lean-ict-2/ - accessed March 2020.
84. UK Energy Research Council, 2007. *The Rebound Effect: An Assessment of the evidence for economy-wide energy savings from improved energy efficiency.* http://www.ukerc.ac.uk/programmes/technology-and-policy-assessment/the-rebound-effect-report.html - accessed March 2020
85. Van Heddeghem, W., Lambert, S., Lannoo, B., Colle, D., Pickavet, M., and Demeester, P., 2014. *Trends in worldwide ICT electricity consumption from 2007 to 2012.* Computer Communications, **50**: p. 64-76.
86. Vaughan, A., 2019. *Cloud gaming may be great for gamers but bad for energy consumption.* New Scientist. https://institutions.newscientist.com/article/2206200-cloud-gaming-may-be-great-for-gamers-but-bad-for-energy-consumption/ - accessed March 2020.
87. Vereecken, W., Deboosere, L., Simoens, P., Vermeulen, B., Colle, D., Develder, C., Pickavet, M., Dhoedt, B., and Demeester, P., 2009. *Energy efficiency in thin client solutions*. in *International Conference on Networks for Grid Applications*. Springer.
88. Walnum, H.J. and Andrae, A.S., 2016. *The internet: Explaining ICT service demand in light of cloud computing technologies*, in *Rethinking Climate and Energy Policies*. Springer. p. 227-241.
89. Widdicks, K., Hazas, M., Bates, O., and Friday, A., 2019. *Streaming, Multi-Screens and YouTube: The New (Unsustainable) Ways of Watching in the Home*. in *Proceedings of the 2019 CHI Conference on Human Factors in Computing Systems*.
90. Wiebe, K.S., Bjelle, E.L., Többen, J., and Wood, R., 2018. *Implementing exogenous scenarios in a global MRIO model for the estimation of future environmental footprints.* Journal of Economic Structures, **7**(1): p. 20.
